# Validating Machine Learning Models Against the Saline Test Gold Standard for Primary Aldosteronism Diagnosis

**DOI:** 10.1016/j.jacasi.2024.09.010

**Published:** 2024-11-12

**Authors:** Jung-Hua Liu, Wei-Chieh Huang, Jinbo Hu, Namki Hong, Yumie Rhee, Qifu Li, Chung-Ming Chen, Jeff S. Chueh, Yen-Hung Lin, Vin-Cent Wu

**Affiliations:** a Department of Communication, National Chung Cheng University, Chiayi, Taiwan; b Division of Cardiology, Department of Internal Medicine, Taipei Veterans General Hospital, Taipei, Taiwan; c Department of Endocrinology, the First Affiliated Hospital, Chongqing Medical University, Chongqing, China; d Division of Endocrinology and Metabolism, Yonsei University College of Medicine, Seoul, South Korea; e Institute for Innovation in Digital Healthcare, Yonsei University, Seodaemun-gu, Seoul, South Korea; f Department of Biomedical Engineering, National Taiwan University, Taipei, Taiwan; g Department of Urology, College of Medicine, National Taiwan University, Taipei, Taiwan; h Division of Cardiology, Department of Internal Medicine, National Taiwan University Hospital, National Taiwan University College of Medicine, Taipei, Taiwan; i Primary Aldosteronism Center in National Taiwan University Hospital, TAIPAI (Taiwan Primary Aldosteronism Investigation) Study Group, Taiwan; j Division of Nephrology, Primary Aldosteronism Center of Internal Medicine, National Taiwan University Hospital, Taipei, Taiwan

**Keywords:** aldosteronism, Deep Neural Network, feature extraction, feature selection, machine learning, Random Forest, TAIPAI, XGBoost

## Abstract

**Background:**

In this study, we developed and validated machine learning models to predict primary aldosteronism (PA) in hypertensive East-Asian patients, comparing their performance against the traditional saline infusion test. The motivation for this development arises from the need to provide a more efficient and standardized diagnostic approach, because the saline infusion test, although considered a gold standard, is often cumbersome, is time-consuming, and lacks uniform protocols. By offering an alternative diagnostic method, this study seeks to enhance patient care through quicker and potentially more reliable PA detection.

**Objectives:**

This study sought to both develop and evaluate the performance of machine learning models in detecting PA among hypertensive participants, in comparison to the standard saline loading test.

**Methods:**

We used patient data from 3 distinct cohorts: TAIPAI (Taiwan Primary Aldosteronism Investigation), CONPASS (Chongqing Primary Aldosteronism Study), and a South Korean cohort. Random Forest’s importance scores, XGBoost, and deep learning techniques are adopted to identify the most predictive features of primary aldosteronism.

**Results:**

We present detailed results of the model’s performance, including accuracy, sensitivity, and specificity. The Random Forest model achieved an accuracy of 0.673 (95% CI: 0.640-0.707), significantly outperforming the baseline models.

**Conclusions:**

In our discussion, we address both the strengths and limitations of our study. Although the machine learning models demonstrated superior performance in predicting primary aldosteronism, the generalizability of these findings may be limited to East-Asian hypertensive populations. Future studies are needed to validate these models in diverse demographic settings to enhance their applicability.

Primary aldosteronism (PA) is a group of disorders characterized by overt aldosterone production, low plasma renin levels, and relative autonomy, which is relatively nonsuppressible through sodium loading. In cross-sectional and prospective studies, PA represents 5% to 20% of resistant hypertensive patients, but confirmation of PA lacks a standard method and conclusive cutoff point.^1^, ^2^, ^3^ Early diagnosis and proper management of PA are crucial because it is one of the few curable causes of secondary hypertension.^4^

Machine learning (ML) has been successfully used for diagnostic testing and predicting various diseases, including cardiovascular disease^5^ and diabetes.^6^ In this study, we employed the Random Forest model to select and extract clinical features of PA patients, and Random Forest, XGBoost, and deep neural network (DNN) models as prediction models.

The Random Forest model has been used for prediction and as a classifier for facilitating early detection of hypertension.^7^ Random Forest also demonstrated its efficiency at feature selection^8^ and feature extraction^9^ to improve the performance of prediction models. DNN has performed well in complex diagnostics spanning the domains.^10^ In this context, XGBoost performs well in kidney injury prediction.^11^

The saline loading test (SLT) is the standard method recommended by the Endocrine Society to differentiate between essential hypertension (EH) and PA (Supplemental Table 1). However, it is time-consuming, and is less available in diagnostic laboratories. SLT is cumbersome after 4 hours loading of 2-L saline and is avoided in patients with resistant hypertension or congestive heart failure.^12^ In this study, we compared the efficacy of ML and the SLT to detect PA in hypertensive participants, adopting Random Forest, XGBoost, and DNN as prediction models. We validated the initial results from our internal training data set using external international multicenter data sets. Our hypothesis was that artificial intelligence/ML could provide tailored diagnosis for individual patients, even without the cumbersome procedures of confirmatory tests for PA, let alone the difficult switching of antihypertensive medications during the diagnostic procedures.

## Methods

### Study design and participants

Between July 2003 and January 2018, 3,130 hypertensive patients with a suspicion of PA were referred to hypertension clinics and enrolled in the TAIPAI (Taiwan Primary Aldosteronism Investigation) database.^13^, ^14^, ^15^, ^16^, ^17^ The referral conditions included 1 or more of the following characteristics: 1) young hypertension with family history (age of onset <35 years); 2) resistant hypertension; 3) occurrence of a hypertensive emergency; 4) clinical presentation of hypokalemia or metabolic alkalosis, or an aldosterone-to-renin ratio (ARR) >35 (ng/mL/h per ng/dL); or 5) adrenal incidentaloma in hypertensive patients. Thereafter, a holistic database was constructed for quality assurance in 2 medical centers and *5* regional hospitals in different cities^18^ (Supplemental Appendix).

All antihypertensive medications were discontinued for at least 3 weeks before participant enrollment. Diltiazem and/or doxazosin were administered to control markedly high blood pressure when required. As previously described, participants with an initially random ARR >35 were invited to undergo a captopril test for screening.^15^^,^^16^ The Institutional Review Board approved this study at the National Taiwan University Hospital (NTUHN. 200611031R, 202010063).

### Confirmation of PA

Participants with hypertension were diagnosed with primary aldosteronism (PA) based on a postsaline loading plasma aldosterone concentration (PAC) >16 ng/dL.^19^ We validated the base cutoff value of PAC after the saline infusion test after correcting for hypokalemia (Supplemental Appendix). Unilateral primary aldosteronism (uPA) was confirmed using 3 criteria:^20^ 1) confirmed PA; 2) adrenal adenoma or hyperplasia confirmed by a computed tomography (CT) or magnetic resonance imaging scan; and 3) lateralization of aldosterone secretion mostly confirmed by adrenal vein sampling (AVS); and furthermore, uPA was additionally confirmed after adrenalectomy with 4) pathological evidence of a positive CYP11B2-staining specimen.^21^

### Participant profiles

The baseline information of the participants with hypertension was collected and evaluated. This information included age, gender, body weight, height, blood pressure, past medical history, biochemistry profiles for PA screening, confirmation, and subtype identification in accordance with the protocol and aldosteronism consensus of the individual enrolled centers.^20^^,^^22^^,^^23^

### Validation data set from the CONPASS cohort

In total, 643 hypertensive patients who had completed the ARR screening were enrolled in the CONPASS (Chongqing Primary Aldosteronism Study) database at the First Affiliated Hospital of Chongqing Medical University in China.^24^ The screening test was considered positive when the ARR was ≥2.0 ng/dL/mIU/mL (27 pmol/L/mIU/mL).

In addition, PA was confirmed if PAC remained >8 ng/dL (221 pmol/L) after the infusion of 2 L normal saline in the recumbent position or >8.5 ng/dL (235 pmol/L) in the seated position. Participants with a positive result in any confirmatory test underwent an enhanced adrenal CT scan. They also underwent AVS to determine the lateralization of aldosterone hypersecretion if surgery was desired.^23^

### Validation data set from the South Korean cohort

A subset of 60 patients with confirmed PA (n = 30) and EH (n = 30) (mean age 53.3 ± 12.8 years) from the clinical registry of consecutive patients who had visited the endocrinology clinic at Severance Hospital, Seoul, South Korea, between 2016 and 2018 (Severance Hospital IRB number 4-2018-0376) was analyzed. PA was confirmed based on saline infusion test (SIT) results (post-SIT PAC >5 ng/dL). For subtyping, all patients with PA underwent C-arm CT (Axiom Artis dTA, Siemens)-assisted sequential bilateral AVS with nearly 100% successful access to both adrenal veins during continuous intravenous infusion of tetracosactide (Cynacten, Dalim Biotech).^22^

### Extracted features related to clinical categories

The diagnostic codes were categorized into 3 subgroups: EH, uPA, and bilateral primary aldosteronism (biPA). Feature subsets were generated based on combinations of feature selection from the data sets. The records of TAIPAI patients referred to the Hypertension Clinic from a multicenter registration in Taiwan, spanning January 1995 to October 2020, were reviewed. The features we used are those that TAIPAI considers related to primary aldosteronism.^25^ CONPASS and the Korean Cohort provided the patients' feature values from their databases. We did not standardize the continuous variables in our data. During the preprocessing stage, our focus was on handling missing data and converting categorical features (Supplemental Table 2). We transformed categorical variables into one-hot encoded features (Supplemental Table 3), which allowed us to effectively incorporate categorical data into our model. We have 30 features; the total number of feature combinations would be 30 factorial (30!). This number is excessively large, so we opted for a heuristic algorithm^26^ that allows for a limited number of combinations and specifies the number of feature subsets. Although a heuristic algorithm cannot provide an optimal solution, it still offers a practical method to evaluate whether ML can achieve higher accuracy. We selected all features as the first subset, then took all combinations of features with 1 fewer feature from all features as subsets, and continued with the same procedure of reducing 1 feature until 3,000 subsets were generated.

These subsets were derived from the remaining 30 features and were used to train and test our models for differentiating between EH and uPA, EH and biPA, and uPA and biPA. The steps are listed below:

• *Feature selection:* Using Random Forest, we selected the most informative features based on their ability to distinguish between the conditions. The Random Forest models were configured with entropy criteria to evaluate the disorder and included 10 trees to optimize the balance between training time and model accuracy.
• *Model training:* For each condition comparison (EH vs uPA, EH vs biPA, and uPA vs biPA), the top 50 feature subsets with the highest prediction accuracy were identified, resulting in a total of 150 models evaluated. This selection aimed to achieve an optimal balance between accuracy and computational efficiency.
• *Performance evaluation***:** We observed that expanding beyond 50 feature subsets per model comparison did not significantly enhance accuracy and instead increased computational demands. This systematic approach involved selecting numerical intervals of 10 within a range from 10 to 100, allowing us to ascertain the point of diminishing returns in model performance.

Random Forest is not a regression framework and is a nonparametric method with recursive partitioning trees, so Random Forest can avoid collinearity. Given that our model’s features were selected and extracted using Random Forest, the original features do not pose a collinearity problem.^27^

### Statistics

#### Model development and validation

We tested various predictive approaches (detailed in the Supplemental Methods), but none achieved higher accuracy than the Random Forest model, which had an accuracy of 0.673 (95% CI:0.640-0.707). Consequently, we selected Random Forest as the classifier for developing our feature selection, extraction, and prediction models. The analytical process comprised of 5 major stages: preprocessing, feature selection, feature extraction, prediction model construction, and a scoring function.

#### Missing data

In the preprocessing stage, we used −1 as a missing indicator to impute our data. There are various imputation algorithms, and some algorithms are complexly constructed for data sets from specific domains.^28^ Imputing with a constant value can reduce the complexity of model development, and the performance of this method is comparable to that of multiple imputation in some data sets.^29^ Addressing the concern about the imputation strategy, we initially adopted mean value imputation for handling missing numerical data. During our preliminary analyses, we observed that while this approach yielded a mean accuracy of 0.673 (95% CI: 0.640-0.707) in our Random Forest model, it was slightly outperformed by an alternative imputation strategy where missing values were replaced with −1. This latter approach yielded a mean accuracy of 0.682 (95% CI: 0.650-0.716), suggesting a marginal yet notable improvement in model performance.

Moreover, mean imputation, although standard, poses practical challenges, particularly when dealing with new, incoming data. The necessity to recompute means and update imputed values with each new data set not only increases computational overhead but also introduces potential variability without offering a corresponding gain in predictive accuracy. In contrast, imputing with a fixed value of −1 simplifies the process and maintains consistency across varying data sets, which is especially crucial in a model requiring frequent updates.

It's important to note that our models are designed not to accept data with missing values, making some form of imputation indispensable. The choice to use −1 as an imputation value was not arbitrary but rather an informed decision aimed at balancing computational efficiency with model accuracy. This approach aligns with the practical considerations of real-world deployment, where simplicity and reproducibility are paramount.

#### Predictors

The feature extraction stage involved using the selected features to predict the diagnosis and generating new features for constructing the prediction models: Random Forest, DNN, and XGBoost. The final extracted features for groups of EH vs uPA, EH vs biPA, and uPA vs biPA were determined in the prediction model construction stage. Each group had 50 feature subsets, resulting in a total of 150 extracted features for the prediction models. The scoring function stage involved assessing the performance of the constructed prediction models using metrics such as accuracy, precision, recall, and area under the receiver-operating characteristic curve. This analytical process ensured that the selected features were relevant, and the resulting ML models were accurate and robust for predicting different diagnoses of hypertension (see Supplemental Figure 1 for an overview of the process).

#### Feature selection disparities across sites

The results showed disparities in permutation feature importance for the models trained with source data from the TAIPAI data set and were permuted in the 3 diagnosis pairs (ie, EH vs uPA, EH vs biPA, and uPA vs biPA) from each validation site (ie, South Korea and CONPASS) using all features. The top 10 most important permuted features were selected, and the proportion of sites that identified a feature as being in the top 10 was considered the “commonality across sites” to display more important features among all features.

The number of features used to analyze the most important features to illustrate the heterogeneity across the sites comprised the top 10 features. The number of 10 was chosen as a pragmatic choice informed by prior research and practical considerations.^30^ Notedly, the features selected included the characteristics before the confirmation test, namely the screening stage and after withholding medications’ potential to confound the renin-angiotensin-aldosterone system.

Bivariate kernel density estimation (KDE) is a method used to estimate the probability density function of a random variable by considering 2 variables simultaneously. This technique allows for the identification of relationships or patterns between 2 variables, even if the relationship is not strictly statistical in nature (ie, cause-and-effect).^31^ In this context, bivariate KDE plots provide estimated trends of the relation between aldosterone/plasma renin activity (PRA) and the diagnosis of PA.

Statistical analyses were performed using IBM SPSS Statistics for Windows version 22.0 (IBM Corp), R software version 3.2.2 (R Core Team), and Python version 3.7.10 (Python Software Foundation). SPSS and R were applied to proceed the statistics in data set, and Python was used for developing ML models including feature selection, feature extraction, and prediction. We employed scikit-learn, a Python package, to calculate the receiver-operating characteristic (ROC) curve, then obtained AUC function, which integrates the area under the ROC curve plotted with FPR on the x-axis and TPR on the y-axis. The AUC value serves as a scalar quantification of the model's ability to differentiate between classes, with values closer to 1 indicating excellent discrimination and values closer to 0.5 suggesting no better than random guessing.

## Results

### Participant characteristics

In the TAIPAI cohort, significant differences were observed between patients with PA and those with EH. The cohort included 1,624 PA patients and 1,506 EH patients. The PA group was found to be older, shorter in height, have a higher percentage of women, and have a higher prevalence of family history of hypertension and diabetes compared with the EH group.

Furthermore, several biochemical and clinical differences were observed between the 2 groups. In the PA group, compared with the EH group, aldosterone levels were higher, PRA was lower, potassium levels were lower, creatinine levels were higher, and blood pressure was higher. Additionally, serum sodium and calcium levels were higher in the PA group. (Table 1).

**Table 1:** Baseline Characteristics of Primary Aldosteronism Patients From the Derivation TAIPAI Cohort

	All (N = 3,130)	Essential Hypertension (n = 1,506)	Primary Aldosteronism (n = 1,624)	*P* Value
Male	1,529 (48.85)	788 (52.32)	741 (45.63)	0.002
Age, y	51.50 ± 13.99	49.94 ± 15.24	52.95 ± 12.54	<0.001
Family history (parents with HTN)				
Neither	1,271 (40.61)	640 (42.50)	631 (38.85)	0.038
Either	1,356 (43.32)	614 (40.77)	742 (45.69)	0.006
Both	503 (16.07)	252 (16.73)	251 (15.46)	0.331
Body weight, kg	68.39 ± 14.44	68.85 ± 14.62	67.97 ± 14.25	0.152
Height, cm	163.24 ± 8.79	163.79 ± 8.98	162.73 ± 8.59	0.002
Comorbidities				
Myocardial infarction	11 (0.35)	6 (0.40)	5 (0.31)	0.669
COPD	15 (0.48)	9 (0.60)	6 (0.37)	0.356
Diabetes	460 (14.70)	171 (11.35)	289 (17.80)	<0.001
Latency of HTN, y	5 (1-10)	3 (1-8)	5.8 (2-11)	<0.001
At confirmation period				
Aldosterone, ng/dL	44.56 ± 32.68	39.31 ± 27.23	49.43 ± 36.35	<0.001
PRA, ng/mL/h	2.19 ± 4.66	3.65 ± 5.83	0.84 ± 2.56	<0.001
sBP, mm Hg	149.34 ± 22.20	145.16 ± 20.84	153.08 ± 22.72	<0.001
dBP, mm Hg	89.08 ± 14.45	86.45 ± 13.87	91.41 ± 14.56	<0.001
Heart rate	74.46 ± 13.01	74.91 ± 13.50	74.08 ± 12.57	0.151
BUN, mg/dL	15.44 ± 7.14	15.09 ± 5.47	15.66 ± 8.01	0.553
Creatinine, mg/dL	0.98 ± 0.69	0.97 ± 0.71	0.98 ± 0.67	0.007
Na^+^, mmol/L	140.39 ± 3.18	139.85 ± 3.12	140.76 ± 3.16	<0.001
K^+^, mmol/L	3.89 ± 0.61	4.14 ± 0.43	3.65 ± 0.65	<0.001
Ca^+^, mmol/L	2.36 ± 0.51	2.40 ± 0.55	2.33 ± 0.48	<0.001
After confirmation test				
Aldosterone, ng/dL	32.62 ± 24.55	24.88 ± 17.34	37.95 ± 27.23	<0.001
PRA, ng/mL/h	2.69 ± 5.73	4.95 ± 7.31	1.09 ± 3.45	<0.001
Diagnosis				
uPA	1,104 (35.27)	(0.00)	1,104 (67.98)	<0.001
biPA	520 (16.61)	(0.00)	520 (32.02)	<0.001

Values are n (%), mean ± SD or median (Q1-Q3).

APA = aldosterone producing adenoma; ARB = angiotensin II receptor blockers; ARR = aldosterone-renin ratio (ng/dL per ng/mL/h); biPA = bilateral primary aldosteronism; CKD = chronic kidney disease; CVA = cardiovascular accident; dBP = diastolic blood pressure; DM = diabetes mellitus; eGFR = estimated glomerular filtration rate; PAC = plasma aldosterone concentration; PRA = plasma renin activity; sBP = systolic blood pressure.

In the South Korean cohort, the 30 PA patients exhibited significant differences compared with the EH participants, because they were shorter, heavier, and had lower potassium levels (Supplemental Table 4).

Similarly, in the CONPASS cohort, the 324 PA patients differed significantly from the 319 EH participants, because they were thinner and had a lower percentage of diabetes, a longer duration of hypertension, higher aldosterone levels, lower direct renin concentrations, higher blood pressure, higher sodium levels, and lower potassium and calcium levels (Supplemental Table 5).

### Feature selection

**Figure 1:**
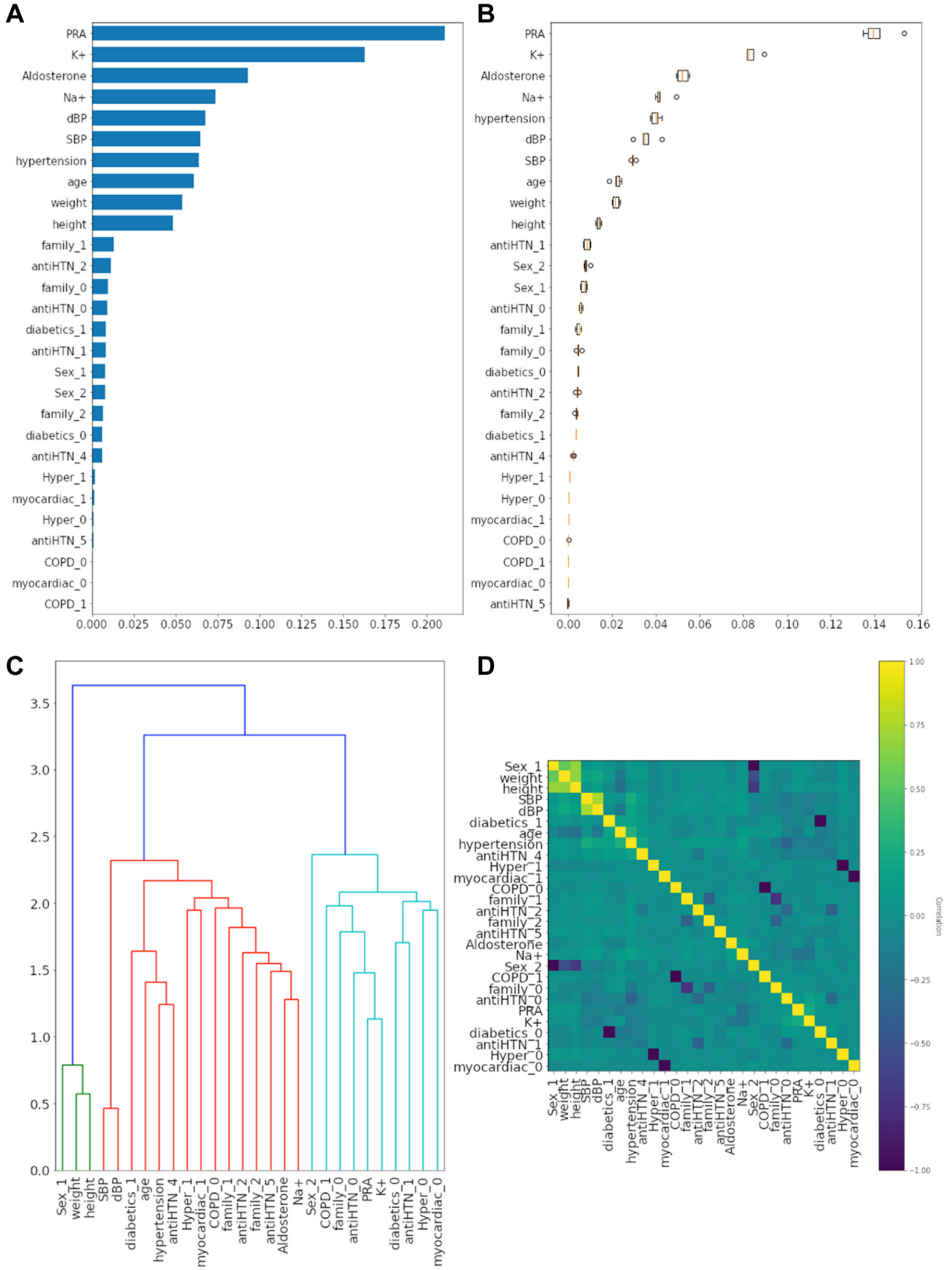
Feature Importance of Random Forest During Feature Selection (A) The bar chart depicting feature importance of the first feature selection subset for predicting essential hypertension and unilateral primary aldosteronism. (B) The box plot of permutation feature importance of the subset. (C) The dendrogram of feature correlations of the subset. (D) The heatmap of feature correlations of the subset. COPD = chronic obstructive pulmonary disease; dBP = diastolic blood pressure; HTN = hypertension; Hyper = hyperthyroidism; myocardiac = myocardial infarction; PRA = plasma renin activity; SBP = systolic blood pressure.

In the analysis of the 150 extracted features derived from the prediction results of combinations of feature selections, PRA and potassium (K+) were the leading features for predicting uPA vs EH (Figures 1A to 1D) and biPA vs EH. Their importance values were significantly larger than those of uPA vs biPA. In the case of uPA vs biPA, the features of PRA, sodium, aldosterone, potassium, and DBP were among the top 5 important features.

Figure 1 depicts the ranking of the highly contributing features in this ML model, with PRA being identified as the most important feature, followed by potassium level (as shown in Figure 1A), with both features having an importance >0.10. The feature importance ranking was conducted based on permutation and relative importance within the models.^32^

The boxplot in Figure 1B demonstrates the permutation feature's importance in the TAIPAI data set, with scattered PRA and restrained potassium levels being identified as significant. Notably, PRA and potassium were the key features that distinguished PA and EH participants. The dendrogram in Figure 1C displays the hierarchical structure of 3 primary clusters among all the participants, and it illustrates the distance between the features and the construction of the relationship between the chosen features. In the dendrogram, the distance between potassium and PRA was shorter than between potassium and aldosterone.

The heatmap in Figure 1D provides the Spearman's correlations between the chosen features, with lighter yellow colors indicating stronger correlations. The heatmap highlights the top 3 correlation sets, namely potassium vs PRA, SBP vs DBP, and sex and weight vs height.

### Feature extraction

The extracted feature “EH-uPA_1” was found to be the most important feature for prediction. The permutation feature analysis derived from the TAIPAI data set also showed that “EH-uPA_1” was the most important permutation feature. The average classification accuracy of the feature extraction method was found to be 95.4% for EH-uPA-biPA and 95.9% for EH-PA of the training data. This accuracy was 10% higher than that of the feature selection method alone. The hierarchical structure revealed 3 main clusters of all the participants, with EH vs uPA and EH vs biPA having shorter distances than uPA vs biPA. (Supplemental Figure 2).

**Figure 2:**
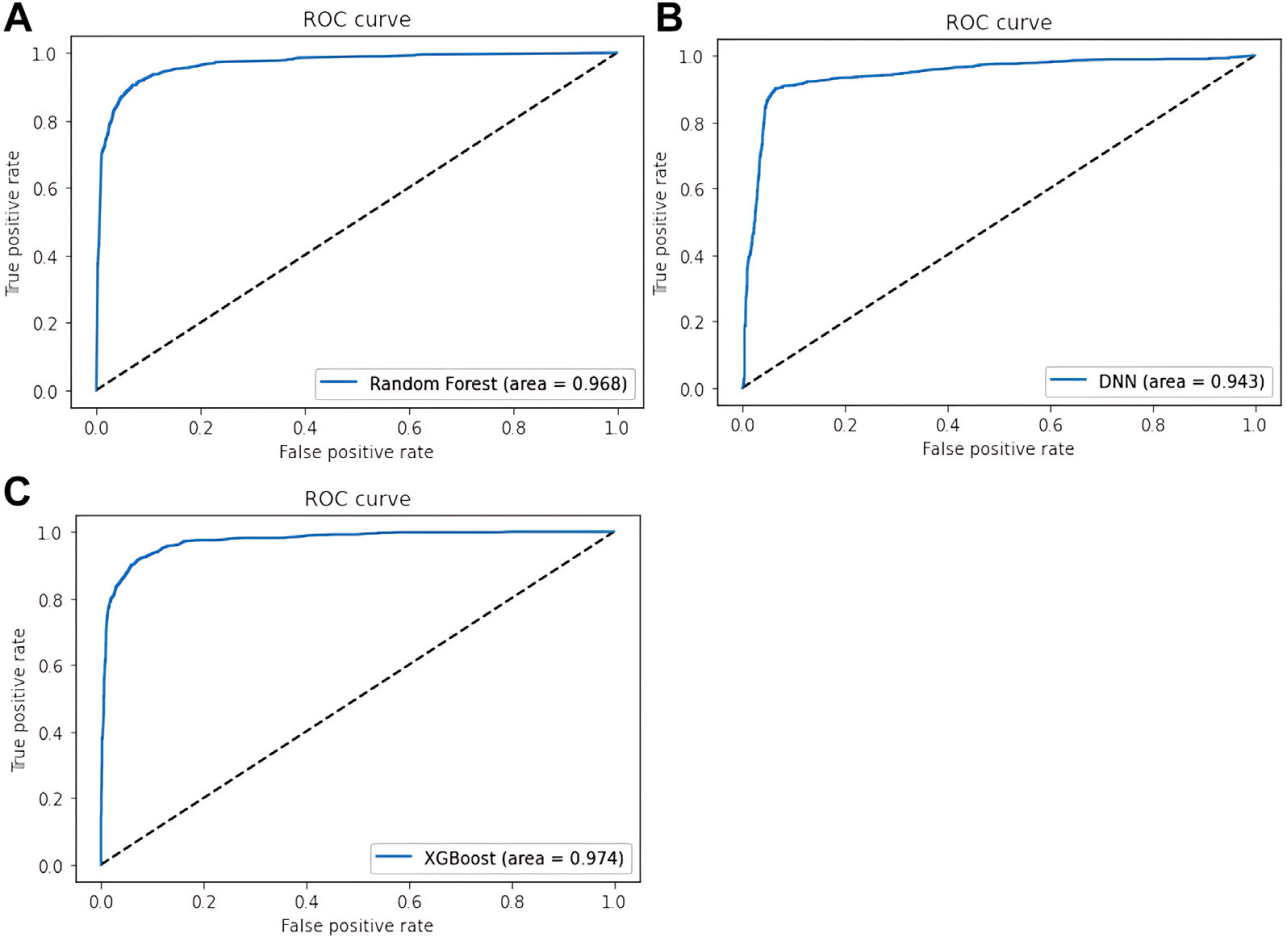
The AUROC of EH-uPA-biPA of Test Set (A) Receiver-operating characteristic (ROC) curve of Random Forest and the AUC value is 0.968. (B) ROC curve of deep neural networks (DNN) and the AUC value is 0.943. (C) ROC curve of XGBoost and the AUC is 0.974.

**Table 2:** The AUROC of EH-uPA-biPA of Test Set

	Random Forest	DNN	XGBoost
Accuracy	0.883 (95% CI: 0.8603-0.9057)	0.887 (95% CI: 0.8646-0.9094)	0.889 (95% CI: 0.8668-0.9112)
AUROC	0.968 (95% CI: 0.955-0.981)	0.943 (95% CI: 0.926-0.90)	0.974 (95% CI: 0.963-0.985)
Recall	0.850	0.870	0.880
F1 score	0.870	0.860	0.880

AUROC = area under the receiver-operating characteristic curve; biPA = bilateral primary aldosteronism; DNN = Deep Neural Network; uPA = unilateral primary aldosteronism.

### Models predicting PA

The accuracy of the tested data sets was 0.887 (95% CI: 0.8646-0.9094) for the DNN model, 0.883 (95% CI: 0.8603-0.9057) for the Random Forest model, and 0.889 (95% CI: 0.8668-0.9112) for the XGBoost model. In the Random Forest model trained from the training set, the AUROC value of 0.968 (95% CI: 0.955-0.981) for all of the participants in the test set demonstrated good performance. The AUROC values of test sets of the DNN model was 0.943 (95% CI: 0.926-0.90) and the AUROC value of the XGBoost model was 0.974 (95% CI: 0.963-0.985) (Figure 2, Table 2).

### Different effects of extracted features in ML models

**Figure 3:**
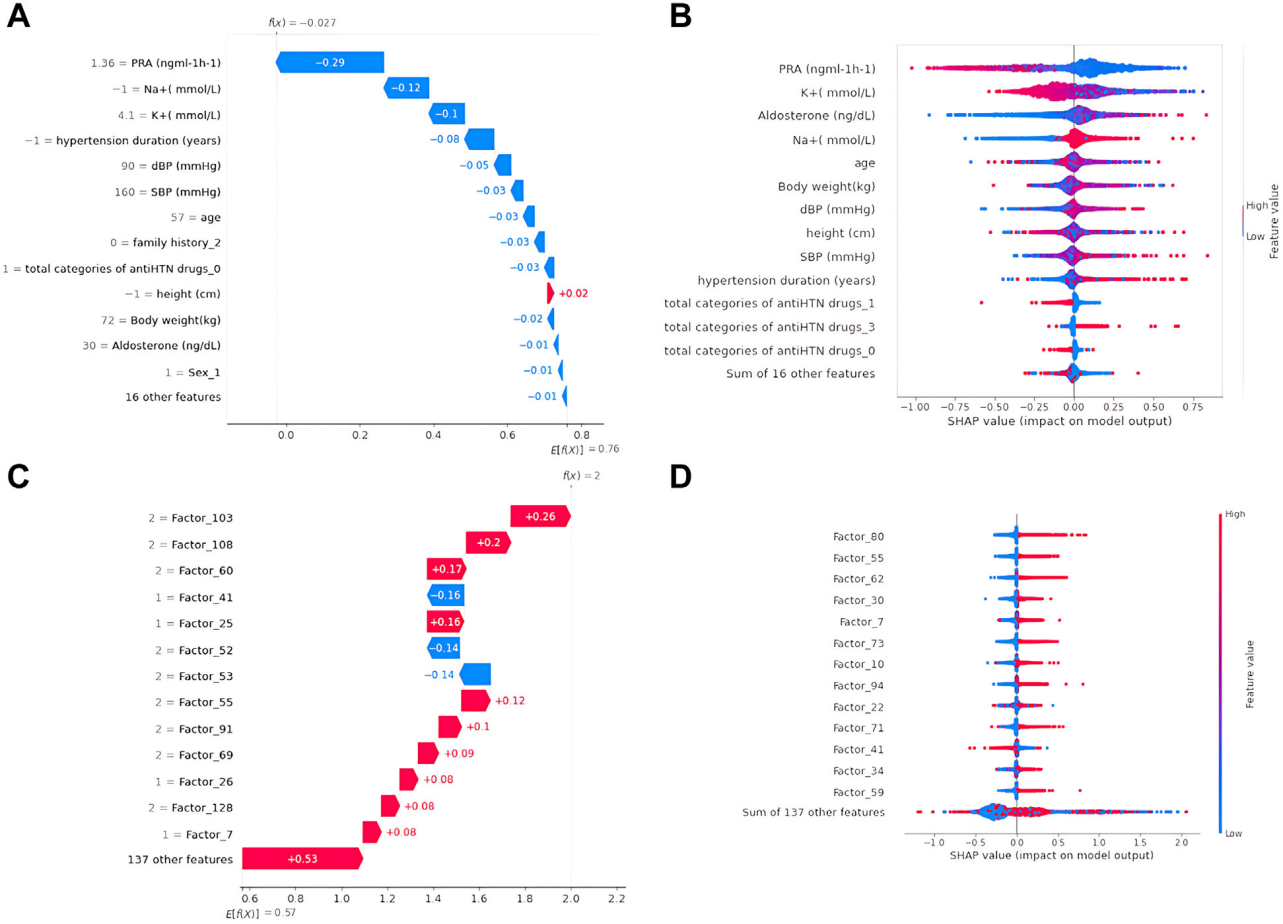
Waterfall Plots of SHAP Values Exploring the Important Expectation (A) The first extracted feature for essential hypertension vs unilateral primary aldosteronism of 1 sample. (B) The first extracted feature for essential hypertension vs unilateral primary aldosteronism of all samples. (C) All extracted features of 1 sample. (D) All extracted features of all samples. Blue symbols depict a negative effect and red depict a positive effect. The number represented the strength of the influence. The x-axis was the impact value. SHAP = Shapley additive explanations; other abbreviations as in Figure 1.

The SHapley Additive exPlanations (SHAP) algorithm was used to explain the XGBoost model prediction (Figure 3), highlighting the features that positively or negatively affect the predictive model (extracted feature mappings are found in the Supplemental Methods).

### Feature selection disparities across sites

**Figure 4:**
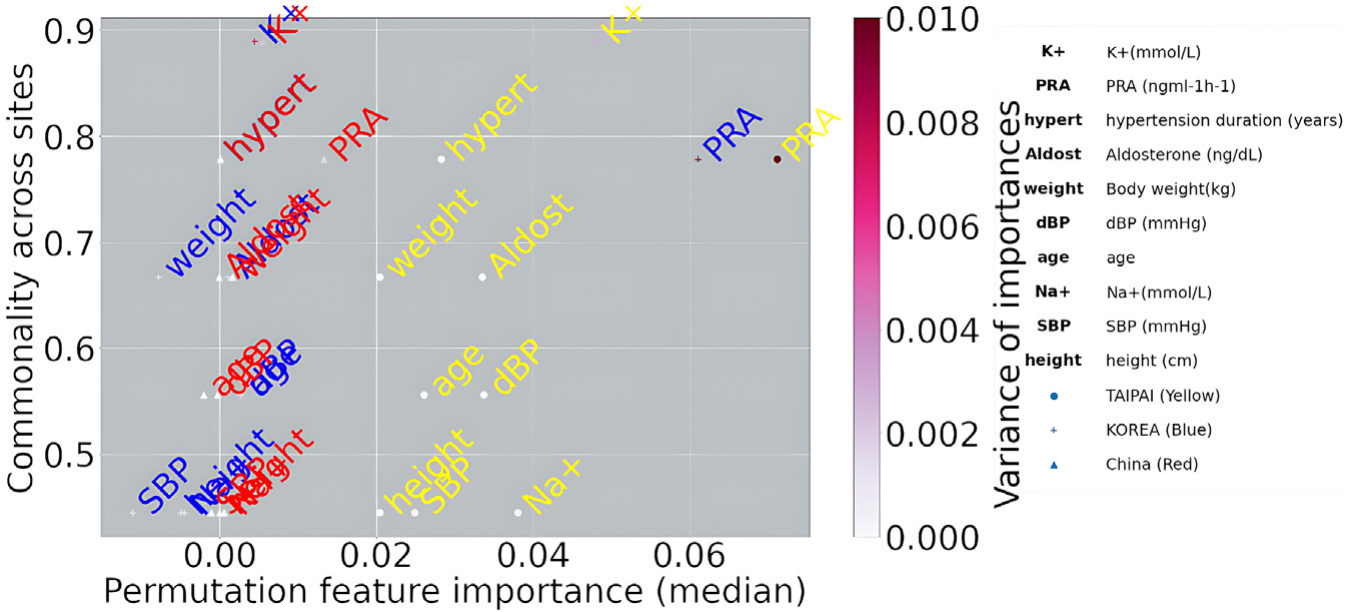
Feature Selection Disparities Across Sites and Permutation Feature Importance Disparities This figure shows feature selection disparities across sites and permutation feature importance disparities for the models trained with source data from the TAIPAI data set and the features permuted in the 3 classifiers (ie, EH vs uPA, EH vs biPA, and uPA vs biPA) at each validation site (ie, South Korea and Mainland China) using all the features (please see Supplemental Methods). Aldost = aldosterone; hypert = hypertension duration (years); other abbreviations as in Figure 1.

In Figure 4, the y-axis represents the proportion of sites that identified the feature as being in the top 10, or “commonality across sites.” The x-axis measures the median of the permutation feature's importance rankings expressed in the pairs of classifiers across the sites. Notably, PRA from the TAIPAI and Korean data sets had the highest specificity. We also draw GAM plots for the most important variables in Supplemental Figure 3.

### Diagnosis sensitivity and specificity of the models

The features trained in the selected models used 10-fold cross-validation to obtain the average accuracy and model performance in terms of AUROC across the various subgroups (ie, uPA and biPA) from the training data set (Supplemental Methods). The models maintained relatively robust performance after evaluating the samples stratified by the PA subgroups uPA and biPA (all *P <* 0.001). For instance, the Random Forest model demonstrated a sensitivity of 0.975 (95% CI: 0.963-0.985) and a specificity of 0.864 (95% CI: 0.849-0.878) in discriminating PA from EH in the TAIPAI data (Supplemental Figure 4A).

### 2D kernel density estimate plots depicted baseline PRA, aldosterone, and SIT

The bivariate analysis explored the density of distribution of baseline PRA and aldosterone between the prediction and actual diagnosis of PA vs EH using the KDE in the screening stage. The results showed that the relationship between baseline aldosterone and standard diagnosis from SIT was consistent (Supplemental Figure 5).

### Probability distribution of the regression mode

In the scatter plots with informative projections,^33^ PA had a higher absolute value of *r* scores compared with EH, which means that PA had a higher negative correlation with PRA (*r* = −0.20) and potassium (*r* = −0.21) (Supplemental Figure 6).

### Decision curve analysis

The y-axis represented the net proportion of participants with PA predicted to benefit without applying a prediction model to participants with good outcomes (see Supplemental Figure 7). The ML model was found to outperform traditional features of potassium and PRA for risk thresholds above 60% of hypertensive participants.^34^

### 2D kernel density estimates the performance of the ML model and SIT

The results showed a consistent relationship between the prediction and standard diagnosis from SIT, with high accuracy along the diagonal line and only a few pseudo-negative samples with multiple missing values (Supplemental Figure 8).

## Discussion

The study presented a novel approach to identifying PA in hypertensive patients using ML models (Central Illustration). The models extracted features from 3 diagnosis pairs, providing a simple and easy way to confirm curable hypertension, if PA is present. The predictors based on the extracted features had a similar accuracy to the SIT, which is considered the gold standard for PA diagnosis. The use of combinations of features improved the accuracy of the models in discriminating between PA and EH. The models' performance was validated using international training data sets, indicating their potential usefulness in clinical practice. SHAP values are presented in Figure 3C, which shows the prediction of factors for 1 patient. Factor 103 has a stronger positive effect on the prediction, whereas Factor 41 has a stronger negative impact. There are no elements in Factor 41 that have been removed from Factor 103. Thus, Factor 103 includes all elements of Factor 41, plus the additional “total categories of antiHTN drugs_0,” indicating that the patient does not use antihypertensive drugs. This case demonstrates how SHAP can help us address the “black box” problem.

**Central Illustration:**
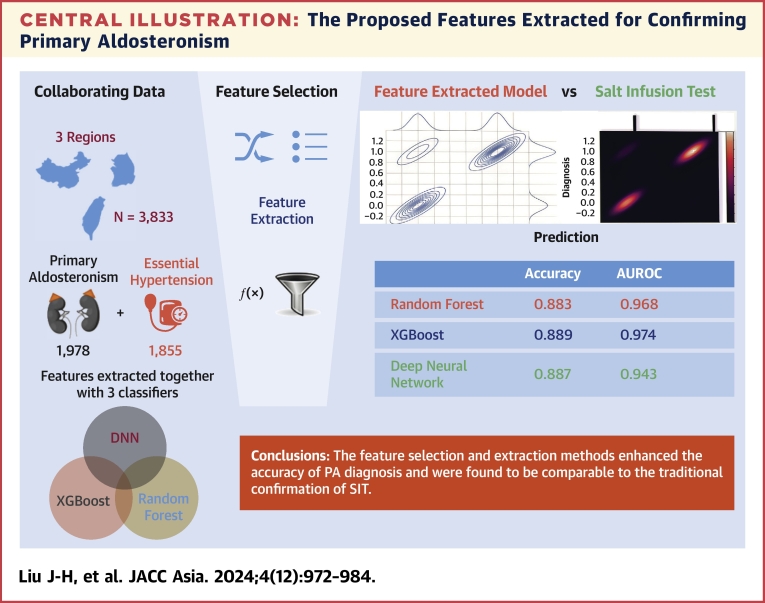
The Proposed Features Extracted for Confirming Primary Aldosteronism This figure demonstrates the validation of machine learning models (Random Forest, XGBoost, and Deep Neural Networks (DNN)) against the saline infusion test (SIT) for diagnosing primary aldosteronism (PA). Data from 3,833 patients (1,978 PA and 1,855 essential hypertension cases) across 3 regions were used, with feature selection and extraction applied to improve model performance. XGBoost achieved the highest accuracy (0.889) and area under the receiver-operating characteristic curve (AUROC) (0.974), followed closely by Random Forest (Accuracy: 0.883, AUROC: 0.968) and Deep Neural Networks (Accuracy: 0.887, AUROC: 0.943). The results indicate that feature selection and extraction methods enhance diagnostic accuracy, making the machine learning models a comparable alternative to traditional SIT diagnosis for PA.

The study also emphasized the importance of accurate screening for PA in hypertensive patients, given the associated costs and risk. Although the sensitivity and specificity of the standard SIT are high, it is often complicated by fluid overload in patients with heart failure,^12^^,^^35^ highlighting the need for alternative approaches. Especially, confirmatory testing of suspected PA requires an extensive medication switch that can be difficult for patients with severe complicated hypertension and/or refractory hypokalemia.^36^ The presented models have the potential to improve the accuracy and efficiency of screening for PA, thereby reducing the burden on health care systems and improving patient outcomes. The ML models could optimize the allocation of scarce health care resources effectively^37^ by improving disease diagnosis, aiding in treatment selection, and enhancing clinical laboratory testing by leveraging large data sets.^38^

For external validation, we have trained our models using the TAIPAI data set and validated our XGBoost model with data sets from South Korea and CONPASS. We chose XGBoost as the model for external validation because it performed well in our initial experiments. We have added these results to Supplemental Table 6 and the ROC figures to Supplemental Figure 9. In our external validation, the accuracy of the South Korean data set is 0.667 (95% CI: 0.548-0.786), and the AUROC is 0.768 (95% CI: 0.648-0.888). For the CONPASS data set, the accuracy is 0.629 (95% CI: 0.592-0.666), and the AUROC value is 0.775 (95% CI: 0.739-0.811). This performance is worse than models that include both the South Korean and CONPASS data sets. However, its discriminative ability is still superior to that of other external validation studies on primary aldosteronism. Sam et al^39^ conducted external validations of 6 clinical prediction models for unilateral primary aldosteronism, finding that the range of C-statistics for ROC curves varied from 0.60 to 0.72. The training sample sizes for these 6 models were fewer than 300. The sample size and characteristics of the validation cohort could be one reason for the poor performance. Including more data sets for training could improve the models, as demonstrated by our inclusion of the CONPASS and South Korean data sets.

### Comparison of features-extracted models with reported ML

Limited studies have utilized ML techniques to predict the diagnosis of PA. For instance, Kaneko et al^40^ applied various ML algorithms to analyze a relatively small sample size of 253 participants. Their algorithm achieved an accuracy of 95.7% and an AUC of 99.0%. However, the possibility of overfitting issues in a small sample size from a single center raises concerns about the generalizability of their results. In contrast, our study has several advantages, including the largest sample size and the ability to discriminate well among 3 different international sites.

Compared with the study by Buffolo et al,^41^ our model showed better performance with a larger AUC value. While they also applied a Random Forest model to predict PA with a sample size of over 4,000, their AUC values from the validation cohort were only 0.739 for positive screening tests, 0.796 for PA, and 0.882 for uPA. Our approach, on the other hand, showed good discrimination to 3 different international sites and had similar accuracy to the standard SIT test.

This study allows clinicians to obtain the probability of PA through routine blood tests and medical records, avoiding the need for complicated physiological tests. Early identification of aldosterone-producing adenoma using ML models can prevent misinterpretation of laboratory results, ultimately reducing health care costs and waiting times.

### Study strengths and limitations

In summary, our study has demonstrated superior performance compared with previous studies by utilizing feature selection and extraction to overcome the challenges of large cohorts from East-Asian Patients. We were able to reduce the noise between features and identify the most important ones for accurate diagnosis. Notably, our models do not require imaging studies, especially in some medically underserved East-Asian patients. This is particularly convenient for clinical diagnosis and aligns with the HISTADO criteria for nonclassical PA. ^42^

The study has several limitations that need to be considered. First, the data sets used in the study are limited to Asian countries, which means that further validation is required to confirm whether the models can perform well in more complex cohorts worldwide. Sample size determination in ML takes into account various factors, including model complexity and overfitting.^43^ In our study, it is essential to emphasize that our data sets originate from 3 different centers, adding an extra layer of complexity. This multicenter aspect introduces additional variability, which can affect the sample size requirements.

Second, although our models can predict EH and PA, their performance in subgroup separation is poor. Although we can identify which features are more important by analyzing the relationship between prediction probability and feature values, such as the high indexes of aldosterone and potassium, ML cannot explain how the important features affect the disease. Due to a large cohort and validation from international collaborations, our sample number was large enough to contain different variants to avoid overfitting issues. Further investigation is needed to determine which features caused poor performance in distinguishing uPA and biPA. Third, there may have been potential work-up bias in this international cohort study caused by different diagnosis protocols. However, the results of KDE plots showed that the density was high, suggesting limited bias between enrolled centers. Fourth, although we showed that potassium and PRA were the leading feature factors in discriminating between PA and EH, the other feature factors in our ML model were still enriched in a black box and require further explainable ML tools. To address this, we have used SHAP in our studies to explain which features are important for our models. Fifth, we did not test feature selections with all classifiers other than the ones previously mentioned, because it would be time-consuming and require a huge effort, but there may be other classifiers that can produce more accurate results. We will build a web-based application to explore our and other classifiers. The backend infrastructure serves as the repository for these models, while a user-friendly frontend website has been meticulously designed to enable health care professionals. The standout feature of our system is its ability to provide immediate predictions for PA, thereby significantly expediting the diagnosis and decision-making process. Sixth, we employed constant value imputation to simplify our model development process. We observed that this imputation method yielded lower accuracy with other models but performed well with Random Forest, DNN, and XGBoost. In future work, we will explore various imputation methods to examine their impact on our models and on traditional statistical models within our data sets.

## Conclusions

In this study, we provide features-extracted models that adopt combinations of feature selections and 3 classifiers (EH, uPA, and biPA) to identify PA at the confirmation stage if curable hypertension can be distinguished between PA and EH from East-Asian patients. The feature selection and extraction methods enhanced the accuracy of PA diagnosis and were found to be comparable to the traditional confirmation of SIT. To achieve generalization and reliability, our models are trained from 3 areas to generalize the ability of prediction and make our model more reliable across sites.

Perspectives **COMPETENCY IN MEDICAL KNOWLEDGE:** The ensemble of feature selection and extraction with ML models can provide the confirming primary aldosteronism. ML models are comparable accuracy to the traditional SIT standard.

**TRANSLATIONAL OUTLOOK:** Further research is necessary to create higher accurate prediction models with new algorithms and to explore the efficacy of more features and data.

## Funding Support and Author Disclosures

The authors have reported that they have no relationships relevant to the contents of this paper to disclose.
