# Supplementary material for: Validating Machine Learning Models Against the Saline Test Gold Standard for Primary Aldosteronism Diagnosis
Source: JACC Asia. 2024 Nov 12;4(12):972–84. doi: 10.1016/j.jacasi.2024.09.010 (PMC11712017; doi:10.1016/j.jacasi.2024.09.010)
Supplement: Supplemental Material [file mmc1.docx]

**Validating Machine Learning Models Against the Saline Test Gold Standard for Primary Aldosteronism Diagnosis**

**Short title**: Machine learning predicting primary aldosteronism

Jung-Hua Liu, PhD^1^, Wei-Chieh Huang, MD^2^, Jinbo Hu, MD, PhD^3^, Namki Hong, MD, PhD^4,9^, Yumie Rhee, MD, PhD ^4^, Qifu Li, MD, PhD^3^, Chung-Ming Chen, PhD^5^, Jeff S Chueh, MD, PhD^6^, Yen-Hung Lin, MD, PhD^7, #^, Vin-Cent Wu MD, PhD^8, #^

**Supplemental methods**

We used a criteria of autonomous excess aldosterone production evidenced with ARR > 35 ng/dL per ng/ml/hr after captopril test, TAIPAI score > 60%, (Wu, Yang et al. 2011) and post-saline loading plasma aldosterone concentration (PAC) > 16 ng/dL to identify PA patients who received MRA treatment.

We have also done a systemic review of the performances of the saline infusion test. (table). The post saline loading aldosterone level was 14.5± 4.8ng/dL, while our criteria was within one standard deviation.

We removed data with mis-labeled diagnosis and gender in Chongqing Primary Aldosteronism Study (CONPASS) dataset, then split every dataset into around 80% train data and 20% test data. The complete training dataset contains 3051 records and test dataset contains 767 ones. In training dataset, there are 504 CONPASS records, 50 Korea ones, and 2497 TAIPAI records. The complete test dataset contains 124 CONPASS records. 10 Korea records, and 633 TAIPAI records.

We have expanded upon our initial exploration of various modeling approaches during the early stages of the study. Specifically, we tested several models, including Linear Regression, Logistic Regression, k-Nearest Neighbors (kNN), Decision Tree, and Naïve Bayes Classifier. For kNN and Decision Tree, we adjusted key parameters such as the k-value and maximum depth, respectively. The Linear and Logistic Regression models were trained with default parameters using the scikit-learn Python library. To ensure robustness, each model was trained and tested five times, and the mean accuracies were calculated.

The results were as follows:

- **Linear Regression:** Mean accuracy of 0.120 (95% CI [0.097, 0.144])
- **Logistic Regression:** Mean accuracy of 0.556 (95% CI [0.521, 0.591])
- **k-Nearest Neighbors (kNN):** Mean accuracy varied with k values, ranging from 0.510 (95% CI [0.475, 0.546]) at k = 3 to 0.543 (95% CI [0.508, 0.579]) at k = 10.
- **Decision Tree:** Mean accuracy ranged from 0.558 (95% CI [0.523, 0.593]) with a maximum depth of 100 to 0.596 (95% CI [0.561, 0.630]) with a maximum depth of 10.
- **Naïve Bayes Classifier:** Mean accuracy of 0.315 (95% CI [0.282, 0.348]).

Despite these efforts, the accuracies of these models were consistently lower than that of the Random Forest model, which achieved a mean accuracy of 0.673 (95% CI [0.640, 0.707]). Due to its superior performance, we selected Random Forest as the primary classifier for developing feature selection, extraction, and prediction models in our study.

Hyperparameters in our models:
XGBoost:

| Name | Value |
| --- | --- |
| max_depth | 27 |
| learning_rate | 0.01, 0.1, 0.02 |
| objective | binary:logistic |
| scoring | neg_mean_squared_error |

DNN: There are 5 hidden layers, and the numbers of nodes are 1000, 1000, 1000, 50, 50.

| Name | Value |
| --- | --- |
| activation | ReLU for hidden layers, Softmax for input and out layers |
| loss_fn | Sparse Categorical Crossentropy |
| optimizer | adam |
| metrics | sparse_categorical_accuracy |

Random Forest:

| Name | Value |
| --- | --- |
| max_depth | 2 |

The following definition are the methods we used in this paper:

1. Feature Selection: Feature selection is the process of choosing a subset of relevant features (variables or attributes) from a larger set of data features. It aims to improve model performance by reducing dimensionality, eliminating irrelevant or redundant information, and enhancing model interpretability.
2. Feature Extraction: Feature extraction involves transforming or creating new features from the original data to capture essential information or patterns. This technique is often used to reduce dimensionality, improve model performance, and reveal latent structures in the data.
3. Random Forest: Random Forest is an ensemble machine learning algorithm that builds multiple decision trees and combines their predictions to make more accurate and robust predictions. It's known for its versatility, ability to handle various data types, and resistance to overfitting.
4. DNN (Deep Neural Network): A Deep Neural Network is a type of artificial neural network with multiple hidden layers between the input and output layers. DNNs are capable of learning complex hierarchical representations from data, making them suitable for tasks like image recognition, natural language processing, and more.
5. XGBoost (Extreme Gradient Boosting): XGBoost is a powerful gradient boosting machine learning algorithm known for its efficiency and effectiveness in predictive modeling. It optimizes decision tree models sequentially, combining their outputs to achieve superior performance in tasks like classification and regression.

**Performance metrics**

The predictive performance was evaluated using kernel density estimation (KDE), the area under the receiver operating characteristic curve (AUROC), and the area under the precision-recall curve (AUPRC).

**Feature selection** disparities across sites and permutation feature importance disparities for the models trained with source data from the TAIPAI dataset and the features permuted in the three classifiers (i.e., EH vs. uPA, EH vs. biPA, and uPA vs. biPA) at each validation site (i.e., South Korea and Mainland China) using all the features. Each dot corresponded to one of the most important permuted features ranked among the top 10 by at least four of the pairs of Random Forest classifiers and sites; the y-axis measured the proportion of sites that identiﬁed the feature as being in the top 10, or “commonality across sites”; the x -axis measured *the median* of permutation feature importance rankings in the pairs of classifiers across sites. The arbitrary cutoff used to analyze the most important features to illustrate heterogeneity across sites was the top 10 features. E.g. PRA comes from TAIPAI samples.

**Acknowledgements**

The authors greatly appreciate the Second Core Lab in National Taiwan University Hospital for technical assistance. This study was supported by Ministry of Science and Technology (MOST) of the Republic of China (Taiwan) [grant number, MOST 107-2314-B-002-026-MY3, 108-2314-B-002-058, 110-2314-B-002-241, 110-2314-B-002-239], National Science and Technology Council (NSTC) [grant number, NSTC 109-2314-B-002-174-MY3, 110-2314-B-002-124-MY3, 111-2314-B-002-046, 111-2314-B-002-058], National Health Research Institutes [PH-102-SP-09], National Taiwan University Hospital [109-S4634, PC-1246, PC-1309, VN109-09, UN109-041, UN110-030, 111-FTN0011 ] Grant MOHW 110-TDU-B-212-124005 and Mrs. Hsiu-Chin Lee Kidney Research Fund.

¶ Patients were withdrawn from antihypertensive medications at least 21 days before the study, with the exception of calcium antagonist or alpha-blockers.

Data were provided as the mean values ± standard deviation

Note: To convert potassium in mmol/L to mEq/L, multiple by 1; BUN in mg/dL to mmol/L, multiple by 0.375; eGFR in mL/min to mL/s, multiple by 0.01667; PAC in ng/dL to nmol/L, multiple by 0.02774; PRA in ng/mL/hr to ng/(Lxs), multiple by 0.2778.

‡ Obtained after sodium infusion test and hold drugs that will interfere the renin-angiotensin system

§ Tumor size was measured using computed tomography or magnetic resonance imaging.

**Abbreviations:** aldost, aldosterone; biPA, bilateral primary aldosteronism; EH,essential hypertension; hypert,hypertension; K,potassium; Na,sodium; PA,primary aldosteronism; PRA,plasma renin activity, SBP,systolic blood pressure; uPA, unilateral primary aldosteronism.

**Supplemental Table 1.** The performance of saline infusion test

| years | authors | Number of subjects | Post-test aldosterone threshold (pmol/L) | Post-test aldosterone threshold (ng/dL) | Diagnostic accuracy | Diagnostic criteria |
| --- | --- | --- | --- | --- | --- | --- |
| 2001 | Agharazii M | 44 | 246 | 19.07 | Se 100% | CT/scintigraphy/AVS/surgery |
| 2006 | Giacchetti G | 118 | 196 | 15.19 | Se 88%Spe 100% | AVS/surgery outcome |
| 2006 | Mulatero P | 98 | 139 | 10.78 | Se 88%Spe 88% | FST positivity |
| 2007 | Rossi GP | 120 | 196 | 15.19 | Se 82%Spe 75% | CT/AVS |
| 2012 | Nanba K | 57 | 170 | 13.18 | Se 60% | Scintigraphy/AVS/surgery |
| 2012 | Willenberg HS | 33 | 88 | 6.82 | Se 82%Spe 92% | AVS/surgery outcome |
| 2014 | Ahmed AH | 66 | 165 seated 140 supine | 12.8 seated 10.9 supine | Se 96% seated Se 33% supine | FST positivity |
| 2016 | Cornu E | 199 | 139 | 10.78 | 29% of false negative | AVS/surgery outcome |
| 2018 | Song Y | 236 | 222 | 17.21 | Se 85%Spe 92% | FST positivity |
| 2018 | Meng X | 164 | 310 | 24.03 | Se 90.4%Spe 95.9% | AVS/Surgery outcome |

### Abbreviations: AVS=adrenal venous sampling; FST=Fludrocortisone suppression test; SIT=Saline infusion test.

According to the consensus of Taiwan Aldosteronism Society(Wu, Hu et al. 2017), one positive confirmation test is considered adequate to make the diagnosis of primary aldosteronism. Compared with the criteria of other society that need one more confirmation study to diagnosis PA(Nishikawa, Omura et al. 2011), our survey criteria could have high sensitivity, not to say identified with high severity patients. (Wu, Chang et al. 2009)

***Unilateral PA***

Unilateral PA (aldosterone producing adenoma) was identified on the basis on the following four criteria^1^: (1) Confirmed PA; (2) an adrenal adenoma or hyperplasia evidenced with a CT or MRI scan [6]; (3) lateralization of aldosterone secretion with adrenal vein sampling (AVS) on the imagine finding side;

Aldosterone producing adenoma/ nodules (APA/APN) is further confirmed after adrenalectomy:

(4) pathologically proven a CYP11B2 adenoma or (multiple) aldosterone-producing nodule / micronodule at immunohistochemistry according to the HISTALDO consensus^5, 6^ after adrenalectomy, and subsequent emergence of biochemical correction.

**Selectivity and lateralization indices of AVS without stimulation tests**

The selectivity index (SI) is defined as the ratio of the sampled cortisol concentration of each adrenal vein to that of the peripheral vein. The lateralization index (LI) is defined as the ratio of the aldosterone/cortisol concentration on the dominant side to that on the contralateral side. Successful AVS is defined as an SI value ≥2.0 bilaterally. After confirming successful bilateral AVS, lateralization of the PA was determined by an LI value ≥2.0.

**Supplemental Table 2.** Baseline characteristics of the participants in the validated South Korea cohort¶

| Predictors | ALL | Essential Hypertension | Primary Aldosteronism | *p* |
| --- | --- | --- | --- | --- |
|  | **60** | **(n=30)** | **(n=30)** |  |
| Gender (M) | 19 (31.7%) | 6 (20.00%) | 13 (43.33%) | 0.052 |
| Age (y/o) | 51.02±11.53 | 52.96±11.21 | 49.07±11.72 | 0.193 |
| Family history |  |  |  | 0.858 |
| Neither | 32 (53.33%) | 15 (50.00%) | 17 (56.67%) |  |
| Either | 22 (36.67%) | 12 (40.00%) | 10 (33.33%) |  |
| Both | 6 (10.00%) | 3 (10.00%) | 3 (10.00%) |  |
| Body weight (Kg) | 67.96±17.95 | 60.58±9.90 | 75.35±21.10 | 0.001 |
| Height (cm) | 163.06±8.97 | 159.55±7.49 | 166.57±9.06 | 0.002 |
| Comorbidities |  |  |  |  |
| Diabetes | 6 (10.00%) | 3 (10.00%) | 3 (10.00%) | 0.999 |
| Latency of HTN (yr) median [IQR] | 1 [0-2.5] | 1 [0-2] | 1 [1-3] | 0.146 |
| At screening period |  |  |  |  |
| PAC (ng/dL) (ng/dL) | 25.44±12.41 | 23.19±8.93 | 27.69±14.95 | 0.163 |
| PRA (ng/mL/hr) | 0.61±0.69 | 0.68±0.91 | 0.54±0.36 | 0.414 |
| SBP (mmHg) | 147.30±21.38 | 144.06±22.83 | 150.53±19.68 | 0.245 |
| dBP (mmHg) | 90.10±14.18 | 88.36±13.89 | 91.83±14.49 | 0.348 |
| Heart rate | 72.23±9.47 | 72.63±11.83 | 72.23±9.47 | 0.886 |
| BUN (mg/dL) | 13.15±3.19 | 13.02±4.22 | 13.15±3.19 | 0.899 |
| Creatinine (mg/dL) | 0.74±0.15 | 0.71±0.13 | 0.77±0.17 | 0.123 |
| Na+ ( mmol/L) | 141.93±2.17 | 141.90±1.66 | 141.97±2.62 | 0.907 |
| K+ ( mmol/L) | 3.97±0.51 | 4.26±0.33 | 3.68±0.49 | <0.001 |
| Ca+ ( mmol/L) | 9.34±0.45 | 9.31±0.35 | 9.37±0.55 | 0.656 |
| After confirmation test |  |  |  |  |
| PAC (ng/dL) (ng/dL) | 8.35±8.71 | 2.90±1.18 | 13.80±9.57 | <0.001 |
| Diagnosis |  |  |  |  |
| uPA | 10 (16.67%) | NA | 10 (33.33%) | NA |
| BiPA | 20 (33.33%) | NA | 20 (66.67%) | NA |

### Abbreviations: PAC= plasma aldosterone concentration; PRA=plasma renin activity; SBP=systolic blood pressure; dBP=diastolic blood pressure; BUN=blood urea nitrogen; uPA= unilateral primary aldosteronism; biPA= bilateral primary aldosteronism.

**Supplemental Table 3.** Baseline characteristics of the participants in the validated CONPASS cohort¶

| Predictors | ALL | Essential Hypertension | Primary Aldosteronism | *p* |
| --- | --- | --- | --- | --- |
|  | **643** | **(n=319)** | **(n=324)** |  |
| Gender (M) | 243 (37.79%) | 117 (36.68%) | 126 (38.89%) | 0.563 |
| Age (y/o) | 48.14±12.61 | 49.29±13.58 | 47.01±11.50 | 0.034 |
| Family history |  |  |  |  |
| Neither | 301 (46.81%) | 156 (48.90%) | 145 (44.75%) | 0.292 |
| Either | 258 (40.12%) | 121 (37.93%) | 137 (42.28%) | 0.260 |
| Both | 84 (13.06%) | 42 (13.17%) | 42 (12.96%) | 0.939 |
| Body weight (Kg) | 65.16±12.26 | 66.51±12.53 | 63.84±11.87 | 0.009 |
| Height (cm) | 161.49±8.04 | 161.39±8.06 | 161.59±8.02 | 0.670 |
| Comorbidities |  |  |  |  |
| Myocardial infarction | 12 (1.87%) | 3 (0.94%) | 9 (2.78%) | 0.085 |
| COPD | 2 (0.31%) | 2 (0.63%) | 0 (0.00%) | 0.153 |
| Diabetes | 122 (18.97%) | 75 (23.51%) | 47 (14.51%) | 0.004 |
| Latency of HTN (yr) median [IQR] | 4 [1-10] | 3 [1-9] | 5 [1-10] | 0.001 |
| At screening period |  |  |  |  |
| PAC (ng/dL) (ng/dL) | 24.79±22.30 | 15.21±8.45 | 34.22±27.17 | <0.001 |
| PRA (ng/mL/hr) | 14.87±27.97 | 2.84±4.65 | 26.71±35.35 | <0.001 |
| SBP (mmHg) | 152.03±19.39 | 150.51±19.49 | 153.52±19.20 | 0.016 |
| dBP (mmHg) | 93.15±13.81 | 91.86±13.98 | 94.43±13.55 | 0.019 |
| BUN (mg/dL) | 14.17±4.36 | 14.52±4.30 | 13.87±4.40 | 0.127 |
| Creatinine (mg/dL) | 0.80±0.26 | 0.79±0.28 | 0.80±0.24 | 0.372 |
| Na+ ( mmol/L) | 143.01±2.86 | 142.40±2.70 | 143.60±2.89 | <0.001 |
| K+ ( mmol/L) | 3.61±0.64 | 3.96±0.41 | 3.27±0.64 | <0.001 |
| Ca+ ( mmol/L) | 2.28±0.12 | 2.31±0.12 | 2.26±0.12 | <0.001 |
| After confirmation test |  |  |  |  |
| PAC (ng/dL) | 17.92±22.51 | 16.39±16.93 | 19.21±25.83 | <0.001 |
| PRC(uIU/ml) | 4.72±7.89 | 5.85±9.13 | 3.87±6.70 | <0.001 |
| Diagnosis |  |  |  |  |
| uPA | 241 (37.48%) | NA | 241 (74.38%) | NA |
| BiPA | 83 (12.91%) | NA | 83 (25.62%) | NA |

**Note:** Data in Supplementary Tables are presented as the mean [confidence interval] for normally distributed data and median [interquartile range] for non-normally distributed data;

**Abbreviations:**

APA=aldosterone producing adenoma; ARB=angiotensin II receptor blockers; ARR=aldosterone-renin ratio (ng/dL per ng/mL/h); CONPASS= Chongqing Primary Aldosteronism Study; CKD=chronic kidney disease; CVA=cardiovascular accident; dBP=diastolic blood pressure; DM=diabetes mellitus; eGFR=estimated glomerular filtration rate; mBP=mean blood pressure; IHA=idiopathic hyperaldosteronism; PAC=plasma aldosterone concentration; PRA=plasma renin activity; SBP=systolic blood pressure; ¶=patients were withdrawn from anti-hypertensive medications at least 21 days before the study, with the exception of calcium antagonist or alpha-blockers; data were provided as the mean values± standard deviation; to convert potassium in mmol/L to mEq/L, multiplied by 1; BUN in mg/dL to mmol/L, multiplied by 0.375; eGFR in mL/min to mL/s, multiplied by 0.01667; PAC in ng/dL to nmol/L, multiplied by 0.02774; PRA in ng/mL/hr to ng/(Lxs), multiplied by 0.2778; uPA= unilateral primary aldosteronism; biPA= bilateral primary aldosteronism**.**.

**Supplemental Table 4.** Features used in the random forest model

| Features | Data Type |
| --- | --- |
| Gender | categorical |
| Age (y/o) | numerical |
| Family history | categorical |
| Body weight (Kg) | numerical |
| Height (cm) | numerical |
| Myocardial infarction | categorical |
| COPD | categorical |
| Diabetes | categorical |
| Latency of HTN (yr) | numerical |
| Total categories of anti-HTN drugs | categorical |
| Hyperthyroidism | categorical |
| PAC (ng/dL), at screening | numerical |
| PRA (ng/mL/hr), at screening | numerical |
| SBP (mmHg) | numerical |
| dBP (mmHg) | numerical |
| Na+ ( mmol/L) | numerical |
| K+ ( mmol/L) | numerical |

### Abbreviations: PAC= plasma aldosterone concentration; PRA=plasma renin activity; SBP=systolic blood pressure; dBP=diastolic blood pressure; BUN=blood urea nitrogen; COPD=chronic obstructive pulmonary disease.

**Supplemental Table 5.** Extracted features used in the random forest model and the detailed prescription

| Features | Notes |
| --- | --- |
| Sex_1 | Male |
| Sex_2 | Female |
| Age (y/o) |  |
| Family history_0 | Neither parent had hypertension |
| Family history_1 | Either parent had hypertension |
| Family history_2 | Both parents had hypertension |
| Body weight (Kg) |  |
| Height (cm) |  |
| Myocardial infarction_0 | without myocardial infarction |
| Myocardial infarction_1 | with myocardial infarction |
| COPD_0 | without COPD |
| COPD_1 | with COPD |
| diabetes_0 | without diabetes |
| diabetes_1 | with diabetes |
| Latency of HTN (yr) |  |
| Categories of anti-HTN drugs |  |
| 0 | No anti-HTN drugs |
| 1 | Category 1 of anti-HTN drugs |
| 2 | Category 2 of anti-HTN drugs |
| 3 | Category 3 of anti-HTN drugs |
| 4 | Category 4 of anti-HTN drugs |
| 5 | Category 5 of anti-HTN drugs |
| 6 | Category 6 of anti-HTN drugs |
| Hyperthyroidism |  |
| No | without hyperthyroidism |
| Yes | with hyperthyroidism |
| PAC (ng/dL), at screening | Plasma aldosterone concentration |
| PRA (ng/mL/hr), at screening | Plasma renin activity |
| SBP (mmHg) | Systolic blood pressure |
| dBP (mmHg) | Diastolic blood pressure |
| Na+ (mmol/L) | Sodium |
| K+ (mmol/L) | Potassium |

### Abbreviations: PAC= plasma aldosterone concentration; PRA=plasma renin activity; SBP=systolic blood pressure; dBP=diastolic blood pressure; BUN=blood urea nitrogen; COPD=chronic obstructive pulmonary disease.

**Supplemental Table 6.** External Validation of South Korean and CONPASS Dataset

|  | **South Korea** | **CONPASS** |
| --- | --- | --- |
| Accuracy | 0.667 (95% CI [0.548, 0.786]) | 0.629 (95% CI [0.592, 0.666]) |
| AUROC | 0.768 (95% CI [0.648, 0.888]) | 0.775 (95% CI [0.739, 0.811]) |

### Abbreviations: AUROC = area under the receiver operating characteristic curve

Figures:


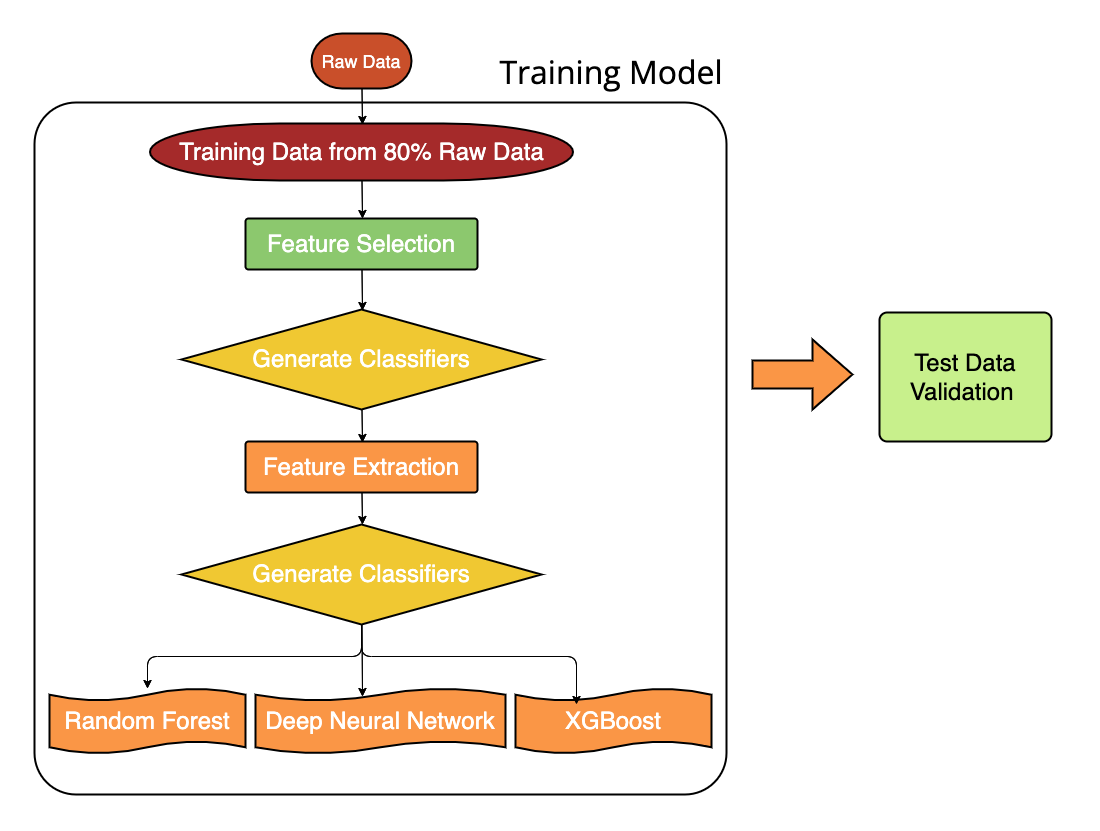


**Supplemental** **Figure 1. Processing Flow Chart**

The data processing flowchart, including developing and validating methods.


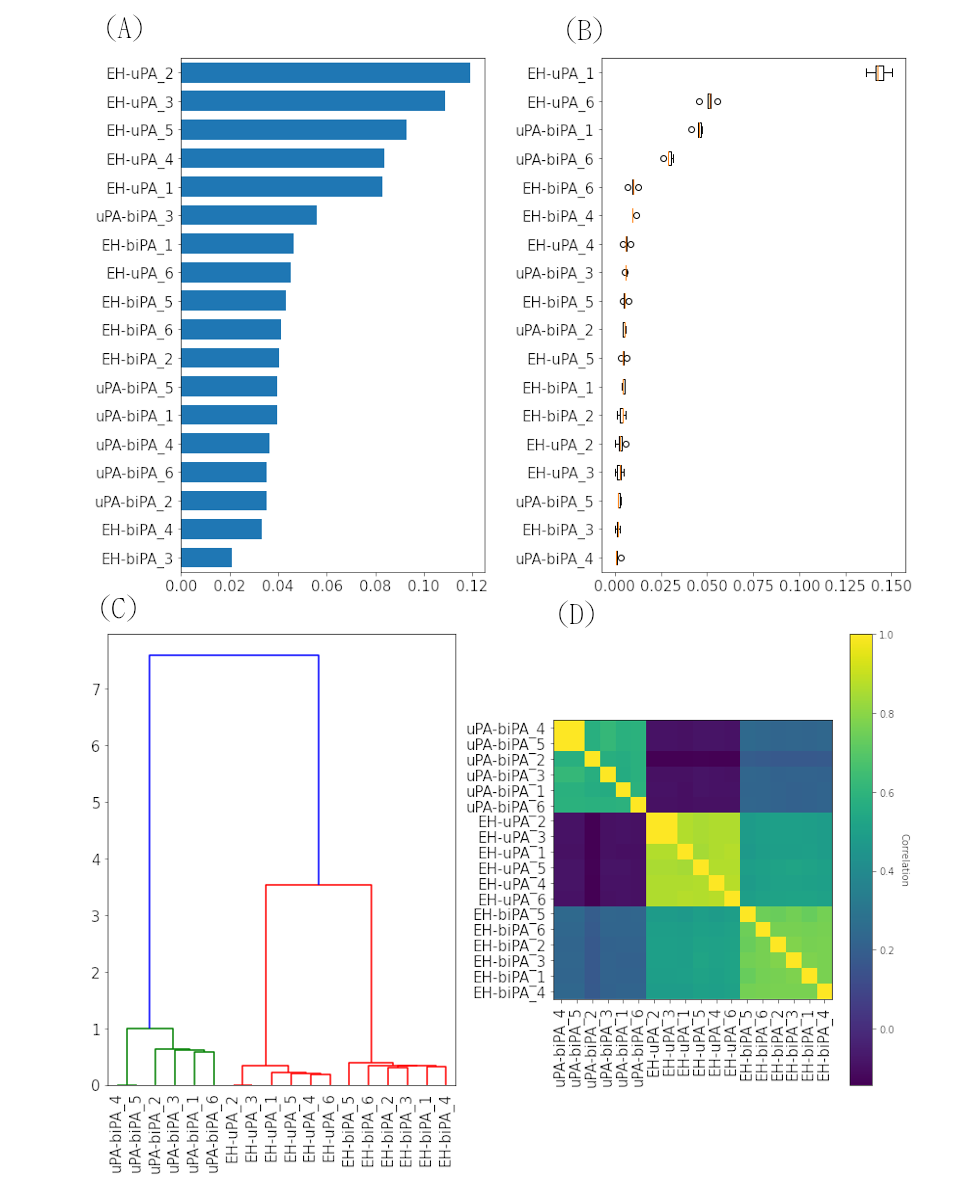


**Supplemental** **Figure 2. Permutation Feature Importance of Random Forest During Feature Selection**

(A) Factors showed feature importance of ML model by dimensionality reduction, that breaks the relationship between the feature and the true outcome after feature extraction. (B) Permutation feature importance was based on TAIPAI dataset, the permutation feature importance considering both the main feature effect and the interaction effects on Random Forest model performance. (C) Cluster dendrogram depicted the correlations between features in hierarchical structure. The correlations were calculated by the permutation feature importance. (D) Heatmap showed the correlation of clinical characteristics. Yellow color depicted positive correlation while blue color depicts negative correlation.

**Abbreviations:** uPA= unilateral primary aldosteronism; biPA= bilateral primary aldosteronism; EH= essential hypertension.


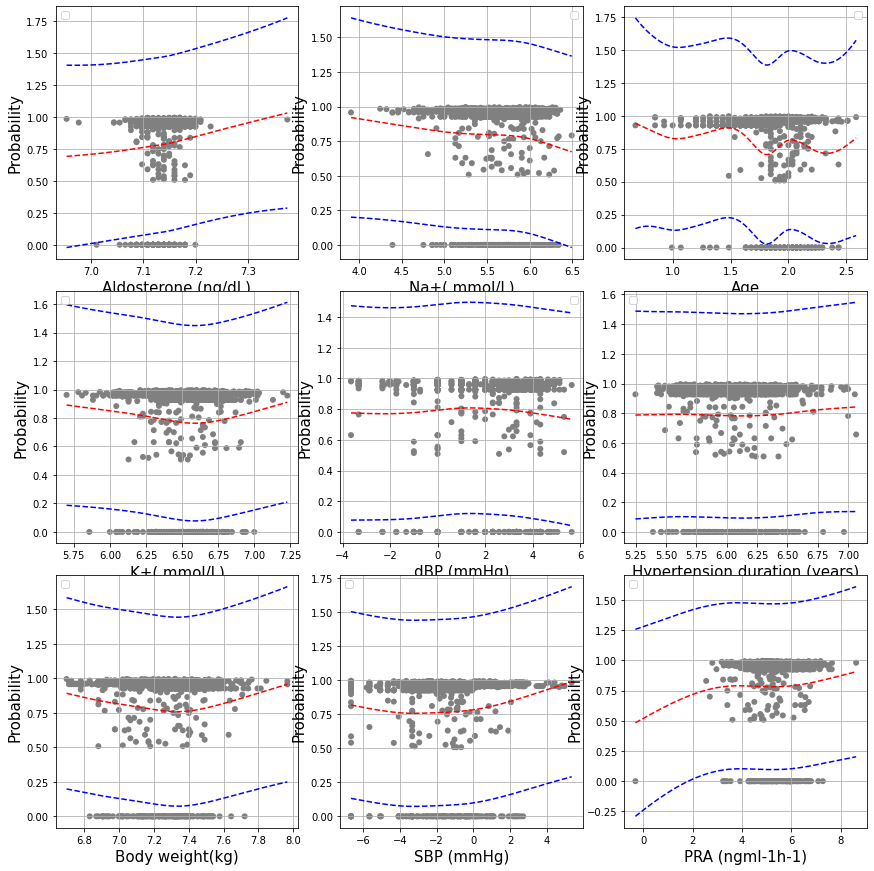


**Supplemental Figure 3.** **GAM Plots of the Most Important Variables of the ML Model**

GAM plots of the most important variables of the ML model depicting the possibility of PA. The x-axis is the logarithm of the values. The y-axis represents the probability of PA. The red lines represent prediction based on the values of the x-axis and the blue lines represent 95% prediction interval. The gray dots are the scatter plots of the original data, which show mostly non-linear relationships between prediction probability and the features. Of note, Aldosterone at screening stage had the highest relationship to PA and the others were non-linear.

### Abbreviations: PA=primary aldosteronism; SBP=systolic blood pressure; dBP=diastolic blood pressure; PRA=plasma renin activity.


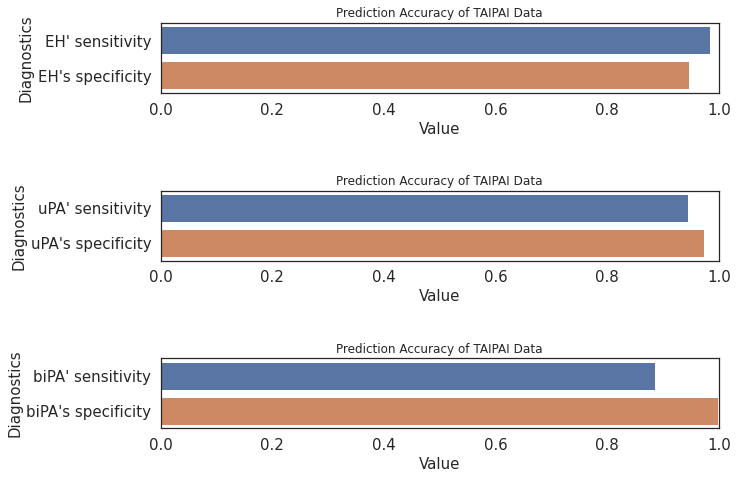


(A) TAIPAI model


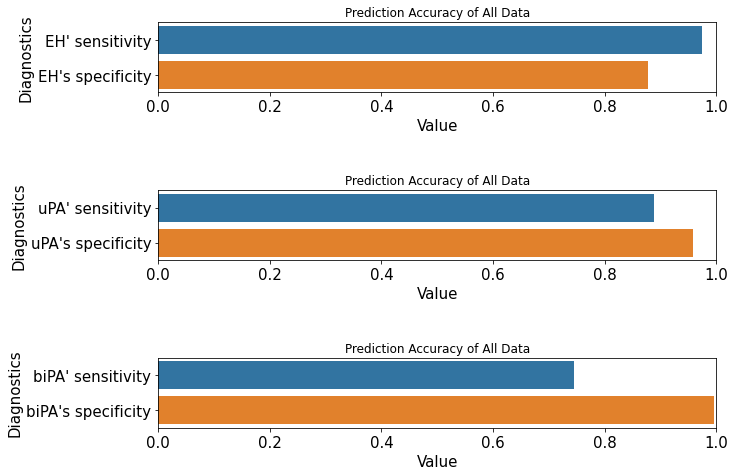


(B) TAIPAI, South Korea, and CONPASS models*

**Supplemental Figure 4**. **Bar graphs showing sensitivity and specificity in consecutive**

Bar graphs showing sensitivity and specificity in consecutive (A)TAIPAI set, (B) Korea and CONPASS participants to identify the diagnosis of EH vs. uPA vs. biPA.

**Note:** *Cross validation of all data.

**Abbreviations:** uPA= unilateral primary aldosteronism; biPA= bilateral primary aldosteronism; EH= essential hypertension.

| **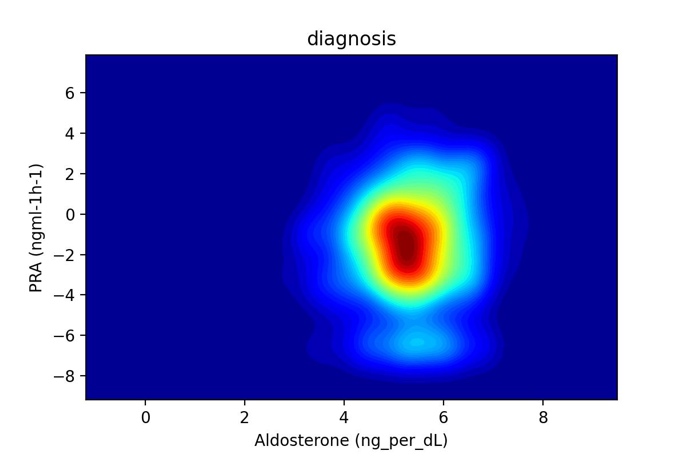(a)** | 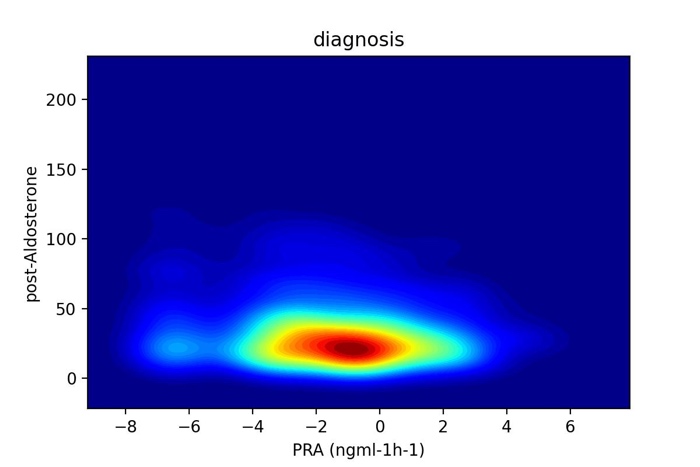  **(b)** |
| --- | --- |

**Supplemental Figure 5. KDE Plots**

The kernel density depicting the diagnosis of PA based on the baseline PRA and PAC on the screening stage. These kernel density estimation (KDE) plots display Aldosterone at screening stage could distinguish EH vs. PA. Both plots demonstrate that the KDE method effectively identifies regions with high concentrations of data points. In plot (a), a concentrated central region is observed, possibly indicating a stronger relationship between Aldosterone and PRA levels. In plot (b), the spread of the high-density region suggests variability in post-Aldosterone levels at certain PRA values, highlighting the value of KDE in capturing non-linear interactions.

In plot (b), the spread of the high-density region suggests variability in post-Aldosterone levels at certain PRA values.

**Abbreviations:** uPA= unilateral primary aldosteronism; biPA= bilateral primary aldosteronism; EH= essential hypertension; PAC**=** plasma aldosterone concentration; PRA**=**plasma renin activity.

**(A)**

**
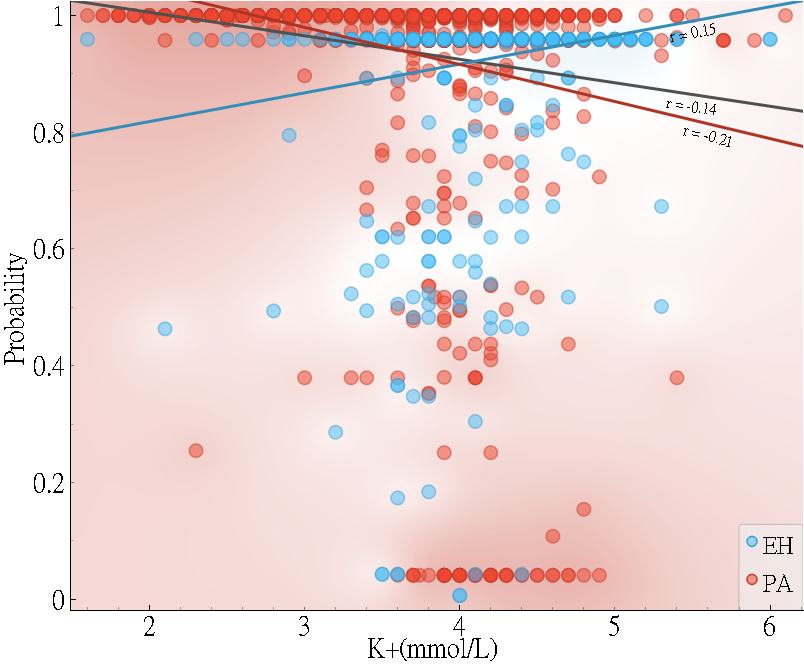
**

**(B)**

**
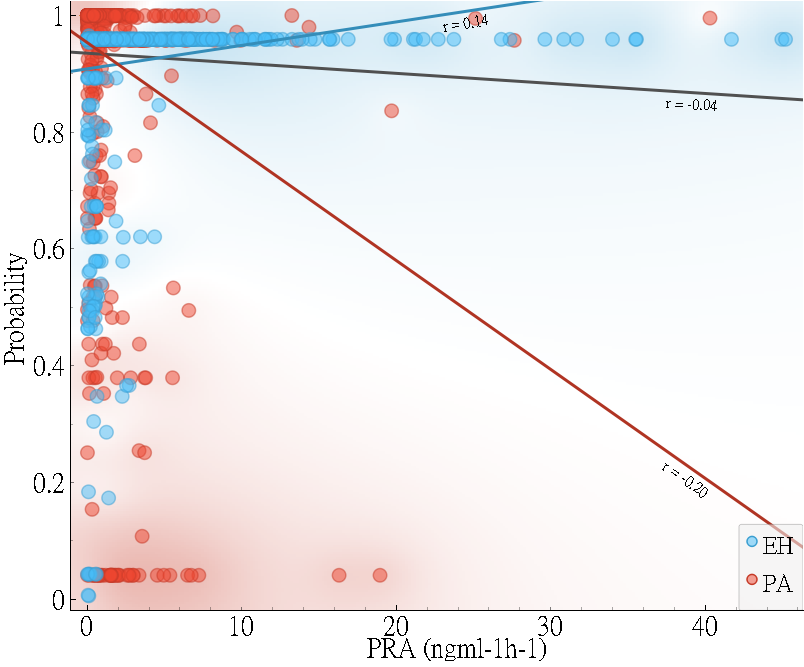
**

**Supplemental Figure 6. Scatter Plot Figures Showing the Probability Distribution and Regression line of EH or PA**

Scatter plot figures with informative projections showing the probability distribution and regression line of essential hypertension (EH) or primary aldosteronism (PA) against (a) serum potassium level and (b) plasma renin activity (PRA). Each data instance in these plots was evaluated using the k-nearest neighbors (kNN) algorithm, which identified the 10 nearest neighbors in the XY space—specifically, the combinations of attribute pairs such as (probability and potassium) or (probability and PRA).

For each data point, kNN determined how many of the nearest neighbors had the same condition (color) as the central point, indicating either PA or EH. The total score for each projection was calculated as the average number of similarly colored neighbors among the 10 nearest neighbors. This score was then used to project the data points into a more informative plot.

The three lines in each figure were constructed using linear least-squares regression. The blue line represents EH, the red line represents PA, and the black line represents the combination of EH and PA. The figures demonstrate that PA had a higher absolute value of the correlation coefficient (r-value) compared to EH, indicating a stronger negative correlation between PA and both PRA and potassium levels.

**Abbreviations:** *Blue line=EH; red line=PA; black line=all participants; PA= primary aldosteronism; EH= essential hypertension; PRA=plasma renin activity.

**Supplemental Figure 7. Decision Curve Analysis**

The image presents a Decision Curve Analysis (DCA) graph, which evaluates the net benefit of different predictive models across a range of threshold probabilities. The x-axis represents the threshold probability—the probability at which a patient would opt for treatment. The y-axis represents the net benefit, a metric that accounts for both true positives and false positives to assess the clinical utility of a model.

The plot includes four lines representing different strategies: ‘None’ (black line) assumes no patients are treated, resulting in a net benefit of zero; ‘All’ (gray line) assumes all patients are treated, establishing a baseline net benefit. The dashed lines represent the net benefit of using serum potassium (K) and plasma renin activity (PRA) as individual predictors, while the green line labeled ‘Prediction’ corresponds to the net benefit of the predictive model that incorporates both K and PRA.

The 'Prediction' model exhibits a higher net benefit across a wide range of threshold probabilities compared to the individual predictors (K and PRA) and the baseline strategies ('None' and 'All'). The curve's shape indicates that the integrated model is more effective at distinguishing between patients who should and should not receive treatment, particularly within the threshold probability range of 0.2 to 0.8. This suggests that the combined model offers a superior balance between detecting true positives and minimizing false positives, making it a more clinically useful tool for decision-making.

**Abbreviations:** PA= primary aldosteronism; PRA=plasma renin activity.

**
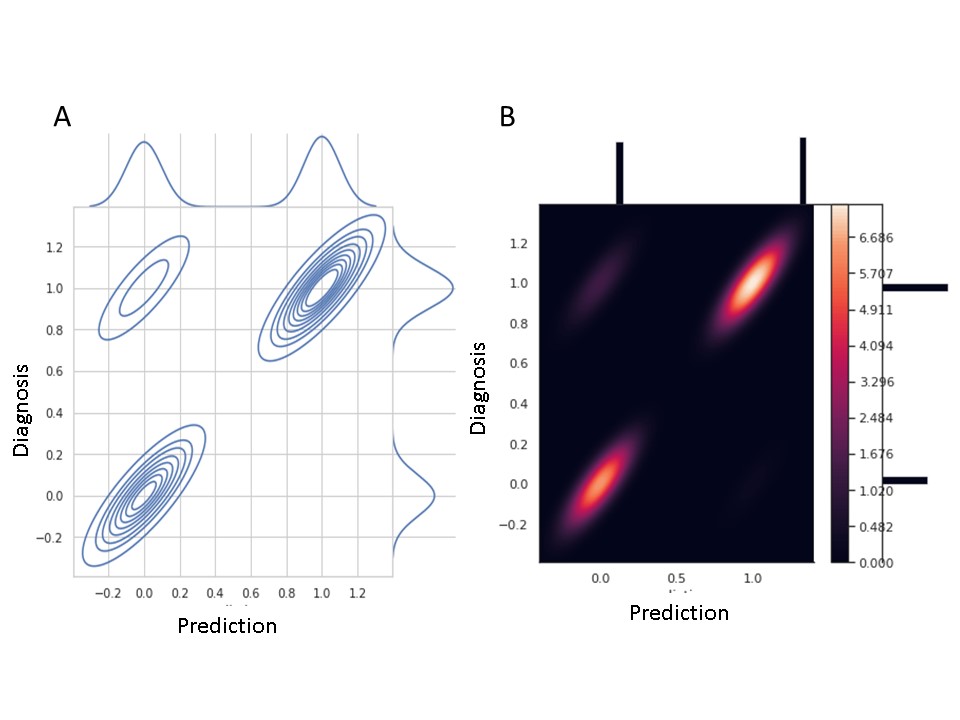
**

**Supplemental Figure 8. 2D KDE Plots**

2D KDE plot depicting the calibration between the prediction and the diagnosis of PA. (A) Bivariate distribution with density plots plotted on both margins; (B) Shaded bivariate distribution with marginal histograms plotted on both the margins, showing prediction overlapping actual diagnosis, which means that the models distinguished EH and PA properly. Bivariate analysis explored the relationship between prediction and actual diagnosis and showed that it was consistent along the diagonal line, with a few pseudo-negative outliers in the upper-left area. The heavy and dense distribution with few outliners represented a close relationship in calibration.

**Abbreviations:** PA= primary aldosteronism; EH= essential hypertension;(A)


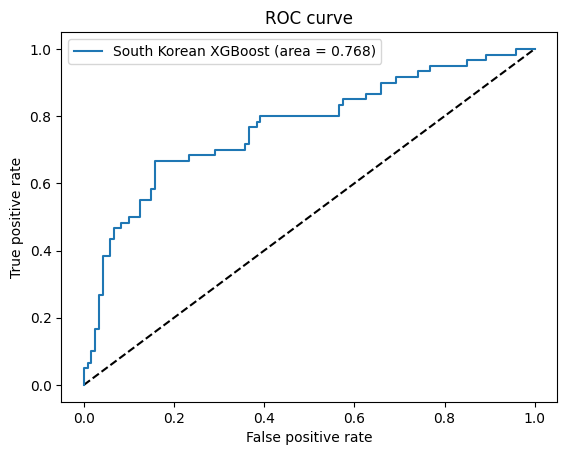


(B)


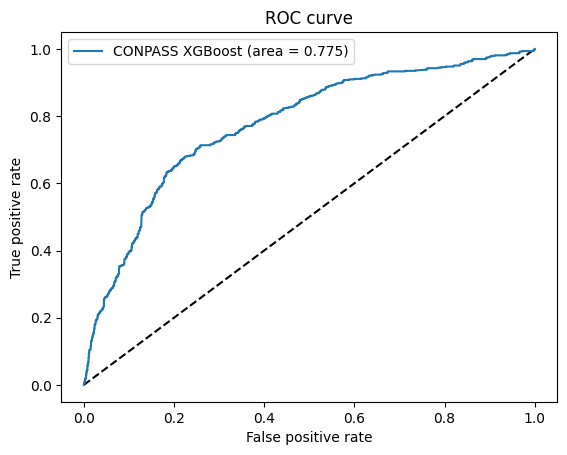


**Supplemental Figure 9. External Validation with South Korean and CONPASS Dataset with XGBoost Model**

We have trained our models with TAIPAI data and validated our XGBoost model with datasets from South Korea and CONPASS. We choose XGBoost as the external validation model, because it performs well in our original design. (A) ROC curve of South Korean dataset and the AUC value is 0.768. (B) ROC Curve of CONPASS dataset and the AUC value is 0.775.

**Abbreviations:** ROC = receiver operating characteristic

**Supplemental Model Development**

**Feature selection and extraction**

We construct a two-layer model with feature selection and feature extraction. In the first layer, we selected several subsets of features for the prediction, according the highest accuracy between EH vs. uPA and EH vs. biPA was 0.82, while the accuracy of uPA and biPA was 0.76 that was below our expectation. Therefore, we further constructed the second layer by way of feature extraction. We use the prediction of the first constructed layer as a new feature to do the second selection.

1. We generate subsets and choose subsets with high accuracy from all features then decreasing features one by one to speed up the feature selection process.
2. We use random forest to perform feature extraction. Random forest is a method by which several decision trees are built from the variable set.
3. We constructed random forest classifiers to select important feature subsets, and extract features from the prediction based on feature subsets.
4. Before we use feature extraction by random forest, we observe the accuracies of random forest in 100 feature selections in uPA vs. biPA, EH vs. uPA, and EH vs. biPA separately.
5. We find the higher accuracies of EH vs. biPA is 0.75, EH vs. uPA is 0.75, and uPA vs. biPA is 0.66. We choose feature subsets if their accuracies are higher the previous one.

**Algorithm**

*
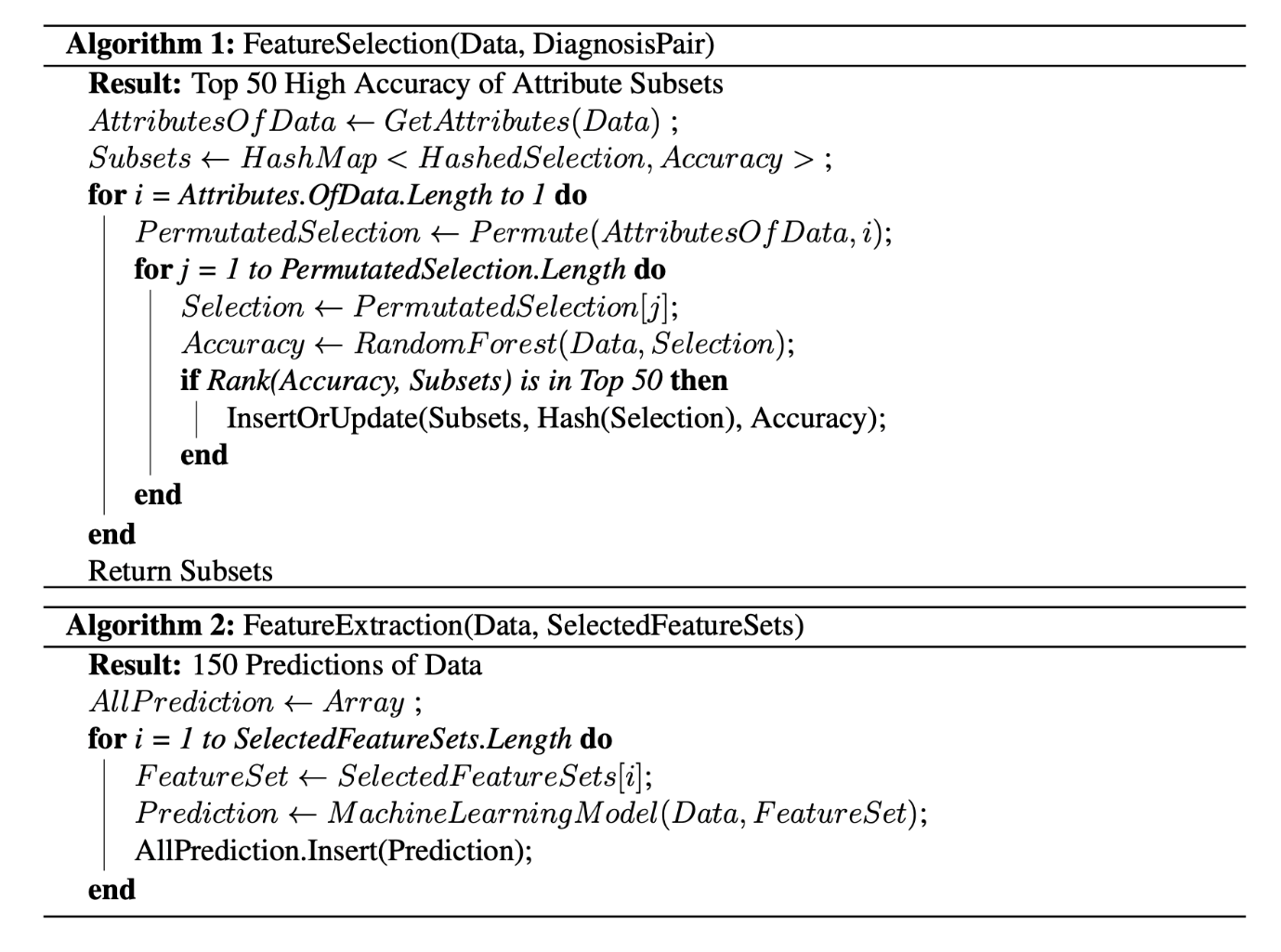
*

**Scoring Function**

We considered two methods to determine the cut-off point for distinguishing between EH, uPA, and biPA patients. The first method was based on the Youden index to determine the point at which the summation of sensitivity and specificity is maximal, and the second method determined the point that yields the highest sensitivity with a minimum specificity of 0.7; here, we designate the former as the regular threshold and the latter as the special threshold. With these thresholds, sensitivity, specificity, positive predictive value (PPV), and negative predictive value (NPV) were calculated to analyze the diagnostic ability of the proposed scoring function.

**Feature selection**

The feature of “EH-uPA_1” means the value extracted from the first classifier with original features listed in Figure2 for EH vs. uPA. The feature of “EH-uPA_3” means the value from the third classifier for EH vs. uPA. The feature of “EH-biPA_2” means the value from the second classifier for EH vs. biPA, and “uPA-biPA_2” means the

value from the second classifier for uPA vs. biPA.

Factor_1:

['age', 'Body weight(kg)', 'height (cm)', 'hypertension duration (years)', 'Aldosterone (ng/dL)', 'PRA (ngml-1h-1)', 'SBP (mmHg)', 'dBP (mmHg)', 'Na+(\xa0mmol/L)', 'K+(\xa0mmol/L)', 'ever_Hyperthyroidism_0', 'ever_Hyperthyroidism_1', 'ever_COPD_0', 'ever_COPD_1', 'ever diabetics_0', 'ever diabetics_1', 'ever_myocardiac infarction_0', 'ever_myocardiac infarction_1', 'Sex_1', 'Sex_2', 'family history_0', 'family history_1', 'family history_2', 'total categories of antiHTN drugs_0', 'total categories of antiHTN drugs_1', 'total categories of antiHTN drugs_2', 'total categories of antiHTN drugs_3', 'total categories of antiHTN drugs_4', 'total categories of antiHTN drugs_5']

Factor_2:

['age', 'Body weight(kg)', 'height (cm)', 'hypertension duration (years)', 'Aldosterone (ng/dL)', 'PRA (ngml-1h-1)', 'SBP (mmHg)', 'dBP (mmHg)', 'Na+(\xa0mmol/L)', 'K+(\xa0mmol/L)', 'ever_Hyperthyroidism_0', 'ever_Hyperthyroidism_1', 'ever_COPD_0', 'ever_COPD_1', 'ever diabetics_0', 'ever diabetics_1', 'ever_myocardiac infarction_0', 'ever_myocardiac infarction_1', 'Sex_1', 'Sex_2', 'family history_0', 'family history_1', 'family history_2', 'total categories of antiHTN drugs_0', 'total categories of antiHTN drugs_1', 'total categories of antiHTN drugs_2', 'total categories of antiHTN drugs_3', 'total categories of antiHTN drugs_4']

Factor_3:

['age', 'Body weight(kg)', 'height (cm)', 'hypertension duration (years)', 'Aldosterone (ng/dL)', 'PRA (ngml-1h-1)', 'SBP (mmHg)', 'dBP (mmHg)', 'Na+(\xa0mmol/L)', 'K+(\xa0mmol/L)', 'ever_Hyperthyroidism_0', 'ever_Hyperthyroidism_1', 'ever_COPD_0', 'ever_COPD_1', 'ever diabetics_0', 'ever diabetics_1', 'ever_myocardiac infarction_0', 'ever_myocardiac infarction_1', 'Sex_1', 'Sex_2', 'family history_0', 'family history_1', 'family history_2', 'total categories of antiHTN drugs_0', 'total categories of antiHTN drugs_1', 'total categories of antiHTN drugs_2', 'total categories of antiHTN drugs_3', 'total categories of antiHTN drugs_5']

Factor_4:

['age', 'Body weight(kg)', 'height (cm)', 'hypertension duration (years)', 'Aldosterone (ng/dL)', 'PRA (ngml-1h-1)', 'SBP (mmHg)', 'dBP (mmHg)', 'Na+(\xa0mmol/L)', 'K+(\xa0mmol/L)', 'ever_Hyperthyroidism_0', 'ever_Hyperthyroidism_1', 'ever_COPD_0', 'ever_COPD_1', 'ever diabetics_0', 'ever diabetics_1', 'ever_myocardiac infarction_0', 'ever_myocardiac infarction_1', 'Sex_1', 'Sex_2', 'family history_0', 'family history_1', 'family history_2', 'total categories of antiHTN drugs_0', 'total categories of antiHTN drugs_1', 'total categories of antiHTN drugs_2', 'total categories of antiHTN drugs_4', 'total categories of antiHTN drugs_5']

Factor_5:

['age', 'Body weight(kg)', 'height (cm)', 'hypertension duration (years)', 'Aldosterone (ng/dL)', 'PRA (ngml-1h-1)', 'SBP (mmHg)', 'dBP (mmHg)', 'Na+(\xa0mmol/L)', 'K+(\xa0mmol/L)', 'ever_Hyperthyroidism_0', 'ever_Hyperthyroidism_1', 'ever_COPD_0', 'ever_COPD_1', 'ever diabetics_0', 'ever diabetics_1', 'ever_myocardiac infarction_0', 'ever_myocardiac infarction_1', 'Sex_1', 'Sex_2', 'family history_0', 'family history_1', 'family history_2', 'total categories of antiHTN drugs_0', 'total categories of antiHTN drugs_1', 'total categories of antiHTN drugs_3', 'total categories of antiHTN drugs_4', 'total categories of antiHTN drugs_5']

Factor_6:

['age', 'Body weight(kg)', 'height (cm)', 'hypertension duration (years)', 'Aldosterone (ng/dL)', 'PRA (ngml-1h-1)', 'SBP (mmHg)', 'dBP (mmHg)', 'Na+(\xa0mmol/L)', 'K+(\xa0mmol/L)', 'ever_Hyperthyroidism_0', 'ever_Hyperthyroidism_1', 'ever_COPD_0', 'ever_COPD_1', 'ever diabetics_0', 'ever diabetics_1', 'ever_myocardiac infarction_0', 'ever_myocardiac infarction_1', 'Sex_1', 'Sex_2', 'family history_0', 'family history_1', 'family history_2', 'total categories of antiHTN drugs_0', 'total categories of antiHTN drugs_2', 'total categories of antiHTN drugs_3', 'total categories of antiHTN drugs_4', 'total categories of antiHTN drugs_5']

Factor_7:

['age', 'Body weight(kg)', 'height (cm)', 'hypertension duration (years)', 'Aldosterone (ng/dL)', 'PRA (ngml-1h-1)', 'SBP (mmHg)', 'dBP (mmHg)', 'Na+(\xa0mmol/L)', 'K+(\xa0mmol/L)', 'ever_Hyperthyroidism_0', 'ever_Hyperthyroidism_1', 'ever_COPD_0', 'ever_COPD_1', 'ever diabetics_0', 'ever diabetics_1', 'ever_myocardiac infarction_0', 'ever_myocardiac infarction_1', 'Sex_1', 'Sex_2', 'family history_0', 'family history_1', 'family history_2', 'total categories of antiHTN drugs_1', 'total categories of antiHTN drugs_2', 'total categories of antiHTN drugs_3', 'total categories of antiHTN drugs_4', 'total categories of antiHTN drugs_5']

Factor_8:

['age', 'Body weight(kg)', 'height (cm)', 'hypertension duration (years)', 'Aldosterone (ng/dL)', 'PRA (ngml-1h-1)', 'SBP (mmHg)', 'dBP (mmHg)', 'Na+(\xa0mmol/L)', 'K+(\xa0mmol/L)', 'ever_Hyperthyroidism_0', 'ever_Hyperthyroidism_1', 'ever_COPD_0', 'ever_COPD_1', 'ever diabetics_0', 'ever diabetics_1', 'ever_myocardiac infarction_0', 'ever_myocardiac infarction_1', 'Sex_1', 'Sex_2', 'family history_0', 'family history_1', 'total categories of antiHTN drugs_0', 'total categories of antiHTN drugs_1', 'total categories of antiHTN drugs_2', 'total categories of antiHTN drugs_3', 'total categories of antiHTN drugs_4', 'total categories of antiHTN drugs_5']

Factor_9:

['age', 'Body weight(kg)', 'height (cm)', 'hypertension duration (years)', 'Aldosterone (ng/dL)', 'PRA (ngml-1h-1)', 'SBP (mmHg)', 'dBP (mmHg)', 'Na+(\xa0mmol/L)', 'K+(\xa0mmol/L)', 'ever_Hyperthyroidism_0', 'ever_Hyperthyroidism_1', 'ever_COPD_0', 'ever_COPD_1', 'ever diabetics_0', 'ever diabetics_1', 'ever_myocardiac infarction_0', 'ever_myocardiac infarction_1', 'Sex_1', 'Sex_2', 'family history_0', 'family history_2', 'total categories of antiHTN drugs_0', 'total categories of antiHTN drugs_1', 'total categories of antiHTN drugs_2', 'total categories of antiHTN drugs_3', 'total categories of antiHTN drugs_4', 'total categories of antiHTN drugs_5']

Factor_10:

['age', 'Body weight(kg)', 'height (cm)', 'hypertension duration (years)', 'Aldosterone (ng/dL)', 'PRA (ngml-1h-1)', 'SBP (mmHg)', 'dBP (mmHg)', 'Na+(\xa0mmol/L)', 'K+(\xa0mmol/L)', 'ever_Hyperthyroidism_0', 'ever_Hyperthyroidism_1', 'ever_COPD_0', 'ever_COPD_1', 'ever diabetics_0', 'ever diabetics_1', 'ever_myocardiac infarction_0', 'ever_myocardiac infarction_1', 'Sex_1', 'Sex_2', 'family history_1', 'family history_2', 'total categories of antiHTN drugs_0', 'total categories of antiHTN drugs_1', 'total categories of antiHTN drugs_2', 'total categories of antiHTN drugs_3', 'total categories of antiHTN drugs_4', 'total categories of antiHTN drugs_5']

Factor_11:

['age', 'Body weight(kg)', 'height (cm)', 'hypertension duration (years)', 'Aldosterone (ng/dL)', 'PRA (ngml-1h-1)', 'SBP (mmHg)', 'dBP (mmHg)', 'Na+(\xa0mmol/L)', 'K+(\xa0mmol/L)', 'ever_Hyperthyroidism_0', 'ever_Hyperthyroidism_1', 'ever_COPD_0', 'ever_COPD_1', 'ever diabetics_0', 'ever diabetics_1', 'ever_myocardiac infarction_0', 'ever_myocardiac infarction_1', 'Sex_1', 'family history_0', 'family history_1', 'family history_2', 'total categories of antiHTN drugs_0', 'total categories of antiHTN drugs_1', 'total categories of antiHTN drugs_2', 'total categories of antiHTN drugs_3', 'total categories of antiHTN drugs_4', 'total categories of antiHTN drugs_5']

Factor_12:

['age', 'Body weight(kg)', 'height (cm)', 'hypertension duration (years)', 'Aldosterone (ng/dL)', 'PRA (ngml-1h-1)', 'SBP (mmHg)', 'dBP (mmHg)', 'Na+(\xa0mmol/L)', 'K+(\xa0mmol/L)', 'ever_Hyperthyroidism_0', 'ever_Hyperthyroidism_1', 'ever_COPD_0', 'ever_COPD_1', 'ever diabetics_0', 'ever diabetics_1', 'ever_myocardiac infarction_0', 'ever_myocardiac infarction_1', 'Sex_2', 'family history_0', 'family history_1', 'family history_2', 'total categories of antiHTN drugs_0', 'total categories of antiHTN drugs_1', 'total categories of antiHTN drugs_2', 'total categories of antiHTN drugs_3', 'total categories of antiHTN drugs_4', 'total categories of antiHTN drugs_5']

Factor_13:

['age', 'Body weight(kg)', 'height (cm)', 'hypertension duration (years)', 'Aldosterone (ng/dL)', 'PRA (ngml-1h-1)', 'SBP (mmHg)', 'dBP (mmHg)', 'Na+(\xa0mmol/L)', 'K+(\xa0mmol/L)', 'ever_Hyperthyroidism_0', 'ever_Hyperthyroidism_1', 'ever_COPD_0', 'ever_COPD_1', 'ever diabetics_0', 'ever diabetics_1', 'ever_myocardiac infarction_0', 'Sex_1', 'Sex_2', 'family history_0', 'family history_1', 'family history_2', 'total categories of antiHTN drugs_0', 'total categories of antiHTN drugs_1', 'total categories of antiHTN drugs_2', 'total categories of antiHTN drugs_3', 'total categories of antiHTN drugs_4', 'total categories of antiHTN drugs_5']

Factor_14:

['age', 'Body weight(kg)', 'height (cm)', 'hypertension duration (years)', 'Aldosterone (ng/dL)', 'PRA (ngml-1h-1)', 'SBP (mmHg)', 'dBP (mmHg)', 'Na+(\xa0mmol/L)', 'K+(\xa0mmol/L)', 'ever_Hyperthyroidism_0', 'ever_Hyperthyroidism_1', 'ever_COPD_0', 'ever_COPD_1', 'ever diabetics_0', 'ever diabetics_1', 'ever_myocardiac infarction_1', 'Sex_1', 'Sex_2', 'family history_0', 'family history_1', 'family history_2', 'total categories of antiHTN drugs_0', 'total categories of antiHTN drugs_1', 'total categories of antiHTN drugs_2', 'total categories of antiHTN drugs_3', 'total categories of antiHTN drugs_4', 'total categories of antiHTN drugs_5']

Factor_15:

['age', 'Body weight(kg)', 'height (cm)', 'hypertension duration (years)', 'Aldosterone (ng/dL)', 'PRA (ngml-1h-1)', 'SBP (mmHg)', 'dBP (mmHg)', 'Na+(\xa0mmol/L)', 'K+(\xa0mmol/L)', 'ever_Hyperthyroidism_0', 'ever_Hyperthyroidism_1', 'ever_COPD_0', 'ever_COPD_1', 'ever diabetics_0', 'ever_myocardiac infarction_0', 'ever_myocardiac infarction_1', 'Sex_1', 'Sex_2', 'family history_0', 'family history_1', 'family history_2', 'total categories of antiHTN drugs_0', 'total categories of antiHTN drugs_1', 'total categories of antiHTN drugs_2', 'total categories of antiHTN drugs_3', 'total categories of antiHTN drugs_4', 'total categories of antiHTN drugs_5']

Factor_16:

['age', 'Body weight(kg)', 'height (cm)', 'hypertension duration (years)', 'Aldosterone (ng/dL)', 'PRA (ngml-1h-1)', 'SBP (mmHg)', 'dBP (mmHg)', 'Na+(\xa0mmol/L)', 'K+(\xa0mmol/L)', 'ever_Hyperthyroidism_0', 'ever_Hyperthyroidism_1', 'ever_COPD_0', 'ever_COPD_1', 'ever diabetics_1', 'ever_myocardiac infarction_0', 'ever_myocardiac infarction_1', 'Sex_1', 'Sex_2', 'family history_0', 'family history_1', 'family history_2', 'total categories of antiHTN drugs_0', 'total categories of antiHTN drugs_1', 'total categories of antiHTN drugs_2', 'total categories of antiHTN drugs_3', 'total categories of antiHTN drugs_4', 'total categories of antiHTN drugs_5']

Factor_17:

['age', 'Body weight(kg)', 'height (cm)', 'hypertension duration (years)', 'Aldosterone (ng/dL)', 'PRA (ngml-1h-1)', 'SBP (mmHg)', 'dBP (mmHg)', 'Na+(\xa0mmol/L)', 'K+(\xa0mmol/L)', 'ever_Hyperthyroidism_0', 'ever_Hyperthyroidism_1', 'ever_COPD_0', 'ever diabetics_0', 'ever diabetics_1', 'ever_myocardiac infarction_0', 'ever_myocardiac infarction_1', 'Sex_1', 'Sex_2', 'family history_0', 'family history_1', 'family history_2', 'total categories of antiHTN drugs_0', 'total categories of antiHTN drugs_1', 'total categories of antiHTN drugs_2', 'total categories of antiHTN drugs_3', 'total categories of antiHTN drugs_4', 'total categories of antiHTN drugs_5']

Factor_18:

['age', 'Body weight(kg)', 'height (cm)', 'hypertension duration (years)', 'Aldosterone (ng/dL)', 'PRA (ngml-1h-1)', 'SBP (mmHg)', 'dBP (mmHg)', 'Na+(\xa0mmol/L)', 'K+(\xa0mmol/L)', 'ever_Hyperthyroidism_0', 'ever_Hyperthyroidism_1', 'ever_COPD_1', 'ever diabetics_0', 'ever diabetics_1', 'ever_myocardiac infarction_0', 'ever_myocardiac infarction_1', 'Sex_1', 'Sex_2', 'family history_0', 'family history_1', 'family history_2', 'total categories of antiHTN drugs_0', 'total categories of antiHTN drugs_1', 'total categories of antiHTN drugs_2', 'total categories of antiHTN drugs_3', 'total categories of antiHTN drugs_4', 'total categories of antiHTN drugs_5']

Factor_19:

['age', 'Body weight(kg)', 'height (cm)', 'hypertension duration (years)', 'Aldosterone (ng/dL)', 'PRA (ngml-1h-1)', 'SBP (mmHg)', 'dBP (mmHg)', 'Na+(\xa0mmol/L)', 'K+(\xa0mmol/L)', 'ever_Hyperthyroidism_0', 'ever_COPD_0', 'ever_COPD_1', 'ever diabetics_0', 'ever diabetics_1', 'ever_myocardiac infarction_0', 'ever_myocardiac infarction_1', 'Sex_1', 'Sex_2', 'family history_0', 'family history_1', 'family history_2', 'total categories of antiHTN drugs_0', 'total categories of antiHTN drugs_1', 'total categories of antiHTN drugs_2', 'total categories of antiHTN drugs_3', 'total categories of antiHTN drugs_4', 'total categories of antiHTN drugs_5']

Factor_20:

['age', 'Body weight(kg)', 'height (cm)', 'hypertension duration (years)', 'Aldosterone (ng/dL)', 'PRA (ngml-1h-1)', 'SBP (mmHg)', 'dBP (mmHg)', 'Na+(\xa0mmol/L)', 'K+(\xa0mmol/L)', 'ever_Hyperthyroidism_1', 'ever_COPD_0', 'ever_COPD_1', 'ever diabetics_0', 'ever diabetics_1', 'ever_myocardiac infarction_0', 'ever_myocardiac infarction_1', 'Sex_1', 'Sex_2', 'family history_0', 'family history_1', 'family history_2', 'total categories of antiHTN drugs_0', 'total categories of antiHTN drugs_1', 'total categories of antiHTN drugs_2', 'total categories of antiHTN drugs_3', 'total categories of antiHTN drugs_4', 'total categories of antiHTN drugs_5']

Factor_21:

['age', 'Body weight(kg)', 'height (cm)', 'hypertension duration (years)', 'Aldosterone (ng/dL)', 'PRA (ngml-1h-1)', 'SBP (mmHg)', 'dBP (mmHg)', 'Na+(\xa0mmol/L)', 'ever_Hyperthyroidism_0', 'ever_Hyperthyroidism_1', 'ever_COPD_0', 'ever_COPD_1', 'ever diabetics_0', 'ever diabetics_1', 'ever_myocardiac infarction_0', 'ever_myocardiac infarction_1', 'Sex_1', 'Sex_2', 'family history_0', 'family history_1', 'family history_2', 'total categories of antiHTN drugs_0', 'total categories of antiHTN drugs_1', 'total categories of antiHTN drugs_2', 'total categories of antiHTN drugs_3', 'total categories of antiHTN drugs_4', 'total categories of antiHTN drugs_5']

Factor_22:

['age', 'Body weight(kg)', 'height (cm)', 'hypertension duration (years)', 'Aldosterone (ng/dL)', 'PRA (ngml-1h-1)', 'SBP (mmHg)', 'dBP (mmHg)', 'K+(\xa0mmol/L)', 'ever_Hyperthyroidism_0', 'ever_Hyperthyroidism_1', 'ever_COPD_0', 'ever_COPD_1', 'ever diabetics_0', 'ever diabetics_1', 'ever_myocardiac infarction_0', 'ever_myocardiac infarction_1', 'Sex_1', 'Sex_2', 'family history_0', 'family history_1', 'family history_2', 'total categories of antiHTN drugs_0', 'total categories of antiHTN drugs_1', 'total categories of antiHTN drugs_2', 'total categories of antiHTN drugs_3', 'total categories of antiHTN drugs_4', 'total categories of antiHTN drugs_5']

Factor_23:

['age', 'Body weight(kg)', 'height (cm)', 'hypertension duration (years)', 'Aldosterone (ng/dL)', 'PRA (ngml-1h-1)', 'SBP (mmHg)', 'Na+(\xa0mmol/L)', 'K+(\xa0mmol/L)', 'ever_Hyperthyroidism_0', 'ever_Hyperthyroidism_1', 'ever_COPD_0', 'ever_COPD_1', 'ever diabetics_0', 'ever diabetics_1', 'ever_myocardiac infarction_0', 'ever_myocardiac infarction_1', 'Sex_1', 'Sex_2', 'family history_0', 'family history_1', 'family history_2', 'total categories of antiHTN drugs_0', 'total categories of antiHTN drugs_1', 'total categories of antiHTN drugs_2', 'total categories of antiHTN drugs_3', 'total categories of antiHTN drugs_4', 'total categories of antiHTN drugs_5']

Factor_24:

['age', 'Body weight(kg)', 'height (cm)', 'hypertension duration (years)', 'Aldosterone (ng/dL)', 'PRA (ngml-1h-1)', 'dBP (mmHg)', 'Na+(\xa0mmol/L)', 'K+(\xa0mmol/L)', 'ever_Hyperthyroidism_0', 'ever_Hyperthyroidism_1', 'ever_COPD_0', 'ever_COPD_1', 'ever diabetics_0', 'ever diabetics_1', 'ever_myocardiac infarction_0', 'ever_myocardiac infarction_1', 'Sex_1', 'Sex_2', 'family history_0', 'family history_1', 'family history_2', 'total categories of antiHTN drugs_0', 'total categories of antiHTN drugs_1', 'total categories of antiHTN drugs_2', 'total categories of antiHTN drugs_3', 'total categories of antiHTN drugs_4', 'total categories of antiHTN drugs_5']

Factor_25:

['age', 'Body weight(kg)', 'height (cm)', 'hypertension duration (years)', 'PRA (ngml-1h-1)', 'SBP (mmHg)', 'dBP (mmHg)', 'Na+(\xa0mmol/L)', 'K+(\xa0mmol/L)', 'ever_Hyperthyroidism_0', 'ever_Hyperthyroidism_1', 'ever_COPD_0', 'ever_COPD_1', 'ever diabetics_0', 'ever diabetics_1', 'ever_myocardiac infarction_0', 'ever_myocardiac infarction_1', 'Sex_1', 'Sex_2', 'family history_0', 'family history_1', 'family history_2', 'total categories of antiHTN drugs_0', 'total categories of antiHTN drugs_1', 'total categories of antiHTN drugs_2', 'total categories of antiHTN drugs_3', 'total categories of antiHTN drugs_4', 'total categories of antiHTN drugs_5']

Factor_26:

['age', 'Body weight(kg)', 'height (cm)', 'Aldosterone (ng/dL)', 'PRA (ngml-1h-1)', 'SBP (mmHg)', 'dBP (mmHg)', 'Na+(\xa0mmol/L)', 'K+(\xa0mmol/L)', 'ever_Hyperthyroidism_0', 'ever_Hyperthyroidism_1', 'ever_COPD_0', 'ever_COPD_1', 'ever diabetics_0', 'ever diabetics_1', 'ever_myocardiac infarction_0', 'ever_myocardiac infarction_1', 'Sex_1', 'Sex_2', 'family history_0', 'family history_1', 'family history_2', 'total categories of antiHTN drugs_0', 'total categories of antiHTN drugs_1', 'total categories of antiHTN drugs_2', 'total categories of antiHTN drugs_3', 'total categories of antiHTN drugs_4', 'total categories of antiHTN drugs_5']

Factor_27:

['age', 'Body weight(kg)', 'hypertension duration (years)', 'Aldosterone (ng/dL)', 'PRA (ngml-1h-1)', 'SBP (mmHg)', 'dBP (mmHg)', 'Na+(\xa0mmol/L)', 'K+(\xa0mmol/L)', 'ever_Hyperthyroidism_0', 'ever_Hyperthyroidism_1', 'ever_COPD_0', 'ever_COPD_1', 'ever diabetics_0', 'ever diabetics_1', 'ever_myocardiac infarction_0', 'ever_myocardiac infarction_1', 'Sex_1', 'Sex_2', 'family history_0', 'family history_1', 'family history_2', 'total categories of antiHTN drugs_0', 'total categories of antiHTN drugs_1', 'total categories of antiHTN drugs_2', 'total categories of antiHTN drugs_3', 'total categories of antiHTN drugs_4', 'total categories of antiHTN drugs_5']

Factor_28:

['age', 'height (cm)', 'hypertension duration (years)', 'Aldosterone (ng/dL)', 'PRA (ngml-1h-1)', 'SBP (mmHg)', 'dBP (mmHg)', 'Na+(\xa0mmol/L)', 'K+(\xa0mmol/L)', 'ever_Hyperthyroidism_0', 'ever_Hyperthyroidism_1', 'ever_COPD_0', 'ever_COPD_1', 'ever diabetics_0', 'ever diabetics_1', 'ever_myocardiac infarction_0', 'ever_myocardiac infarction_1', 'Sex_1', 'Sex_2', 'family history_0', 'family history_1', 'family history_2', 'total categories of antiHTN drugs_0', 'total categories of antiHTN drugs_1', 'total categories of antiHTN drugs_2', 'total categories of antiHTN drugs_3', 'total categories of antiHTN drugs_4', 'total categories of antiHTN drugs_5']

Factor_29:

['Body weight(kg)', 'height (cm)', 'hypertension duration (years)', 'Aldosterone (ng/dL)', 'PRA (ngml-1h-1)', 'SBP (mmHg)', 'dBP (mmHg)', 'Na+(\xa0mmol/L)', 'K+(\xa0mmol/L)', 'ever_Hyperthyroidism_0', 'ever_Hyperthyroidism_1', 'ever_COPD_0', 'ever_COPD_1', 'ever diabetics_0', 'ever diabetics_1', 'ever_myocardiac infarction_0', 'ever_myocardiac infarction_1', 'Sex_1', 'Sex_2', 'family history_0', 'family history_1', 'family history_2', 'total categories of antiHTN drugs_0', 'total categories of antiHTN drugs_1', 'total categories of antiHTN drugs_2', 'total categories of antiHTN drugs_3', 'total categories of antiHTN drugs_4', 'total categories of antiHTN drugs_5']

Factor_30:

['age', 'Body weight(kg)', 'height (cm)', 'hypertension duration (years)', 'Aldosterone (ng/dL)', 'PRA (ngml-1h-1)', 'SBP (mmHg)', 'dBP (mmHg)', 'Na+(\xa0mmol/L)', 'K+(\xa0mmol/L)', 'ever_Hyperthyroidism_0', 'ever_Hyperthyroidism_1', 'ever_COPD_0', 'ever_COPD_1', 'ever diabetics_0', 'ever diabetics_1', 'ever_myocardiac infarction_0', 'ever_myocardiac infarction_1', 'Sex_1', 'Sex_2', 'family history_0', 'family history_1', 'family history_2', 'total categories of antiHTN drugs_0', 'total categories of antiHTN drugs_1', 'total categories of antiHTN drugs_2', 'total categories of antiHTN drugs_3']

Factor_31:

['age', 'Body weight(kg)', 'height (cm)', 'hypertension duration (years)', 'Aldosterone (ng/dL)', 'PRA (ngml-1h-1)', 'SBP (mmHg)', 'dBP (mmHg)', 'Na+(\xa0mmol/L)', 'K+(\xa0mmol/L)', 'ever_Hyperthyroidism_0', 'ever_Hyperthyroidism_1', 'ever_COPD_0', 'ever_COPD_1', 'ever diabetics_0', 'ever diabetics_1', 'ever_myocardiac infarction_0', 'ever_myocardiac infarction_1', 'Sex_1', 'Sex_2', 'family history_0', 'family history_1', 'family history_2', 'total categories of antiHTN drugs_0', 'total categories of antiHTN drugs_1', 'total categories of antiHTN drugs_2', 'total categories of antiHTN drugs_4']

Factor_32:

['age', 'Body weight(kg)', 'height (cm)', 'hypertension duration (years)', 'Aldosterone (ng/dL)', 'PRA (ngml-1h-1)', 'SBP (mmHg)', 'dBP (mmHg)', 'Na+(\xa0mmol/L)', 'K+(\xa0mmol/L)', 'ever_Hyperthyroidism_0', 'ever_Hyperthyroidism_1', 'ever_COPD_0', 'ever_COPD_1', 'ever diabetics_0', 'ever diabetics_1', 'ever_myocardiac infarction_0', 'ever_myocardiac infarction_1', 'Sex_1', 'Sex_2', 'family history_0', 'family history_1', 'family history_2', 'total categories of antiHTN drugs_0', 'total categories of antiHTN drugs_1', 'total categories of antiHTN drugs_2', 'total categories of antiHTN drugs_5']

Factor_33:

['age', 'Body weight(kg)', 'height (cm)', 'hypertension duration (years)', 'Aldosterone (ng/dL)', 'PRA (ngml-1h-1)', 'SBP (mmHg)', 'dBP (mmHg)', 'Na+(\xa0mmol/L)', 'K+(\xa0mmol/L)', 'ever_Hyperthyroidism_0', 'ever_Hyperthyroidism_1', 'ever_COPD_0', 'ever_COPD_1', 'ever diabetics_0', 'ever diabetics_1', 'ever_myocardiac infarction_0', 'ever_myocardiac infarction_1', 'Sex_1', 'Sex_2', 'family history_0', 'family history_1', 'family history_2', 'total categories of antiHTN drugs_0', 'total categories of antiHTN drugs_1', 'total categories of antiHTN drugs_3', 'total categories of antiHTN drugs_4']

Factor_34:

['age', 'Body weight(kg)', 'height (cm)', 'hypertension duration (years)', 'Aldosterone (ng/dL)', 'PRA (ngml-1h-1)', 'SBP (mmHg)', 'dBP (mmHg)', 'Na+(\xa0mmol/L)', 'K+(\xa0mmol/L)', 'ever_Hyperthyroidism_0', 'ever_Hyperthyroidism_1', 'ever_COPD_0', 'ever_COPD_1', 'ever diabetics_0', 'ever diabetics_1', 'ever_myocardiac infarction_0', 'ever_myocardiac infarction_1', 'Sex_1', 'Sex_2', 'family history_0', 'family history_1', 'family history_2', 'total categories of antiHTN drugs_0', 'total categories of antiHTN drugs_1', 'total categories of antiHTN drugs_3', 'total categories of antiHTN drugs_5']

Factor_35:

['age', 'Body weight(kg)', 'height (cm)', 'hypertension duration (years)', 'Aldosterone (ng/dL)', 'PRA (ngml-1h-1)', 'SBP (mmHg)', 'dBP (mmHg)', 'Na+(\xa0mmol/L)', 'K+(\xa0mmol/L)', 'ever_Hyperthyroidism_0', 'ever_Hyperthyroidism_1', 'ever_COPD_0', 'ever_COPD_1', 'ever diabetics_0', 'ever diabetics_1', 'ever_myocardiac infarction_0', 'ever_myocardiac infarction_1', 'Sex_1', 'Sex_2', 'family history_0', 'family history_1', 'family history_2', 'total categories of antiHTN drugs_0', 'total categories of antiHTN drugs_1', 'total categories of antiHTN drugs_4', 'total categories of antiHTN drugs_5']

Factor_36:

['age', 'Body weight(kg)', 'height (cm)', 'hypertension duration (years)', 'Aldosterone (ng/dL)', 'PRA (ngml-1h-1)', 'SBP (mmHg)', 'dBP (mmHg)', 'Na+(\xa0mmol/L)', 'K+(\xa0mmol/L)', 'ever_Hyperthyroidism_0', 'ever_Hyperthyroidism_1', 'ever_COPD_0', 'ever_COPD_1', 'ever diabetics_0', 'ever diabetics_1', 'ever_myocardiac infarction_0', 'ever_myocardiac infarction_1', 'Sex_1', 'Sex_2', 'family history_0', 'family history_1', 'family history_2', 'total categories of antiHTN drugs_0', 'total categories of antiHTN drugs_2', 'total categories of antiHTN drugs_3', 'total categories of antiHTN drugs_4']

Factor_37:

['age', 'Body weight(kg)', 'height (cm)', 'hypertension duration (years)', 'Aldosterone (ng/dL)', 'PRA (ngml-1h-1)', 'SBP (mmHg)', 'dBP (mmHg)', 'Na+(\xa0mmol/L)', 'K+(\xa0mmol/L)', 'ever_Hyperthyroidism_0', 'ever_Hyperthyroidism_1', 'ever_COPD_0', 'ever_COPD_1', 'ever diabetics_0', 'ever diabetics_1', 'ever_myocardiac infarction_0', 'ever_myocardiac infarction_1', 'Sex_1', 'Sex_2', 'family history_0', 'family history_1', 'family history_2', 'total categories of antiHTN drugs_0', 'total categories of antiHTN drugs_2', 'total categories of antiHTN drugs_3', 'total categories of antiHTN drugs_5']

Factor_38:

['age', 'Body weight(kg)', 'height (cm)', 'hypertension duration (years)', 'Aldosterone (ng/dL)', 'PRA (ngml-1h-1)', 'SBP (mmHg)', 'dBP (mmHg)', 'Na+(\xa0mmol/L)', 'K+(\xa0mmol/L)', 'ever_Hyperthyroidism_0', 'ever_Hyperthyroidism_1', 'ever_COPD_0', 'ever_COPD_1', 'ever diabetics_0', 'ever diabetics_1', 'ever_myocardiac infarction_0', 'ever_myocardiac infarction_1', 'Sex_1', 'Sex_2', 'family history_0', 'family history_1', 'family history_2', 'total categories of antiHTN drugs_0', 'total categories of antiHTN drugs_2', 'total categories of antiHTN drugs_4', 'total categories of antiHTN drugs_5']

Factor_39:

['age', 'Body weight(kg)', 'height (cm)', 'hypertension duration (years)', 'Aldosterone (ng/dL)', 'PRA (ngml-1h-1)', 'SBP (mmHg)', 'dBP (mmHg)', 'Na+(\xa0mmol/L)', 'K+(\xa0mmol/L)', 'ever_Hyperthyroidism_0', 'ever_Hyperthyroidism_1', 'ever_COPD_0', 'ever_COPD_1', 'ever diabetics_0', 'ever diabetics_1', 'ever_myocardiac infarction_0', 'ever_myocardiac infarction_1', 'Sex_1', 'Sex_2', 'family history_0', 'family history_1', 'family history_2', 'total categories of antiHTN drugs_0', 'total categories of antiHTN drugs_3', 'total categories of antiHTN drugs_4', 'total categories of antiHTN drugs_5']

Factor_40:

['age', 'Body weight(kg)', 'height (cm)', 'hypertension duration (years)', 'Aldosterone (ng/dL)', 'PRA (ngml-1h-1)', 'SBP (mmHg)', 'dBP (mmHg)', 'Na+(\xa0mmol/L)', 'K+(\xa0mmol/L)', 'ever_Hyperthyroidism_0', 'ever_Hyperthyroidism_1', 'ever_COPD_0', 'ever_COPD_1', 'ever diabetics_0', 'ever diabetics_1', 'ever_myocardiac infarction_0', 'ever_myocardiac infarction_1', 'Sex_1', 'Sex_2', 'family history_0', 'family history_1', 'family history_2', 'total categories of antiHTN drugs_1', 'total categories of antiHTN drugs_2', 'total categories of antiHTN drugs_3', 'total categories of antiHTN drugs_4']

Factor_41:

['age', 'Body weight(kg)', 'height (cm)', 'hypertension duration (years)', 'Aldosterone (ng/dL)', 'PRA (ngml-1h-1)', 'SBP (mmHg)', 'dBP (mmHg)', 'Na+(\xa0mmol/L)', 'K+(\xa0mmol/L)', 'ever_Hyperthyroidism_0', 'ever_Hyperthyroidism_1', 'ever_COPD_0', 'ever_COPD_1', 'ever diabetics_0', 'ever diabetics_1', 'ever_myocardiac infarction_0', 'ever_myocardiac infarction_1', 'Sex_1', 'Sex_2', 'family history_0', 'family history_1', 'family history_2', 'total categories of antiHTN drugs_1', 'total categories of antiHTN drugs_2', 'total categories of antiHTN drugs_3', 'total categories of antiHTN drugs_5']

Factor_42:

['age', 'Body weight(kg)', 'height (cm)', 'hypertension duration (years)', 'Aldosterone (ng/dL)', 'PRA (ngml-1h-1)', 'SBP (mmHg)', 'dBP (mmHg)', 'Na+(\xa0mmol/L)', 'K+(\xa0mmol/L)', 'ever_Hyperthyroidism_0', 'ever_Hyperthyroidism_1', 'ever_COPD_0', 'ever_COPD_1', 'ever diabetics_0', 'ever diabetics_1', 'ever_myocardiac infarction_0', 'ever_myocardiac infarction_1', 'Sex_1', 'Sex_2', 'family history_0', 'family history_1', 'family history_2', 'total categories of antiHTN drugs_1', 'total categories of antiHTN drugs_2', 'total categories of antiHTN drugs_4', 'total categories of antiHTN drugs_5']

Factor_43:

['age', 'Body weight(kg)', 'height (cm)', 'hypertension duration (years)', 'Aldosterone (ng/dL)', 'PRA (ngml-1h-1)', 'SBP (mmHg)', 'dBP (mmHg)', 'Na+(\xa0mmol/L)', 'K+(\xa0mmol/L)', 'ever_Hyperthyroidism_0', 'ever_Hyperthyroidism_1', 'ever_COPD_0', 'ever_COPD_1', 'ever diabetics_0', 'ever diabetics_1', 'ever_myocardiac infarction_0', 'ever_myocardiac infarction_1', 'Sex_1', 'Sex_2', 'family history_0', 'family history_1', 'family history_2', 'total categories of antiHTN drugs_1', 'total categories of antiHTN drugs_3', 'total categories of antiHTN drugs_4', 'total categories of antiHTN drugs_5']

Factor_44:

['age', 'Body weight(kg)', 'height (cm)', 'hypertension duration (years)', 'Aldosterone (ng/dL)', 'PRA (ngml-1h-1)', 'SBP (mmHg)', 'dBP (mmHg)', 'Na+(\xa0mmol/L)', 'K+(\xa0mmol/L)', 'ever_Hyperthyroidism_0', 'ever_Hyperthyroidism_1', 'ever_COPD_0', 'ever_COPD_1', 'ever diabetics_0', 'ever diabetics_1', 'ever_myocardiac infarction_0', 'ever_myocardiac infarction_1', 'Sex_1', 'Sex_2', 'family history_0', 'family history_1', 'family history_2', 'total categories of antiHTN drugs_2', 'total categories of antiHTN drugs_3', 'total categories of antiHTN drugs_4', 'total categories of antiHTN drugs_5']

Factor_45:

['age', 'Body weight(kg)', 'height (cm)', 'hypertension duration (years)', 'Aldosterone (ng/dL)', 'PRA (ngml-1h-1)', 'SBP (mmHg)', 'dBP (mmHg)', 'Na+(\xa0mmol/L)', 'K+(\xa0mmol/L)', 'ever_Hyperthyroidism_0', 'ever_Hyperthyroidism_1', 'ever_COPD_0', 'ever_COPD_1', 'ever diabetics_0', 'ever diabetics_1', 'ever_myocardiac infarction_0', 'ever_myocardiac infarction_1', 'Sex_1', 'Sex_2', 'family history_0', 'family history_1', 'total categories of antiHTN drugs_0', 'total categories of antiHTN drugs_1', 'total categories of antiHTN drugs_2', 'total categories of antiHTN drugs_3', 'total categories of antiHTN drugs_4']

Factor_46:

['age', 'Body weight(kg)', 'height (cm)', 'hypertension duration (years)', 'Aldosterone (ng/dL)', 'PRA (ngml-1h-1)', 'SBP (mmHg)', 'dBP (mmHg)', 'Na+(\xa0mmol/L)', 'K+(\xa0mmol/L)', 'ever_Hyperthyroidism_0', 'ever_Hyperthyroidism_1', 'ever_COPD_0', 'ever_COPD_1', 'ever diabetics_0', 'ever diabetics_1', 'ever_myocardiac infarction_0', 'ever_myocardiac infarction_1', 'Sex_1', 'Sex_2', 'family history_0', 'family history_1', 'total categories of antiHTN drugs_0', 'total categories of antiHTN drugs_1', 'total categories of antiHTN drugs_2', 'total categories of antiHTN drugs_3', 'total categories of antiHTN drugs_5']

Factor_47:

['age', 'Body weight(kg)', 'height (cm)', 'hypertension duration (years)', 'Aldosterone (ng/dL)', 'PRA (ngml-1h-1)', 'SBP (mmHg)', 'dBP (mmHg)', 'Na+(\xa0mmol/L)', 'K+(\xa0mmol/L)', 'ever_Hyperthyroidism_0', 'ever_Hyperthyroidism_1', 'ever_COPD_0', 'ever_COPD_1', 'ever diabetics_0', 'ever diabetics_1', 'ever_myocardiac infarction_0', 'ever_myocardiac infarction_1', 'Sex_1', 'Sex_2', 'family history_0', 'family history_1', 'total categories of antiHTN drugs_0', 'total categories of antiHTN drugs_1', 'total categories of antiHTN drugs_2', 'total categories of antiHTN drugs_4', 'total categories of antiHTN drugs_5']

Factor_48:

['age', 'Body weight(kg)', 'height (cm)', 'hypertension duration (years)', 'Aldosterone (ng/dL)', 'PRA (ngml-1h-1)', 'SBP (mmHg)', 'dBP (mmHg)', 'Na+(\xa0mmol/L)', 'K+(\xa0mmol/L)', 'ever_Hyperthyroidism_0', 'ever_Hyperthyroidism_1', 'ever_COPD_0', 'ever_COPD_1', 'ever diabetics_0', 'ever diabetics_1', 'ever_myocardiac infarction_0', 'ever_myocardiac infarction_1', 'Sex_1', 'Sex_2', 'family history_0', 'family history_1', 'total categories of antiHTN drugs_0', 'total categories of antiHTN drugs_1', 'total categories of antiHTN drugs_3', 'total categories of antiHTN drugs_4', 'total categories of antiHTN drugs_5']

Factor_49:

['age', 'Body weight(kg)', 'height (cm)', 'hypertension duration (years)', 'Aldosterone (ng/dL)', 'PRA (ngml-1h-1)', 'SBP (mmHg)', 'dBP (mmHg)', 'Na+(\xa0mmol/L)', 'K+(\xa0mmol/L)', 'ever_Hyperthyroidism_0', 'ever_Hyperthyroidism_1', 'ever_COPD_0', 'ever_COPD_1', 'ever diabetics_0', 'ever diabetics_1', 'ever_myocardiac infarction_0', 'ever_myocardiac infarction_1', 'Sex_1', 'Sex_2', 'family history_0', 'family history_1', 'total categories of antiHTN drugs_0', 'total categories of antiHTN drugs_2', 'total categories of antiHTN drugs_3', 'total categories of antiHTN drugs_4', 'total categories of antiHTN drugs_5']

Factor_50:

['age', 'Body weight(kg)', 'height (cm)', 'hypertension duration (years)', 'Aldosterone (ng/dL)', 'PRA (ngml-1h-1)', 'SBP (mmHg)', 'dBP (mmHg)', 'Na+(\xa0mmol/L)', 'K+(\xa0mmol/L)', 'ever_Hyperthyroidism_0', 'ever_Hyperthyroidism_1', 'ever_COPD_0', 'ever_COPD_1', 'ever diabetics_0', 'ever diabetics_1', 'ever_myocardiac infarction_0', 'ever_myocardiac infarction_1', 'Sex_1', 'Sex_2', 'family history_0', 'family history_1', 'total categories of antiHTN drugs_1', 'total categories of antiHTN drugs_2', 'total categories of antiHTN drugs_3', 'total categories of antiHTN drugs_4', 'total categories of antiHTN drugs_5']

Factor_51:

['age', 'Body weight(kg)', 'height (cm)', 'hypertension duration (years)', 'Aldosterone (ng/dL)', 'PRA (ngml-1h-1)', 'SBP (mmHg)', 'dBP (mmHg)', 'Na+(\xa0mmol/L)', 'K+(\xa0mmol/L)', 'ever_Hyperthyroidism_0', 'ever_Hyperthyroidism_1', 'ever_COPD_0', 'ever_COPD_1', 'ever diabetics_0', 'ever diabetics_1', 'ever_myocardiac infarction_0', 'ever_myocardiac infarction_1', 'Sex_1', 'Sex_2', 'family history_0', 'family history_1', 'family history_2', 'total categories of antiHTN drugs_0', 'total categories of antiHTN drugs_1', 'total categories of antiHTN drugs_2', 'total categories of antiHTN drugs_3', 'total categories of antiHTN drugs_4', 'total categories of antiHTN drugs_5']

Factor_52:

['age', 'Body weight(kg)', 'height (cm)', 'hypertension duration (years)', 'Aldosterone (ng/dL)', 'PRA (ngml-1h-1)', 'SBP (mmHg)', 'dBP (mmHg)', 'Na+(\xa0mmol/L)', 'K+(\xa0mmol/L)', 'ever_Hyperthyroidism_0', 'ever_Hyperthyroidism_1', 'ever_COPD_0', 'ever_COPD_1', 'ever diabetics_0', 'ever diabetics_1', 'ever_myocardiac infarction_0', 'ever_myocardiac infarction_1', 'Sex_1', 'Sex_2', 'family history_0', 'family history_1', 'family history_2', 'total categories of antiHTN drugs_0', 'total categories of antiHTN drugs_1', 'total categories of antiHTN drugs_2', 'total categories of antiHTN drugs_3', 'total categories of antiHTN drugs_4']

Factor_53:

['age', 'Body weight(kg)', 'height (cm)', 'hypertension duration (years)', 'Aldosterone (ng/dL)', 'PRA (ngml-1h-1)', 'SBP (mmHg)', 'dBP (mmHg)', 'Na+(\xa0mmol/L)', 'K+(\xa0mmol/L)', 'ever_Hyperthyroidism_0', 'ever_Hyperthyroidism_1', 'ever_COPD_0', 'ever_COPD_1', 'ever diabetics_0', 'ever diabetics_1', 'ever_myocardiac infarction_0', 'ever_myocardiac infarction_1', 'Sex_1', 'Sex_2', 'family history_0', 'family history_1', 'family history_2', 'total categories of antiHTN drugs_0', 'total categories of antiHTN drugs_1', 'total categories of antiHTN drugs_2', 'total categories of antiHTN drugs_3', 'total categories of antiHTN drugs_5']

Factor_54:

['age', 'Body weight(kg)', 'height (cm)', 'hypertension duration (years)', 'Aldosterone (ng/dL)', 'PRA (ngml-1h-1)', 'SBP (mmHg)', 'dBP (mmHg)', 'Na+(\xa0mmol/L)', 'K+(\xa0mmol/L)', 'ever_Hyperthyroidism_0', 'ever_Hyperthyroidism_1', 'ever_COPD_0', 'ever_COPD_1', 'ever diabetics_0', 'ever diabetics_1', 'ever_myocardiac infarction_0', 'ever_myocardiac infarction_1', 'Sex_1', 'Sex_2', 'family history_0', 'family history_1', 'family history_2', 'total categories of antiHTN drugs_0', 'total categories of antiHTN drugs_1', 'total categories of antiHTN drugs_2', 'total categories of antiHTN drugs_4', 'total categories of antiHTN drugs_5']

Factor_55:

['age', 'Body weight(kg)', 'height (cm)', 'hypertension duration (years)', 'Aldosterone (ng/dL)', 'PRA (ngml-1h-1)', 'SBP (mmHg)', 'dBP (mmHg)', 'Na+(\xa0mmol/L)', 'K+(\xa0mmol/L)', 'ever_Hyperthyroidism_0', 'ever_Hyperthyroidism_1', 'ever_COPD_0', 'ever_COPD_1', 'ever diabetics_0', 'ever diabetics_1', 'ever_myocardiac infarction_0', 'ever_myocardiac infarction_1', 'Sex_1', 'Sex_2', 'family history_0', 'family history_1', 'family history_2', 'total categories of antiHTN drugs_0', 'total categories of antiHTN drugs_1', 'total categories of antiHTN drugs_3', 'total categories of antiHTN drugs_4', 'total categories of antiHTN drugs_5']

Factor_56:

['age', 'Body weight(kg)', 'height (cm)', 'hypertension duration (years)', 'Aldosterone (ng/dL)', 'PRA (ngml-1h-1)', 'SBP (mmHg)', 'dBP (mmHg)', 'Na+(\xa0mmol/L)', 'K+(\xa0mmol/L)', 'ever_Hyperthyroidism_0', 'ever_Hyperthyroidism_1', 'ever_COPD_0', 'ever_COPD_1', 'ever diabetics_0', 'ever diabetics_1', 'ever_myocardiac infarction_0', 'ever_myocardiac infarction_1', 'Sex_1', 'Sex_2', 'family history_0', 'family history_1', 'family history_2', 'total categories of antiHTN drugs_0', 'total categories of antiHTN drugs_2', 'total categories of antiHTN drugs_3', 'total categories of antiHTN drugs_4', 'total categories of antiHTN drugs_5']

Factor_57:

['age', 'Body weight(kg)', 'height (cm)', 'hypertension duration (years)', 'Aldosterone (ng/dL)', 'PRA (ngml-1h-1)', 'SBP (mmHg)', 'dBP (mmHg)', 'Na+(\xa0mmol/L)', 'K+(\xa0mmol/L)', 'ever_Hyperthyroidism_0', 'ever_Hyperthyroidism_1', 'ever_COPD_0', 'ever_COPD_1', 'ever diabetics_0', 'ever diabetics_1', 'ever_myocardiac infarction_0', 'ever_myocardiac infarction_1', 'Sex_1', 'Sex_2', 'family history_0', 'family history_1', 'family history_2', 'total categories of antiHTN drugs_1', 'total categories of antiHTN drugs_2', 'total categories of antiHTN drugs_3', 'total categories of antiHTN drugs_4', 'total categories of antiHTN drugs_5']

Factor_58:

['age', 'Body weight(kg)', 'height (cm)', 'hypertension duration (years)', 'Aldosterone (ng/dL)', 'PRA (ngml-1h-1)', 'SBP (mmHg)', 'dBP (mmHg)', 'Na+(\xa0mmol/L)', 'K+(\xa0mmol/L)', 'ever_Hyperthyroidism_0', 'ever_Hyperthyroidism_1', 'ever_COPD_0', 'ever_COPD_1', 'ever diabetics_0', 'ever diabetics_1', 'ever_myocardiac infarction_0', 'ever_myocardiac infarction_1', 'Sex_1', 'Sex_2', 'family history_0', 'family history_1', 'total categories of antiHTN drugs_0', 'total categories of antiHTN drugs_1', 'total categories of antiHTN drugs_2', 'total categories of antiHTN drugs_3', 'total categories of antiHTN drugs_4', 'total categories of antiHTN drugs_5']

Factor_59:

['age', 'Body weight(kg)', 'height (cm)', 'hypertension duration (years)', 'Aldosterone (ng/dL)', 'PRA (ngml-1h-1)', 'SBP (mmHg)', 'dBP (mmHg)', 'Na+(\xa0mmol/L)', 'K+(\xa0mmol/L)', 'ever_Hyperthyroidism_0', 'ever_Hyperthyroidism_1', 'ever_COPD_0', 'ever_COPD_1', 'ever diabetics_0', 'ever diabetics_1', 'ever_myocardiac infarction_0', 'ever_myocardiac infarction_1', 'Sex_1', 'Sex_2', 'family history_0', 'family history_2', 'total categories of antiHTN drugs_0', 'total categories of antiHTN drugs_1', 'total categories of antiHTN drugs_2', 'total categories of antiHTN drugs_3', 'total categories of antiHTN drugs_4', 'total categories of antiHTN drugs_5']

Factor_60:

['age', 'Body weight(kg)', 'height (cm)', 'hypertension duration (years)', 'Aldosterone (ng/dL)', 'PRA (ngml-1h-1)', 'SBP (mmHg)', 'dBP (mmHg)', 'Na+(\xa0mmol/L)', 'K+(\xa0mmol/L)', 'ever_Hyperthyroidism_0', 'ever_Hyperthyroidism_1', 'ever_COPD_0', 'ever_COPD_1', 'ever diabetics_0', 'ever diabetics_1', 'ever_myocardiac infarction_0', 'ever_myocardiac infarction_1', 'Sex_1', 'Sex_2', 'family history_1', 'family history_2', 'total categories of antiHTN drugs_0', 'total categories of antiHTN drugs_1', 'total categories of antiHTN drugs_2', 'total categories of antiHTN drugs_3', 'total categories of antiHTN drugs_4', 'total categories of antiHTN drugs_5']

Factor_61:

['age', 'Body weight(kg)', 'height (cm)', 'hypertension duration (years)', 'Aldosterone (ng/dL)', 'PRA (ngml-1h-1)', 'SBP (mmHg)', 'dBP (mmHg)', 'Na+(\xa0mmol/L)', 'K+(\xa0mmol/L)', 'ever_Hyperthyroidism_0', 'ever_Hyperthyroidism_1', 'ever_COPD_0', 'ever_COPD_1', 'ever diabetics_0', 'ever diabetics_1', 'ever_myocardiac infarction_0', 'ever_myocardiac infarction_1', 'Sex_1', 'family history_0', 'family history_1', 'family history_2', 'total categories of antiHTN drugs_0', 'total categories of antiHTN drugs_1', 'total categories of antiHTN drugs_2', 'total categories of antiHTN drugs_3', 'total categories of antiHTN drugs_4', 'total categories of antiHTN drugs_5']

Factor_62:

['age', 'Body weight(kg)', 'height (cm)', 'hypertension duration (years)', 'Aldosterone (ng/dL)', 'PRA (ngml-1h-1)', 'SBP (mmHg)', 'dBP (mmHg)', 'Na+(\xa0mmol/L)', 'K+(\xa0mmol/L)', 'ever_Hyperthyroidism_0', 'ever_Hyperthyroidism_1', 'ever_COPD_0', 'ever_COPD_1', 'ever diabetics_0', 'ever diabetics_1', 'ever_myocardiac infarction_0', 'ever_myocardiac infarction_1', 'Sex_2', 'family history_0', 'family history_1', 'family history_2', 'total categories of antiHTN drugs_0', 'total categories of antiHTN drugs_1', 'total categories of antiHTN drugs_2', 'total categories of antiHTN drugs_3', 'total categories of antiHTN drugs_4', 'total categories of antiHTN drugs_5']

Factor_63:

['age', 'Body weight(kg)', 'height (cm)', 'hypertension duration (years)', 'Aldosterone (ng/dL)', 'PRA (ngml-1h-1)', 'SBP (mmHg)', 'dBP (mmHg)', 'Na+(\xa0mmol/L)', 'K+(\xa0mmol/L)', 'ever_Hyperthyroidism_0', 'ever_Hyperthyroidism_1', 'ever_COPD_0', 'ever_COPD_1', 'ever diabetics_0', 'ever diabetics_1', 'ever_myocardiac infarction_0', 'Sex_1', 'Sex_2', 'family history_0', 'family history_1', 'family history_2', 'total categories of antiHTN drugs_0', 'total categories of antiHTN drugs_1', 'total categories of antiHTN drugs_2', 'total categories of antiHTN drugs_3', 'total categories of antiHTN drugs_4', 'total categories of antiHTN drugs_5']

Factor_64:

['age', 'Body weight(kg)', 'height (cm)', 'hypertension duration (years)', 'Aldosterone (ng/dL)', 'PRA (ngml-1h-1)', 'SBP (mmHg)', 'dBP (mmHg)', 'Na+(\xa0mmol/L)', 'K+(\xa0mmol/L)', 'ever_Hyperthyroidism_0', 'ever_Hyperthyroidism_1', 'ever_COPD_0', 'ever_COPD_1', 'ever diabetics_0', 'ever diabetics_1', 'ever_myocardiac infarction_1', 'Sex_1', 'Sex_2', 'family history_0', 'family history_1', 'family history_2', 'total categories of antiHTN drugs_0', 'total categories of antiHTN drugs_1', 'total categories of antiHTN drugs_2', 'total categories of antiHTN drugs_3', 'total categories of antiHTN drugs_4', 'total categories of antiHTN drugs_5']

Factor_65:

['age', 'Body weight(kg)', 'height (cm)', 'hypertension duration (years)', 'Aldosterone (ng/dL)', 'PRA (ngml-1h-1)', 'SBP (mmHg)', 'dBP (mmHg)', 'Na+(\xa0mmol/L)', 'K+(\xa0mmol/L)', 'ever_Hyperthyroidism_0', 'ever_Hyperthyroidism_1', 'ever_COPD_0', 'ever_COPD_1', 'ever diabetics_0', 'ever_myocardiac infarction_0', 'ever_myocardiac infarction_1', 'Sex_1', 'Sex_2', 'family history_0', 'family history_1', 'family history_2', 'total categories of antiHTN drugs_0', 'total categories of antiHTN drugs_1', 'total categories of antiHTN drugs_2', 'total categories of antiHTN drugs_3', 'total categories of antiHTN drugs_4', 'total categories of antiHTN drugs_5']

Factor_66:

['age', 'Body weight(kg)', 'height (cm)', 'hypertension duration (years)', 'Aldosterone (ng/dL)', 'PRA (ngml-1h-1)', 'SBP (mmHg)', 'dBP (mmHg)', 'Na+(\xa0mmol/L)', 'K+(\xa0mmol/L)', 'ever_Hyperthyroidism_0', 'ever_Hyperthyroidism_1', 'ever_COPD_0', 'ever_COPD_1', 'ever diabetics_1', 'ever_myocardiac infarction_0', 'ever_myocardiac infarction_1', 'Sex_1', 'Sex_2', 'family history_0', 'family history_1', 'family history_2', 'total categories of antiHTN drugs_0', 'total categories of antiHTN drugs_1', 'total categories of antiHTN drugs_2', 'total categories of antiHTN drugs_3', 'total categories of antiHTN drugs_4', 'total categories of antiHTN drugs_5']

Factor_67:

['age', 'Body weight(kg)', 'height (cm)', 'hypertension duration (years)', 'Aldosterone (ng/dL)', 'PRA (ngml-1h-1)', 'SBP (mmHg)', 'dBP (mmHg)', 'Na+(\xa0mmol/L)', 'K+(\xa0mmol/L)', 'ever_Hyperthyroidism_0', 'ever_Hyperthyroidism_1', 'ever_COPD_0', 'ever diabetics_0', 'ever diabetics_1', 'ever_myocardiac infarction_0', 'ever_myocardiac infarction_1', 'Sex_1', 'Sex_2', 'family history_0', 'family history_1', 'family history_2', 'total categories of antiHTN drugs_0', 'total categories of antiHTN drugs_1', 'total categories of antiHTN drugs_2', 'total categories of antiHTN drugs_3', 'total categories of antiHTN drugs_4', 'total categories of antiHTN drugs_5']

Factor_68:

['age', 'Body weight(kg)', 'height (cm)', 'hypertension duration (years)', 'Aldosterone (ng/dL)', 'PRA (ngml-1h-1)', 'SBP (mmHg)', 'dBP (mmHg)', 'Na+(\xa0mmol/L)', 'K+(\xa0mmol/L)', 'ever_Hyperthyroidism_0', 'ever_Hyperthyroidism_1', 'ever_COPD_1', 'ever diabetics_0', 'ever diabetics_1', 'ever_myocardiac infarction_0', 'ever_myocardiac infarction_1', 'Sex_1', 'Sex_2', 'family history_0', 'family history_1', 'family history_2', 'total categories of antiHTN drugs_0', 'total categories of antiHTN drugs_1', 'total categories of antiHTN drugs_2', 'total categories of antiHTN drugs_3', 'total categories of antiHTN drugs_4', 'total categories of antiHTN drugs_5']

Factor_69:

['age', 'Body weight(kg)', 'height (cm)', 'hypertension duration (years)', 'Aldosterone (ng/dL)', 'PRA (ngml-1h-1)', 'SBP (mmHg)', 'dBP (mmHg)', 'Na+(\xa0mmol/L)', 'K+(\xa0mmol/L)', 'ever_Hyperthyroidism_0', 'ever_COPD_0', 'ever_COPD_1', 'ever diabetics_0', 'ever diabetics_1', 'ever_myocardiac infarction_0', 'ever_myocardiac infarction_1', 'Sex_1', 'Sex_2', 'family history_0', 'family history_1', 'family history_2', 'total categories of antiHTN drugs_0', 'total categories of antiHTN drugs_1', 'total categories of antiHTN drugs_2', 'total categories of antiHTN drugs_3', 'total categories of antiHTN drugs_4', 'total categories of antiHTN drugs_5']

Factor_70:

['age', 'Body weight(kg)', 'height (cm)', 'hypertension duration (years)', 'Aldosterone (ng/dL)', 'PRA (ngml-1h-1)', 'SBP (mmHg)', 'dBP (mmHg)', 'Na+(\xa0mmol/L)', 'K+(\xa0mmol/L)', 'ever_Hyperthyroidism_1', 'ever_COPD_0', 'ever_COPD_1', 'ever diabetics_0', 'ever diabetics_1', 'ever_myocardiac infarction_0', 'ever_myocardiac infarction_1', 'Sex_1', 'Sex_2', 'family history_0', 'family history_1', 'family history_2', 'total categories of antiHTN drugs_0', 'total categories of antiHTN drugs_1', 'total categories of antiHTN drugs_2', 'total categories of antiHTN drugs_3', 'total categories of antiHTN drugs_4', 'total categories of antiHTN drugs_5']

Factor_71:

['age', 'Body weight(kg)', 'height (cm)', 'hypertension duration (years)', 'Aldosterone (ng/dL)', 'PRA (ngml-1h-1)', 'SBP (mmHg)', 'dBP (mmHg)', 'Na+(\xa0mmol/L)', 'ever_Hyperthyroidism_0', 'ever_Hyperthyroidism_1', 'ever_COPD_0', 'ever_COPD_1', 'ever diabetics_0', 'ever diabetics_1', 'ever_myocardiac infarction_0', 'ever_myocardiac infarction_1', 'Sex_1', 'Sex_2', 'family history_0', 'family history_1', 'family history_2', 'total categories of antiHTN drugs_0', 'total categories of antiHTN drugs_1', 'total categories of antiHTN drugs_2', 'total categories of antiHTN drugs_3', 'total categories of antiHTN drugs_4', 'total categories of antiHTN drugs_5']

Factor_72:

['age', 'Body weight(kg)', 'height (cm)', 'hypertension duration (years)', 'Aldosterone (ng/dL)', 'PRA (ngml-1h-1)', 'SBP (mmHg)', 'dBP (mmHg)', 'K+(\xa0mmol/L)', 'ever_Hyperthyroidism_0', 'ever_Hyperthyroidism_1', 'ever_COPD_0', 'ever_COPD_1', 'ever diabetics_0', 'ever diabetics_1', 'ever_myocardiac infarction_0', 'ever_myocardiac infarction_1', 'Sex_1', 'Sex_2', 'family history_0', 'family history_1', 'family history_2', 'total categories of antiHTN drugs_0', 'total categories of antiHTN drugs_1', 'total categories of antiHTN drugs_2', 'total categories of antiHTN drugs_3', 'total categories of antiHTN drugs_4', 'total categories of antiHTN drugs_5']

Factor_73:

['age', 'Body weight(kg)', 'height (cm)', 'hypertension duration (years)', 'Aldosterone (ng/dL)', 'PRA (ngml-1h-1)', 'SBP (mmHg)', 'Na+(\xa0mmol/L)', 'K+(\xa0mmol/L)', 'ever_Hyperthyroidism_0', 'ever_Hyperthyroidism_1', 'ever_COPD_0', 'ever_COPD_1', 'ever diabetics_0', 'ever diabetics_1', 'ever_myocardiac infarction_0', 'ever_myocardiac infarction_1', 'Sex_1', 'Sex_2', 'family history_0', 'family history_1', 'family history_2', 'total categories of antiHTN drugs_0', 'total categories of antiHTN drugs_1', 'total categories of antiHTN drugs_2', 'total categories of antiHTN drugs_3', 'total categories of antiHTN drugs_4', 'total categories of antiHTN drugs_5']

Factor_74:

['age', 'Body weight(kg)', 'height (cm)', 'hypertension duration (years)', 'Aldosterone (ng/dL)', 'PRA (ngml-1h-1)', 'dBP (mmHg)', 'Na+(\xa0mmol/L)', 'K+(\xa0mmol/L)', 'ever_Hyperthyroidism_0', 'ever_Hyperthyroidism_1', 'ever_COPD_0', 'ever_COPD_1', 'ever diabetics_0', 'ever diabetics_1', 'ever_myocardiac infarction_0', 'ever_myocardiac infarction_1', 'Sex_1', 'Sex_2', 'family history_0', 'family history_1', 'family history_2', 'total categories of antiHTN drugs_0', 'total categories of antiHTN drugs_1', 'total categories of antiHTN drugs_2', 'total categories of antiHTN drugs_3', 'total categories of antiHTN drugs_4', 'total categories of antiHTN drugs_5']

Factor_75:

['age', 'Body weight(kg)', 'height (cm)', 'hypertension duration (years)', 'PRA (ngml-1h-1)', 'SBP (mmHg)', 'dBP (mmHg)', 'Na+(\xa0mmol/L)', 'K+(\xa0mmol/L)', 'ever_Hyperthyroidism_0', 'ever_Hyperthyroidism_1', 'ever_COPD_0', 'ever_COPD_1', 'ever diabetics_0', 'ever diabetics_1', 'ever_myocardiac infarction_0', 'ever_myocardiac infarction_1', 'Sex_1', 'Sex_2', 'family history_0', 'family history_1', 'family history_2', 'total categories of antiHTN drugs_0', 'total categories of antiHTN drugs_1', 'total categories of antiHTN drugs_2', 'total categories of antiHTN drugs_3', 'total categories of antiHTN drugs_4', 'total categories of antiHTN drugs_5']

Factor_76:

['age', 'Body weight(kg)', 'height (cm)', 'Aldosterone (ng/dL)', 'PRA (ngml-1h-1)', 'SBP (mmHg)', 'dBP (mmHg)', 'Na+(\xa0mmol/L)', 'K+(\xa0mmol/L)', 'ever_Hyperthyroidism_0', 'ever_Hyperthyroidism_1', 'ever_COPD_0', 'ever_COPD_1', 'ever diabetics_0', 'ever diabetics_1', 'ever_myocardiac infarction_0', 'ever_myocardiac infarction_1', 'Sex_1', 'Sex_2', 'family history_0', 'family history_1', 'family history_2', 'total categories of antiHTN drugs_0', 'total categories of antiHTN drugs_1', 'total categories of antiHTN drugs_2', 'total categories of antiHTN drugs_3', 'total categories of antiHTN drugs_4', 'total categories of antiHTN drugs_5']

Factor_77:

['age', 'Body weight(kg)', 'hypertension duration (years)', 'Aldosterone (ng/dL)', 'PRA (ngml-1h-1)', 'SBP (mmHg)', 'dBP (mmHg)', 'Na+(\xa0mmol/L)', 'K+(\xa0mmol/L)', 'ever_Hyperthyroidism_0', 'ever_Hyperthyroidism_1', 'ever_COPD_0', 'ever_COPD_1', 'ever diabetics_0', 'ever diabetics_1', 'ever_myocardiac infarction_0', 'ever_myocardiac infarction_1', 'Sex_1', 'Sex_2', 'family history_0', 'family history_1', 'family history_2', 'total categories of antiHTN drugs_0', 'total categories of antiHTN drugs_1', 'total categories of antiHTN drugs_2', 'total categories of antiHTN drugs_3', 'total categories of antiHTN drugs_4', 'total categories of antiHTN drugs_5']

Factor_78:

['age', 'height (cm)', 'hypertension duration (years)', 'Aldosterone (ng/dL)', 'PRA (ngml-1h-1)', 'SBP (mmHg)', 'dBP (mmHg)', 'Na+(\xa0mmol/L)', 'K+(\xa0mmol/L)', 'ever_Hyperthyroidism_0', 'ever_Hyperthyroidism_1', 'ever_COPD_0', 'ever_COPD_1', 'ever diabetics_0', 'ever diabetics_1', 'ever_myocardiac infarction_0', 'ever_myocardiac infarction_1', 'Sex_1', 'Sex_2', 'family history_0', 'family history_1', 'family history_2', 'total categories of antiHTN drugs_0', 'total categories of antiHTN drugs_1', 'total categories of antiHTN drugs_2', 'total categories of antiHTN drugs_3', 'total categories of antiHTN drugs_4', 'total categories of antiHTN drugs_5']

Factor_79:

['Body weight(kg)', 'height (cm)', 'hypertension duration (years)', 'Aldosterone (ng/dL)', 'PRA (ngml-1h-1)', 'SBP (mmHg)', 'dBP (mmHg)', 'Na+(\xa0mmol/L)', 'K+(\xa0mmol/L)', 'ever_Hyperthyroidism_0', 'ever_Hyperthyroidism_1', 'ever_COPD_0', 'ever_COPD_1', 'ever diabetics_0', 'ever diabetics_1', 'ever_myocardiac infarction_0', 'ever_myocardiac infarction_1', 'Sex_1', 'Sex_2', 'family history_0', 'family history_1', 'family history_2', 'total categories of antiHTN drugs_0', 'total categories of antiHTN drugs_1', 'total categories of antiHTN drugs_2', 'total categories of antiHTN drugs_3', 'total categories of antiHTN drugs_4', 'total categories of antiHTN drugs_5']

Factor_80:

['age', 'Body weight(kg)', 'height (cm)', 'hypertension duration (years)', 'Aldosterone (ng/dL)', 'PRA (ngml-1h-1)', 'SBP (mmHg)', 'dBP (mmHg)', 'Na+(\xa0mmol/L)', 'K+(\xa0mmol/L)', 'ever_Hyperthyroidism_0', 'ever_Hyperthyroidism_1', 'ever_COPD_0', 'ever_COPD_1', 'ever diabetics_0', 'ever diabetics_1', 'ever_myocardiac infarction_0', 'ever_myocardiac infarction_1', 'Sex_1', 'Sex_2', 'family history_0', 'family history_1', 'family history_2', 'total categories of antiHTN drugs_0', 'total categories of antiHTN drugs_1', 'total categories of antiHTN drugs_2', 'total categories of antiHTN drugs_3']

Factor_81:

['age', 'Body weight(kg)', 'height (cm)', 'hypertension duration (years)', 'Aldosterone (ng/dL)', 'PRA (ngml-1h-1)', 'SBP (mmHg)', 'dBP (mmHg)', 'Na+(\xa0mmol/L)', 'K+(\xa0mmol/L)', 'ever_Hyperthyroidism_0', 'ever_Hyperthyroidism_1', 'ever_COPD_0', 'ever_COPD_1', 'ever diabetics_0', 'ever diabetics_1', 'ever_myocardiac infarction_0', 'ever_myocardiac infarction_1', 'Sex_1', 'Sex_2', 'family history_0', 'family history_1', 'family history_2', 'total categories of antiHTN drugs_0', 'total categories of antiHTN drugs_1', 'total categories of antiHTN drugs_2', 'total categories of antiHTN drugs_4']

Factor_82:

['age', 'Body weight(kg)', 'height (cm)', 'hypertension duration (years)', 'Aldosterone (ng/dL)', 'PRA (ngml-1h-1)', 'SBP (mmHg)', 'dBP (mmHg)', 'Na+(\xa0mmol/L)', 'K+(\xa0mmol/L)', 'ever_Hyperthyroidism_0', 'ever_Hyperthyroidism_1', 'ever_COPD_0', 'ever_COPD_1', 'ever diabetics_0', 'ever diabetics_1', 'ever_myocardiac infarction_0', 'ever_myocardiac infarction_1', 'Sex_1', 'Sex_2', 'family history_0', 'family history_1', 'family history_2', 'total categories of antiHTN drugs_0', 'total categories of antiHTN drugs_1', 'total categories of antiHTN drugs_2', 'total categories of antiHTN drugs_5']

Factor_83:

['age', 'Body weight(kg)', 'height (cm)', 'hypertension duration (years)', 'Aldosterone (ng/dL)', 'PRA (ngml-1h-1)', 'SBP (mmHg)', 'dBP (mmHg)', 'Na+(\xa0mmol/L)', 'K+(\xa0mmol/L)', 'ever_Hyperthyroidism_0', 'ever_Hyperthyroidism_1', 'ever_COPD_0', 'ever_COPD_1', 'ever diabetics_0', 'ever diabetics_1', 'ever_myocardiac infarction_0', 'ever_myocardiac infarction_1', 'Sex_1', 'Sex_2', 'family history_0', 'family history_1', 'family history_2', 'total categories of antiHTN drugs_0', 'total categories of antiHTN drugs_1', 'total categories of antiHTN drugs_3', 'total categories of antiHTN drugs_4']

Factor_84:

['age', 'Body weight(kg)', 'height (cm)', 'hypertension duration (years)', 'Aldosterone (ng/dL)', 'PRA (ngml-1h-1)', 'SBP (mmHg)', 'dBP (mmHg)', 'Na+(\xa0mmol/L)', 'K+(\xa0mmol/L)', 'ever_Hyperthyroidism_0', 'ever_Hyperthyroidism_1', 'ever_COPD_0', 'ever_COPD_1', 'ever diabetics_0', 'ever diabetics_1', 'ever_myocardiac infarction_0', 'ever_myocardiac infarction_1', 'Sex_1', 'Sex_2', 'family history_0', 'family history_1', 'family history_2', 'total categories of antiHTN drugs_0', 'total categories of antiHTN drugs_1', 'total categories of antiHTN drugs_3', 'total categories of antiHTN drugs_5']

Factor_85:

['age', 'Body weight(kg)', 'height (cm)', 'hypertension duration (years)', 'Aldosterone (ng/dL)', 'PRA (ngml-1h-1)', 'SBP (mmHg)', 'dBP (mmHg)', 'Na+(\xa0mmol/L)', 'K+(\xa0mmol/L)', 'ever_Hyperthyroidism_0', 'ever_Hyperthyroidism_1', 'ever_COPD_0', 'ever_COPD_1', 'ever diabetics_0', 'ever diabetics_1', 'ever_myocardiac infarction_0', 'ever_myocardiac infarction_1', 'Sex_1', 'Sex_2', 'family history_0', 'family history_1', 'family history_2', 'total categories of antiHTN drugs_0', 'total categories of antiHTN drugs_1', 'total categories of antiHTN drugs_4', 'total categories of antiHTN drugs_5']

Factor_86:

['age', 'Body weight(kg)', 'height (cm)', 'hypertension duration (years)', 'Aldosterone (ng/dL)', 'PRA (ngml-1h-1)', 'SBP (mmHg)', 'dBP (mmHg)', 'Na+(\xa0mmol/L)', 'K+(\xa0mmol/L)', 'ever_Hyperthyroidism_0', 'ever_Hyperthyroidism_1', 'ever_COPD_0', 'ever_COPD_1', 'ever diabetics_0', 'ever diabetics_1', 'ever_myocardiac infarction_0', 'ever_myocardiac infarction_1', 'Sex_1', 'Sex_2', 'family history_0', 'family history_1', 'family history_2', 'total categories of antiHTN drugs_0', 'total categories of antiHTN drugs_2', 'total categories of antiHTN drugs_3', 'total categories of antiHTN drugs_4']

Factor_87:

['age', 'Body weight(kg)', 'height (cm)', 'hypertension duration (years)', 'Aldosterone (ng/dL)', 'PRA (ngml-1h-1)', 'SBP (mmHg)', 'dBP (mmHg)', 'Na+(\xa0mmol/L)', 'K+(\xa0mmol/L)', 'ever_Hyperthyroidism_0', 'ever_Hyperthyroidism_1', 'ever_COPD_0', 'ever_COPD_1', 'ever diabetics_0', 'ever diabetics_1', 'ever_myocardiac infarction_0', 'ever_myocardiac infarction_1', 'Sex_1', 'Sex_2', 'family history_0', 'family history_1', 'family history_2', 'total categories of antiHTN drugs_0', 'total categories of antiHTN drugs_2', 'total categories of antiHTN drugs_3', 'total categories of antiHTN drugs_5']

Factor_88:

['age', 'Body weight(kg)', 'height (cm)', 'hypertension duration (years)', 'Aldosterone (ng/dL)', 'PRA (ngml-1h-1)', 'SBP (mmHg)', 'dBP (mmHg)', 'Na+(\xa0mmol/L)', 'K+(\xa0mmol/L)', 'ever_Hyperthyroidism_0', 'ever_Hyperthyroidism_1', 'ever_COPD_0', 'ever_COPD_1', 'ever diabetics_0', 'ever diabetics_1', 'ever_myocardiac infarction_0', 'ever_myocardiac infarction_1', 'Sex_1', 'Sex_2', 'family history_0', 'family history_1', 'family history_2', 'total categories of antiHTN drugs_0', 'total categories of antiHTN drugs_2', 'total categories of antiHTN drugs_4', 'total categories of antiHTN drugs_5']

Factor_89:

['age', 'Body weight(kg)', 'height (cm)', 'hypertension duration (years)', 'Aldosterone (ng/dL)', 'PRA (ngml-1h-1)', 'SBP (mmHg)', 'dBP (mmHg)', 'Na+(\xa0mmol/L)', 'K+(\xa0mmol/L)', 'ever_Hyperthyroidism_0', 'ever_Hyperthyroidism_1', 'ever_COPD_0', 'ever_COPD_1', 'ever diabetics_0', 'ever diabetics_1', 'ever_myocardiac infarction_0', 'ever_myocardiac infarction_1', 'Sex_1', 'Sex_2', 'family history_0', 'family history_1', 'family history_2', 'total categories of antiHTN drugs_0', 'total categories of antiHTN drugs_3', 'total categories of antiHTN drugs_4', 'total categories of antiHTN drugs_5']

Factor_90:

['age', 'Body weight(kg)', 'height (cm)', 'hypertension duration (years)', 'Aldosterone (ng/dL)', 'PRA (ngml-1h-1)', 'SBP (mmHg)', 'dBP (mmHg)', 'Na+(\xa0mmol/L)', 'K+(\xa0mmol/L)', 'ever_Hyperthyroidism_0', 'ever_Hyperthyroidism_1', 'ever_COPD_0', 'ever_COPD_1', 'ever diabetics_0', 'ever diabetics_1', 'ever_myocardiac infarction_0', 'ever_myocardiac infarction_1', 'Sex_1', 'Sex_2', 'family history_0', 'family history_1', 'family history_2', 'total categories of antiHTN drugs_1', 'total categories of antiHTN drugs_2', 'total categories of antiHTN drugs_3', 'total categories of antiHTN drugs_4']

Factor_91:

['age', 'Body weight(kg)', 'height (cm)', 'hypertension duration (years)', 'Aldosterone (ng/dL)', 'PRA (ngml-1h-1)', 'SBP (mmHg)', 'dBP (mmHg)', 'Na+(\xa0mmol/L)', 'K+(\xa0mmol/L)', 'ever_Hyperthyroidism_0', 'ever_Hyperthyroidism_1', 'ever_COPD_0', 'ever_COPD_1', 'ever diabetics_0', 'ever diabetics_1', 'ever_myocardiac infarction_0', 'ever_myocardiac infarction_1', 'Sex_1', 'Sex_2', 'family history_0', 'family history_1', 'family history_2', 'total categories of antiHTN drugs_1', 'total categories of antiHTN drugs_2', 'total categories of antiHTN drugs_3', 'total categories of antiHTN drugs_5']

Factor_92:

['age', 'Body weight(kg)', 'height (cm)', 'hypertension duration (years)', 'Aldosterone (ng/dL)', 'PRA (ngml-1h-1)', 'SBP (mmHg)', 'dBP (mmHg)', 'Na+(\xa0mmol/L)', 'K+(\xa0mmol/L)', 'ever_Hyperthyroidism_0', 'ever_Hyperthyroidism_1', 'ever_COPD_0', 'ever_COPD_1', 'ever diabetics_0', 'ever diabetics_1', 'ever_myocardiac infarction_0', 'ever_myocardiac infarction_1', 'Sex_1', 'Sex_2', 'family history_0', 'family history_1', 'family history_2', 'total categories of antiHTN drugs_1', 'total categories of antiHTN drugs_2', 'total categories of antiHTN drugs_4', 'total categories of antiHTN drugs_5']

Factor_93:

['age', 'Body weight(kg)', 'height (cm)', 'hypertension duration (years)', 'Aldosterone (ng/dL)', 'PRA (ngml-1h-1)', 'SBP (mmHg)', 'dBP (mmHg)', 'Na+(\xa0mmol/L)', 'K+(\xa0mmol/L)', 'ever_Hyperthyroidism_0', 'ever_Hyperthyroidism_1', 'ever_COPD_0', 'ever_COPD_1', 'ever diabetics_0', 'ever diabetics_1', 'ever_myocardiac infarction_0', 'ever_myocardiac infarction_1', 'Sex_1', 'Sex_2', 'family history_0', 'family history_1', 'family history_2', 'total categories of antiHTN drugs_1', 'total categories of antiHTN drugs_3', 'total categories of antiHTN drugs_4', 'total categories of antiHTN drugs_5']

Factor_94:

['age', 'Body weight(kg)', 'height (cm)', 'hypertension duration (years)', 'Aldosterone (ng/dL)', 'PRA (ngml-1h-1)', 'SBP (mmHg)', 'dBP (mmHg)', 'Na+(\xa0mmol/L)', 'K+(\xa0mmol/L)', 'ever_Hyperthyroidism_0', 'ever_Hyperthyroidism_1', 'ever_COPD_0', 'ever_COPD_1', 'ever diabetics_0', 'ever diabetics_1', 'ever_myocardiac infarction_0', 'ever_myocardiac infarction_1', 'Sex_1', 'Sex_2', 'family history_0', 'family history_1', 'family history_2', 'total categories of antiHTN drugs_2', 'total categories of antiHTN drugs_3', 'total categories of antiHTN drugs_4', 'total categories of antiHTN drugs_5']

Factor_95:

['age', 'Body weight(kg)', 'height (cm)', 'hypertension duration (years)', 'Aldosterone (ng/dL)', 'PRA (ngml-1h-1)', 'SBP (mmHg)', 'dBP (mmHg)', 'Na+(\xa0mmol/L)', 'K+(\xa0mmol/L)', 'ever_Hyperthyroidism_0', 'ever_Hyperthyroidism_1', 'ever_COPD_0', 'ever_COPD_1', 'ever diabetics_0', 'ever diabetics_1', 'ever_myocardiac infarction_0', 'ever_myocardiac infarction_1', 'Sex_1', 'Sex_2', 'family history_0', 'family history_1', 'total categories of antiHTN drugs_0', 'total categories of antiHTN drugs_1', 'total categories of antiHTN drugs_2', 'total categories of antiHTN drugs_3', 'total categories of antiHTN drugs_4']

Factor_96:

['age', 'Body weight(kg)', 'height (cm)', 'hypertension duration (years)', 'Aldosterone (ng/dL)', 'PRA (ngml-1h-1)', 'SBP (mmHg)', 'dBP (mmHg)', 'Na+(\xa0mmol/L)', 'K+(\xa0mmol/L)', 'ever_Hyperthyroidism_0', 'ever_Hyperthyroidism_1', 'ever_COPD_0', 'ever_COPD_1', 'ever diabetics_0', 'ever diabetics_1', 'ever_myocardiac infarction_0', 'ever_myocardiac infarction_1', 'Sex_1', 'Sex_2', 'family history_0', 'family history_1', 'total categories of antiHTN drugs_0', 'total categories of antiHTN drugs_1', 'total categories of antiHTN drugs_2', 'total categories of antiHTN drugs_3', 'total categories of antiHTN drugs_5']

Factor_97:

['age', 'Body weight(kg)', 'height (cm)', 'hypertension duration (years)', 'Aldosterone (ng/dL)', 'PRA (ngml-1h-1)', 'SBP (mmHg)', 'dBP (mmHg)', 'Na+(\xa0mmol/L)', 'K+(\xa0mmol/L)', 'ever_Hyperthyroidism_0', 'ever_Hyperthyroidism_1', 'ever_COPD_0', 'ever_COPD_1', 'ever diabetics_0', 'ever diabetics_1', 'ever_myocardiac infarction_0', 'ever_myocardiac infarction_1', 'Sex_1', 'Sex_2', 'family history_0', 'family history_1', 'total categories of antiHTN drugs_0', 'total categories of antiHTN drugs_1', 'total categories of antiHTN drugs_2', 'total categories of antiHTN drugs_4', 'total categories of antiHTN drugs_5']

Factor_98:

['age', 'Body weight(kg)', 'height (cm)', 'hypertension duration (years)', 'Aldosterone (ng/dL)', 'PRA (ngml-1h-1)', 'SBP (mmHg)', 'dBP (mmHg)', 'Na+(\xa0mmol/L)', 'K+(\xa0mmol/L)', 'ever_Hyperthyroidism_0', 'ever_Hyperthyroidism_1', 'ever_COPD_0', 'ever_COPD_1', 'ever diabetics_0', 'ever diabetics_1', 'ever_myocardiac infarction_0', 'ever_myocardiac infarction_1', 'Sex_1', 'Sex_2', 'family history_0', 'family history_1', 'total categories of antiHTN drugs_0', 'total categories of antiHTN drugs_1', 'total categories of antiHTN drugs_3', 'total categories of antiHTN drugs_4', 'total categories of antiHTN drugs_5']

Factor_99:

['age', 'Body weight(kg)', 'height (cm)', 'hypertension duration (years)', 'Aldosterone (ng/dL)', 'PRA (ngml-1h-1)', 'SBP (mmHg)', 'dBP (mmHg)', 'Na+(\xa0mmol/L)', 'K+(\xa0mmol/L)', 'ever_Hyperthyroidism_0', 'ever_Hyperthyroidism_1', 'ever_COPD_0', 'ever_COPD_1', 'ever diabetics_0', 'ever diabetics_1', 'ever_myocardiac infarction_0', 'ever_myocardiac infarction_1', 'Sex_1', 'Sex_2', 'family history_0', 'family history_1', 'total categories of antiHTN drugs_0', 'total categories of antiHTN drugs_2', 'total categories of antiHTN drugs_3', 'total categories of antiHTN drugs_4', 'total categories of antiHTN drugs_5']

Factor_100:

['age', 'Body weight(kg)', 'height (cm)', 'hypertension duration (years)', 'Aldosterone (ng/dL)', 'PRA (ngml-1h-1)', 'SBP (mmHg)', 'dBP (mmHg)', 'Na+(\xa0mmol/L)', 'K+(\xa0mmol/L)', 'ever_Hyperthyroidism_0', 'ever_Hyperthyroidism_1', 'ever_COPD_0', 'ever_COPD_1', 'ever diabetics_0', 'ever diabetics_1', 'ever_myocardiac infarction_0', 'ever_myocardiac infarction_1', 'Sex_1', 'Sex_2', 'family history_0', 'family history_1', 'total categories of antiHTN drugs_1', 'total categories of antiHTN drugs_2', 'total categories of antiHTN drugs_3', 'total categories of antiHTN drugs_4', 'total categories of antiHTN drugs_5']

Factor_101:

['age', 'Body weight(kg)', 'height (cm)', 'hypertension duration (years)', 'Aldosterone (ng/dL)', 'PRA (ngml-1h-1)', 'SBP (mmHg)', 'dBP (mmHg)', 'Na+(\xa0mmol/L)', 'K+(\xa0mmol/L)', 'ever_Hyperthyroidism_0', 'ever_Hyperthyroidism_1', 'ever_COPD_0', 'ever_COPD_1', 'ever diabetics_0', 'ever diabetics_1', 'ever_myocardiac infarction_0', 'ever_myocardiac infarction_1', 'Sex_1', 'Sex_2', 'family history_0', 'family history_1', 'family history_2', 'total categories of antiHTN drugs_0', 'total categories of antiHTN drugs_1', 'total categories of antiHTN drugs_2', 'total categories of antiHTN drugs_3', 'total categories of antiHTN drugs_4', 'total categories of antiHTN drugs_5']

Factor_102:

['age', 'Body weight(kg)', 'height (cm)', 'hypertension duration (years)', 'Aldosterone (ng/dL)', 'PRA (ngml-1h-1)', 'SBP (mmHg)', 'dBP (mmHg)', 'Na+(\xa0mmol/L)', 'K+(\xa0mmol/L)', 'ever_Hyperthyroidism_0', 'ever_Hyperthyroidism_1', 'ever_COPD_0', 'ever_COPD_1', 'ever diabetics_0', 'ever diabetics_1', 'ever_myocardiac infarction_0', 'ever_myocardiac infarction_1', 'Sex_1', 'Sex_2', 'family history_0', 'family history_1', 'family history_2', 'total categories of antiHTN drugs_0', 'total categories of antiHTN drugs_1', 'total categories of antiHTN drugs_2', 'total categories of antiHTN drugs_3', 'total categories of antiHTN drugs_4']

Factor_103:

['age', 'Body weight(kg)', 'height (cm)', 'hypertension duration (years)', 'Aldosterone (ng/dL)', 'PRA (ngml-1h-1)', 'SBP (mmHg)', 'dBP (mmHg)', 'Na+(\xa0mmol/L)', 'K+(\xa0mmol/L)', 'ever_Hyperthyroidism_0', 'ever_Hyperthyroidism_1', 'ever_COPD_0', 'ever_COPD_1', 'ever diabetics_0', 'ever diabetics_1', 'ever_myocardiac infarction_0', 'ever_myocardiac infarction_1', 'Sex_1', 'Sex_2', 'family history_0', 'family history_1', 'family history_2', 'total categories of antiHTN drugs_0', 'total categories of antiHTN drugs_1', 'total categories of antiHTN drugs_2', 'total categories of antiHTN drugs_3', 'total categories of antiHTN drugs_5']

Factor_104:

['age', 'Body weight(kg)', 'height (cm)', 'hypertension duration (years)', 'Aldosterone (ng/dL)', 'PRA (ngml-1h-1)', 'SBP (mmHg)', 'dBP (mmHg)', 'Na+(\xa0mmol/L)', 'K+(\xa0mmol/L)', 'ever_Hyperthyroidism_0', 'ever_Hyperthyroidism_1', 'ever_COPD_0', 'ever_COPD_1', 'ever diabetics_0', 'ever diabetics_1', 'ever_myocardiac infarction_0', 'ever_myocardiac infarction_1', 'Sex_1', 'Sex_2', 'family history_0', 'family history_1', 'family history_2', 'total categories of antiHTN drugs_0', 'total categories of antiHTN drugs_1', 'total categories of antiHTN drugs_2', 'total categories of antiHTN drugs_4', 'total categories of antiHTN drugs_5']

Factor_105:

['age', 'Body weight(kg)', 'height (cm)', 'hypertension duration (years)', 'Aldosterone (ng/dL)', 'PRA (ngml-1h-1)', 'SBP (mmHg)', 'dBP (mmHg)', 'Na+(\xa0mmol/L)', 'K+(\xa0mmol/L)', 'ever_Hyperthyroidism_0', 'ever_Hyperthyroidism_1', 'ever_COPD_0', 'ever_COPD_1', 'ever diabetics_0', 'ever diabetics_1', 'ever_myocardiac infarction_0', 'ever_myocardiac infarction_1', 'Sex_1', 'Sex_2', 'family history_0', 'family history_1', 'family history_2', 'total categories of antiHTN drugs_0', 'total categories of antiHTN drugs_2', 'total categories of antiHTN drugs_3', 'total categories of antiHTN drugs_4', 'total categories of antiHTN drugs_5']

Factor_106:

['age', 'Body weight(kg)', 'height (cm)', 'hypertension duration (years)', 'Aldosterone (ng/dL)', 'PRA (ngml-1h-1)', 'SBP (mmHg)', 'dBP (mmHg)', 'Na+(\xa0mmol/L)', 'K+(\xa0mmol/L)', 'ever_Hyperthyroidism_0', 'ever_Hyperthyroidism_1', 'ever_COPD_0', 'ever_COPD_1', 'ever diabetics_0', 'ever diabetics_1', 'ever_myocardiac infarction_0', 'ever_myocardiac infarction_1', 'Sex_1', 'Sex_2', 'family history_0', 'family history_1', 'family history_2', 'total categories of antiHTN drugs_1', 'total categories of antiHTN drugs_2', 'total categories of antiHTN drugs_3', 'total categories of antiHTN drugs_4', 'total categories of antiHTN drugs_5']

Factor_107:

['age', 'Body weight(kg)', 'height (cm)', 'hypertension duration (years)', 'Aldosterone (ng/dL)', 'PRA (ngml-1h-1)', 'SBP (mmHg)', 'dBP (mmHg)', 'Na+(\xa0mmol/L)', 'K+(\xa0mmol/L)', 'ever_Hyperthyroidism_0', 'ever_Hyperthyroidism_1', 'ever_COPD_0', 'ever_COPD_1', 'ever diabetics_0', 'ever diabetics_1', 'ever_myocardiac infarction_0', 'ever_myocardiac infarction_1', 'Sex_1', 'Sex_2', 'family history_0', 'family history_1', 'total categories of antiHTN drugs_0', 'total categories of antiHTN drugs_1', 'total categories of antiHTN drugs_2', 'total categories of antiHTN drugs_3', 'total categories of antiHTN drugs_4', 'total categories of antiHTN drugs_5']

Factor_108:

['age', 'Body weight(kg)', 'height (cm)', 'hypertension duration (years)', 'Aldosterone (ng/dL)', 'PRA (ngml-1h-1)', 'SBP (mmHg)', 'dBP (mmHg)', 'Na+(\xa0mmol/L)', 'K+(\xa0mmol/L)', 'ever_Hyperthyroidism_0', 'ever_Hyperthyroidism_1', 'ever_COPD_0', 'ever_COPD_1', 'ever diabetics_0', 'ever diabetics_1', 'ever_myocardiac infarction_0', 'ever_myocardiac infarction_1', 'Sex_1', 'Sex_2', 'family history_1', 'family history_2', 'total categories of antiHTN drugs_0', 'total categories of antiHTN drugs_1', 'total categories of antiHTN drugs_2', 'total categories of antiHTN drugs_3', 'total categories of antiHTN drugs_4', 'total categories of antiHTN drugs_5']

Factor_109:

['age', 'Body weight(kg)', 'height (cm)', 'hypertension duration (years)', 'Aldosterone (ng/dL)', 'PRA (ngml-1h-1)', 'SBP (mmHg)', 'dBP (mmHg)', 'Na+(\xa0mmol/L)', 'K+(\xa0mmol/L)', 'ever_Hyperthyroidism_0', 'ever_Hyperthyroidism_1', 'ever_COPD_0', 'ever_COPD_1', 'ever diabetics_0', 'ever diabetics_1', 'ever_myocardiac infarction_0', 'ever_myocardiac infarction_1', 'Sex_1', 'family history_0', 'family history_1', 'family history_2', 'total categories of antiHTN drugs_0', 'total categories of antiHTN drugs_1', 'total categories of antiHTN drugs_2', 'total categories of antiHTN drugs_3', 'total categories of antiHTN drugs_4', 'total categories of antiHTN drugs_5']

Factor_110:

['age', 'Body weight(kg)', 'height (cm)', 'hypertension duration (years)', 'Aldosterone (ng/dL)', 'PRA (ngml-1h-1)', 'SBP (mmHg)', 'dBP (mmHg)', 'Na+(\xa0mmol/L)', 'K+(\xa0mmol/L)', 'ever_Hyperthyroidism_0', 'ever_Hyperthyroidism_1', 'ever_COPD_0', 'ever_COPD_1', 'ever diabetics_0', 'ever diabetics_1', 'ever_myocardiac infarction_0', 'ever_myocardiac infarction_1', 'Sex_2', 'family history_0', 'family history_1', 'family history_2', 'total categories of antiHTN drugs_0', 'total categories of antiHTN drugs_1', 'total categories of antiHTN drugs_2', 'total categories of antiHTN drugs_3', 'total categories of antiHTN drugs_4', 'total categories of antiHTN drugs_5']

Factor_111:

['age', 'Body weight(kg)', 'height (cm)', 'hypertension duration (years)', 'Aldosterone (ng/dL)', 'PRA (ngml-1h-1)', 'SBP (mmHg)', 'dBP (mmHg)', 'Na+(\xa0mmol/L)', 'K+(\xa0mmol/L)', 'ever_Hyperthyroidism_0', 'ever_Hyperthyroidism_1', 'ever_COPD_0', 'ever_COPD_1', 'ever diabetics_0', 'ever_myocardiac infarction_0', 'ever_myocardiac infarction_1', 'Sex_1', 'Sex_2', 'family history_0', 'family history_1', 'family history_2', 'total categories of antiHTN drugs_0', 'total categories of antiHTN drugs_1', 'total categories of antiHTN drugs_2', 'total categories of antiHTN drugs_3', 'total categories of antiHTN drugs_4', 'total categories of antiHTN drugs_5']

Factor_112:

['age', 'Body weight(kg)', 'height (cm)', 'hypertension duration (years)', 'Aldosterone (ng/dL)', 'PRA (ngml-1h-1)', 'SBP (mmHg)', 'dBP (mmHg)', 'Na+(\xa0mmol/L)', 'K+(\xa0mmol/L)', 'ever_Hyperthyroidism_0', 'ever_Hyperthyroidism_1', 'ever_COPD_0', 'ever_COPD_1', 'ever diabetics_1', 'ever_myocardiac infarction_0', 'ever_myocardiac infarction_1', 'Sex_1', 'Sex_2', 'family history_0', 'family history_1', 'family history_2', 'total categories of antiHTN drugs_0', 'total categories of antiHTN drugs_1', 'total categories of antiHTN drugs_2', 'total categories of antiHTN drugs_3', 'total categories of antiHTN drugs_4', 'total categories of antiHTN drugs_5']

Factor_113:

['age', 'Body weight(kg)', 'height (cm)', 'hypertension duration (years)', 'Aldosterone (ng/dL)', 'PRA (ngml-1h-1)', 'SBP (mmHg)', 'dBP (mmHg)', 'Na+(\xa0mmol/L)', 'K+(\xa0mmol/L)', 'ever_Hyperthyroidism_0', 'ever_Hyperthyroidism_1', 'ever_COPD_0', 'ever diabetics_0', 'ever diabetics_1', 'ever_myocardiac infarction_0', 'ever_myocardiac infarction_1', 'Sex_1', 'Sex_2', 'family history_0', 'family history_1', 'family history_2', 'total categories of antiHTN drugs_0', 'total categories of antiHTN drugs_1', 'total categories of antiHTN drugs_2', 'total categories of antiHTN drugs_3', 'total categories of antiHTN drugs_4', 'total categories of antiHTN drugs_5']

Factor_114:

['age', 'Body weight(kg)', 'height (cm)', 'hypertension duration (years)', 'Aldosterone (ng/dL)', 'PRA (ngml-1h-1)', 'SBP (mmHg)', 'dBP (mmHg)', 'Na+(\xa0mmol/L)', 'K+(\xa0mmol/L)', 'ever_Hyperthyroidism_0', 'ever_Hyperthyroidism_1', 'ever_COPD_1', 'ever diabetics_0', 'ever diabetics_1', 'ever_myocardiac infarction_0', 'ever_myocardiac infarction_1', 'Sex_1', 'Sex_2', 'family history_0', 'family history_1', 'family history_2', 'total categories of antiHTN drugs_0', 'total categories of antiHTN drugs_1', 'total categories of antiHTN drugs_2', 'total categories of antiHTN drugs_3', 'total categories of antiHTN drugs_4', 'total categories of antiHTN drugs_5']

Factor_115:

['age', 'Body weight(kg)', 'height (cm)', 'hypertension duration (years)', 'Aldosterone (ng/dL)', 'PRA (ngml-1h-1)', 'SBP (mmHg)', 'dBP (mmHg)', 'Na+(\xa0mmol/L)', 'K+(\xa0mmol/L)', 'ever_Hyperthyroidism_0', 'ever_COPD_0', 'ever_COPD_1', 'ever diabetics_0', 'ever diabetics_1', 'ever_myocardiac infarction_0', 'ever_myocardiac infarction_1', 'Sex_1', 'Sex_2', 'family history_0', 'family history_1', 'family history_2', 'total categories of antiHTN drugs_0', 'total categories of antiHTN drugs_1', 'total categories of antiHTN drugs_2', 'total categories of antiHTN drugs_3', 'total categories of antiHTN drugs_4', 'total categories of antiHTN drugs_5']

Factor_116:

['age', 'Body weight(kg)', 'height (cm)', 'hypertension duration (years)', 'Aldosterone (ng/dL)', 'PRA (ngml-1h-1)', 'SBP (mmHg)', 'dBP (mmHg)', 'Na+(\xa0mmol/L)', 'ever_Hyperthyroidism_0', 'ever_Hyperthyroidism_1', 'ever_COPD_0', 'ever_COPD_1', 'ever diabetics_0', 'ever diabetics_1', 'ever_myocardiac infarction_0', 'ever_myocardiac infarction_1', 'Sex_1', 'Sex_2', 'family history_0', 'family history_1', 'family history_2', 'total categories of antiHTN drugs_0', 'total categories of antiHTN drugs_1', 'total categories of antiHTN drugs_2', 'total categories of antiHTN drugs_3', 'total categories of antiHTN drugs_4', 'total categories of antiHTN drugs_5']

Factor_117:

['age', 'Body weight(kg)', 'height (cm)', 'hypertension duration (years)', 'Aldosterone (ng/dL)', 'PRA (ngml-1h-1)', 'SBP (mmHg)', 'dBP (mmHg)', 'K+(\xa0mmol/L)', 'ever_Hyperthyroidism_0', 'ever_Hyperthyroidism_1', 'ever_COPD_0', 'ever_COPD_1', 'ever diabetics_0', 'ever diabetics_1', 'ever_myocardiac infarction_0', 'ever_myocardiac infarction_1', 'Sex_1', 'Sex_2', 'family history_0', 'family history_1', 'family history_2', 'total categories of antiHTN drugs_0', 'total categories of antiHTN drugs_1', 'total categories of antiHTN drugs_2', 'total categories of antiHTN drugs_3', 'total categories of antiHTN drugs_4', 'total categories of antiHTN drugs_5']

Factor_118:

['age', 'Body weight(kg)', 'height (cm)', 'hypertension duration (years)', 'Aldosterone (ng/dL)', 'PRA (ngml-1h-1)', 'SBP (mmHg)', 'Na+(\xa0mmol/L)', 'K+(\xa0mmol/L)', 'ever_Hyperthyroidism_0', 'ever_Hyperthyroidism_1', 'ever_COPD_0', 'ever_COPD_1', 'ever diabetics_0', 'ever diabetics_1', 'ever_myocardiac infarction_0', 'ever_myocardiac infarction_1', 'Sex_1', 'Sex_2', 'family history_0', 'family history_1', 'family history_2', 'total categories of antiHTN drugs_0', 'total categories of antiHTN drugs_1', 'total categories of antiHTN drugs_2', 'total categories of antiHTN drugs_3', 'total categories of antiHTN drugs_4', 'total categories of antiHTN drugs_5']

Factor_119:

['age', 'Body weight(kg)', 'height (cm)', 'hypertension duration (years)', 'Aldosterone (ng/dL)', 'PRA (ngml-1h-1)', 'dBP (mmHg)', 'Na+(\xa0mmol/L)', 'K+(\xa0mmol/L)', 'ever_Hyperthyroidism_0', 'ever_Hyperthyroidism_1', 'ever_COPD_0', 'ever_COPD_1', 'ever diabetics_0', 'ever diabetics_1', 'ever_myocardiac infarction_0', 'ever_myocardiac infarction_1', 'Sex_1', 'Sex_2', 'family history_0', 'family history_1', 'family history_2', 'total categories of antiHTN drugs_0', 'total categories of antiHTN drugs_1', 'total categories of antiHTN drugs_2', 'total categories of antiHTN drugs_3', 'total categories of antiHTN drugs_4', 'total categories of antiHTN drugs_5']

Factor_120:

['age', 'Body weight(kg)', 'height (cm)', 'hypertension duration (years)', 'Aldosterone (ng/dL)', 'SBP (mmHg)', 'dBP (mmHg)', 'Na+(\xa0mmol/L)', 'K+(\xa0mmol/L)', 'ever_Hyperthyroidism_0', 'ever_Hyperthyroidism_1', 'ever_COPD_0', 'ever_COPD_1', 'ever diabetics_0', 'ever diabetics_1', 'ever_myocardiac infarction_0', 'ever_myocardiac infarction_1', 'Sex_1', 'Sex_2', 'family history_0', 'family history_1', 'family history_2', 'total categories of antiHTN drugs_0', 'total categories of antiHTN drugs_1', 'total categories of antiHTN drugs_2', 'total categories of antiHTN drugs_3', 'total categories of antiHTN drugs_4', 'total categories of antiHTN drugs_5']

Factor_121:

['age', 'Body weight(kg)', 'height (cm)', 'hypertension duration (years)', 'PRA (ngml-1h-1)', 'SBP (mmHg)', 'dBP (mmHg)', 'Na+(\xa0mmol/L)', 'K+(\xa0mmol/L)', 'ever_Hyperthyroidism_0', 'ever_Hyperthyroidism_1', 'ever_COPD_0', 'ever_COPD_1', 'ever diabetics_0', 'ever diabetics_1', 'ever_myocardiac infarction_0', 'ever_myocardiac infarction_1', 'Sex_1', 'Sex_2', 'family history_0', 'family history_1', 'family history_2', 'total categories of antiHTN drugs_0', 'total categories of antiHTN drugs_1', 'total categories of antiHTN drugs_2', 'total categories of antiHTN drugs_3', 'total categories of antiHTN drugs_4', 'total categories of antiHTN drugs_5']

Factor_122:

['age', 'Body weight(kg)', 'height (cm)', 'Aldosterone (ng/dL)', 'PRA (ngml-1h-1)', 'SBP (mmHg)', 'dBP (mmHg)', 'Na+(\xa0mmol/L)', 'K+(\xa0mmol/L)', 'ever_Hyperthyroidism_0', 'ever_Hyperthyroidism_1', 'ever_COPD_0', 'ever_COPD_1', 'ever diabetics_0', 'ever diabetics_1', 'ever_myocardiac infarction_0', 'ever_myocardiac infarction_1', 'Sex_1', 'Sex_2', 'family history_0', 'family history_1', 'family history_2', 'total categories of antiHTN drugs_0', 'total categories of antiHTN drugs_1', 'total categories of antiHTN drugs_2', 'total categories of antiHTN drugs_3', 'total categories of antiHTN drugs_4', 'total categories of antiHTN drugs_5']

Factor_123:

['age', 'Body weight(kg)', 'hypertension duration (years)', 'Aldosterone (ng/dL)', 'PRA (ngml-1h-1)', 'SBP (mmHg)', 'dBP (mmHg)', 'Na+(\xa0mmol/L)', 'K+(\xa0mmol/L)', 'ever_Hyperthyroidism_0', 'ever_Hyperthyroidism_1', 'ever_COPD_0', 'ever_COPD_1', 'ever diabetics_0', 'ever diabetics_1', 'ever_myocardiac infarction_0', 'ever_myocardiac infarction_1', 'Sex_1', 'Sex_2', 'family history_0', 'family history_1', 'family history_2', 'total categories of antiHTN drugs_0', 'total categories of antiHTN drugs_1', 'total categories of antiHTN drugs_2', 'total categories of antiHTN drugs_3', 'total categories of antiHTN drugs_4', 'total categories of antiHTN drugs_5']

Factor_124:

['Body weight(kg)', 'height (cm)', 'hypertension duration (years)', 'Aldosterone (ng/dL)', 'PRA (ngml-1h-1)', 'SBP (mmHg)', 'dBP (mmHg)', 'Na+(\xa0mmol/L)', 'K+(\xa0mmol/L)', 'ever_Hyperthyroidism_0', 'ever_Hyperthyroidism_1', 'ever_COPD_0', 'ever_COPD_1', 'ever diabetics_0', 'ever diabetics_1', 'ever_myocardiac infarction_0', 'ever_myocardiac infarction_1', 'Sex_1', 'Sex_2', 'family history_0', 'family history_1', 'family history_2', 'total categories of antiHTN drugs_0', 'total categories of antiHTN drugs_1', 'total categories of antiHTN drugs_2', 'total categories of antiHTN drugs_3', 'total categories of antiHTN drugs_4', 'total categories of antiHTN drugs_5']

Factor_125:

['age', 'Body weight(kg)', 'height (cm)', 'hypertension duration (years)', 'Aldosterone (ng/dL)', 'PRA (ngml-1h-1)', 'SBP (mmHg)', 'dBP (mmHg)', 'Na+(\xa0mmol/L)', 'K+(\xa0mmol/L)', 'ever_Hyperthyroidism_0', 'ever_Hyperthyroidism_1', 'ever_COPD_0', 'ever_COPD_1', 'ever diabetics_0', 'ever diabetics_1', 'ever_myocardiac infarction_0', 'ever_myocardiac infarction_1', 'Sex_1', 'Sex_2', 'family history_0', 'family history_1', 'family history_2', 'total categories of antiHTN drugs_0', 'total categories of antiHTN drugs_1', 'total categories of antiHTN drugs_2', 'total categories of antiHTN drugs_3']

Factor_126:

['age', 'Body weight(kg)', 'height (cm)', 'hypertension duration (years)', 'Aldosterone (ng/dL)', 'PRA (ngml-1h-1)', 'SBP (mmHg)', 'dBP (mmHg)', 'Na+(\xa0mmol/L)', 'K+(\xa0mmol/L)', 'ever_Hyperthyroidism_0', 'ever_Hyperthyroidism_1', 'ever_COPD_0', 'ever_COPD_1', 'ever diabetics_0', 'ever diabetics_1', 'ever_myocardiac infarction_0', 'ever_myocardiac infarction_1', 'Sex_1', 'Sex_2', 'family history_0', 'family history_1', 'family history_2', 'total categories of antiHTN drugs_0', 'total categories of antiHTN drugs_1', 'total categories of antiHTN drugs_2', 'total categories of antiHTN drugs_4']

Factor_127:

['age', 'Body weight(kg)', 'height (cm)', 'hypertension duration (years)', 'Aldosterone (ng/dL)', 'PRA (ngml-1h-1)', 'SBP (mmHg)', 'dBP (mmHg)', 'Na+(\xa0mmol/L)', 'K+(\xa0mmol/L)', 'ever_Hyperthyroidism_0', 'ever_Hyperthyroidism_1', 'ever_COPD_0', 'ever_COPD_1', 'ever diabetics_0', 'ever diabetics_1', 'ever_myocardiac infarction_0', 'ever_myocardiac infarction_1', 'Sex_1', 'Sex_2', 'family history_0', 'family history_1', 'family history_2', 'total categories of antiHTN drugs_0', 'total categories of antiHTN drugs_1', 'total categories of antiHTN drugs_2', 'total categories of antiHTN drugs_5']

Factor_128:

['age', 'Body weight(kg)', 'height (cm)', 'hypertension duration (years)', 'Aldosterone (ng/dL)', 'PRA (ngml-1h-1)', 'SBP (mmHg)', 'dBP (mmHg)', 'Na+(\xa0mmol/L)', 'K+(\xa0mmol/L)', 'ever_Hyperthyroidism_0', 'ever_Hyperthyroidism_1', 'ever_COPD_0', 'ever_COPD_1', 'ever diabetics_0', 'ever diabetics_1', 'ever_myocardiac infarction_0', 'ever_myocardiac infarction_1', 'Sex_1', 'Sex_2', 'family history_0', 'family history_1', 'family history_2', 'total categories of antiHTN drugs_0', 'total categories of antiHTN drugs_1', 'total categories of antiHTN drugs_3', 'total categories of antiHTN drugs_4']

Factor_129:

['age', 'Body weight(kg)', 'height (cm)', 'hypertension duration (years)', 'Aldosterone (ng/dL)', 'PRA (ngml-1h-1)', 'SBP (mmHg)', 'dBP (mmHg)', 'Na+(\xa0mmol/L)', 'K+(\xa0mmol/L)', 'ever_Hyperthyroidism_0', 'ever_Hyperthyroidism_1', 'ever_COPD_0', 'ever_COPD_1', 'ever diabetics_0', 'ever diabetics_1', 'ever_myocardiac infarction_0', 'ever_myocardiac infarction_1', 'Sex_1', 'Sex_2', 'family history_0', 'family history_1', 'family history_2', 'total categories of antiHTN drugs_0', 'total categories of antiHTN drugs_1', 'total categories of antiHTN drugs_3', 'total categories of antiHTN drugs_5']

Factor_130:

['age', 'Body weight(kg)', 'height (cm)', 'hypertension duration (years)', 'Aldosterone (ng/dL)', 'PRA (ngml-1h-1)', 'SBP (mmHg)', 'dBP (mmHg)', 'Na+(\xa0mmol/L)', 'K+(\xa0mmol/L)', 'ever_Hyperthyroidism_0', 'ever_Hyperthyroidism_1', 'ever_COPD_0', 'ever_COPD_1', 'ever diabetics_0', 'ever diabetics_1', 'ever_myocardiac infarction_0', 'ever_myocardiac infarction_1', 'Sex_1', 'Sex_2', 'family history_0', 'family history_1', 'family history_2', 'total categories of antiHTN drugs_0', 'total categories of antiHTN drugs_2', 'total categories of antiHTN drugs_3', 'total categories of antiHTN drugs_4']

Factor_131:

['age', 'Body weight(kg)', 'height (cm)', 'hypertension duration (years)', 'Aldosterone (ng/dL)', 'PRA (ngml-1h-1)', 'SBP (mmHg)', 'dBP (mmHg)', 'Na+(\xa0mmol/L)', 'K+(\xa0mmol/L)', 'ever_Hyperthyroidism_0', 'ever_Hyperthyroidism_1', 'ever_COPD_0', 'ever_COPD_1', 'ever diabetics_0', 'ever diabetics_1', 'ever_myocardiac infarction_0', 'ever_myocardiac infarction_1', 'Sex_1', 'Sex_2', 'family history_0', 'family history_1', 'family history_2', 'total categories of antiHTN drugs_0', 'total categories of antiHTN drugs_2', 'total categories of antiHTN drugs_4', 'total categories of antiHTN drugs_5']

Factor_132:

['age', 'Body weight(kg)', 'height (cm)', 'hypertension duration (years)', 'Aldosterone (ng/dL)', 'PRA (ngml-1h-1)', 'SBP (mmHg)', 'dBP (mmHg)', 'Na+(\xa0mmol/L)', 'K+(\xa0mmol/L)', 'ever_Hyperthyroidism_0', 'ever_Hyperthyroidism_1', 'ever_COPD_0', 'ever_COPD_1', 'ever diabetics_0', 'ever diabetics_1', 'ever_myocardiac infarction_0', 'ever_myocardiac infarction_1', 'Sex_1', 'Sex_2', 'family history_0', 'family history_1', 'family history_2', 'total categories of antiHTN drugs_0', 'total categories of antiHTN drugs_3', 'total categories of antiHTN drugs_4', 'total categories of antiHTN drugs_5']

Factor_133:

['age', 'Body weight(kg)', 'height (cm)', 'hypertension duration (years)', 'Aldosterone (ng/dL)', 'PRA (ngml-1h-1)', 'SBP (mmHg)', 'dBP (mmHg)', 'Na+(\xa0mmol/L)', 'K+(\xa0mmol/L)', 'ever_Hyperthyroidism_0', 'ever_Hyperthyroidism_1', 'ever_COPD_0', 'ever_COPD_1', 'ever diabetics_0', 'ever diabetics_1', 'ever_myocardiac infarction_0', 'ever_myocardiac infarction_1', 'Sex_1', 'Sex_2', 'family history_0', 'family history_1', 'family history_2', 'total categories of antiHTN drugs_1', 'total categories of antiHTN drugs_2', 'total categories of antiHTN drugs_3', 'total categories of antiHTN drugs_4']

Factor_134:

['age', 'Body weight(kg)', 'height (cm)', 'hypertension duration (years)', 'Aldosterone (ng/dL)', 'PRA (ngml-1h-1)', 'SBP (mmHg)', 'dBP (mmHg)', 'Na+(\xa0mmol/L)', 'K+(\xa0mmol/L)', 'ever_Hyperthyroidism_0', 'ever_Hyperthyroidism_1', 'ever_COPD_0', 'ever_COPD_1', 'ever diabetics_0', 'ever diabetics_1', 'ever_myocardiac infarction_0', 'ever_myocardiac infarction_1', 'Sex_1', 'Sex_2', 'family history_0', 'family history_1', 'family history_2', 'total categories of antiHTN drugs_1', 'total categories of antiHTN drugs_2', 'total categories of antiHTN drugs_3', 'total categories of antiHTN drugs_5']

Factor_135:

['age', 'Body weight(kg)', 'height (cm)', 'hypertension duration (years)', 'Aldosterone (ng/dL)', 'PRA (ngml-1h-1)', 'SBP (mmHg)', 'dBP (mmHg)', 'Na+(\xa0mmol/L)', 'K+(\xa0mmol/L)', 'ever_Hyperthyroidism_0', 'ever_Hyperthyroidism_1', 'ever_COPD_0', 'ever_COPD_1', 'ever diabetics_0', 'ever diabetics_1', 'ever_myocardiac infarction_0', 'ever_myocardiac infarction_1', 'Sex_1', 'Sex_2', 'family history_0', 'family history_1', 'family history_2', 'total categories of antiHTN drugs_1', 'total categories of antiHTN drugs_2', 'total categories of antiHTN drugs_4', 'total categories of antiHTN drugs_5']

Factor_136:

['age', 'Body weight(kg)', 'height (cm)', 'hypertension duration (years)', 'Aldosterone (ng/dL)', 'PRA (ngml-1h-1)', 'SBP (mmHg)', 'dBP (mmHg)', 'Na+(\xa0mmol/L)', 'K+(\xa0mmol/L)', 'ever_Hyperthyroidism_0', 'ever_Hyperthyroidism_1', 'ever_COPD_0', 'ever_COPD_1', 'ever diabetics_0', 'ever diabetics_1', 'ever_myocardiac infarction_0', 'ever_myocardiac infarction_1', 'Sex_1', 'Sex_2', 'family history_0', 'family history_1', 'family history_2', 'total categories of antiHTN drugs_1', 'total categories of antiHTN drugs_3', 'total categories of antiHTN drugs_4', 'total categories of antiHTN drugs_5']

Factor_137:

['age', 'Body weight(kg)', 'height (cm)', 'hypertension duration (years)', 'Aldosterone (ng/dL)', 'PRA (ngml-1h-1)', 'SBP (mmHg)', 'dBP (mmHg)', 'Na+(\xa0mmol/L)', 'K+(\xa0mmol/L)', 'ever_Hyperthyroidism_0', 'ever_Hyperthyroidism_1', 'ever_COPD_0', 'ever_COPD_1', 'ever diabetics_0', 'ever diabetics_1', 'ever_myocardiac infarction_0', 'ever_myocardiac infarction_1', 'Sex_1', 'Sex_2', 'family history_0', 'family history_1', 'family history_2', 'total categories of antiHTN drugs_2', 'total categories of antiHTN drugs_3', 'total categories of antiHTN drugs_4', 'total categories of antiHTN drugs_5']

Factor_138:

['age', 'Body weight(kg)', 'height (cm)', 'hypertension duration (years)', 'Aldosterone (ng/dL)', 'PRA (ngml-1h-1)', 'SBP (mmHg)', 'dBP (mmHg)', 'Na+(\xa0mmol/L)', 'K+(\xa0mmol/L)', 'ever_Hyperthyroidism_0', 'ever_Hyperthyroidism_1', 'ever_COPD_0', 'ever_COPD_1', 'ever diabetics_0', 'ever diabetics_1', 'ever_myocardiac infarction_0', 'ever_myocardiac infarction_1', 'Sex_1', 'Sex_2', 'family history_0', 'family history_1', 'total categories of antiHTN drugs_0', 'total categories of antiHTN drugs_1', 'total categories of antiHTN drugs_2', 'total categories of antiHTN drugs_3', 'total categories of antiHTN drugs_4']

Factor_139:

['age', 'Body weight(kg)', 'height (cm)', 'hypertension duration (years)', 'Aldosterone (ng/dL)', 'PRA (ngml-1h-1)', 'SBP (mmHg)', 'dBP (mmHg)', 'Na+(\xa0mmol/L)', 'K+(\xa0mmol/L)', 'ever_Hyperthyroidism_0', 'ever_Hyperthyroidism_1', 'ever_COPD_0', 'ever_COPD_1', 'ever diabetics_0', 'ever diabetics_1', 'ever_myocardiac infarction_0', 'ever_myocardiac infarction_1', 'Sex_1', 'Sex_2', 'family history_0', 'family history_1', 'total categories of antiHTN drugs_0', 'total categories of antiHTN drugs_1', 'total categories of antiHTN drugs_2', 'total categories of antiHTN drugs_3', 'total categories of antiHTN drugs_5']

Factor_140:

['age', 'Body weight(kg)', 'height (cm)', 'hypertension duration (years)', 'Aldosterone (ng/dL)', 'PRA (ngml-1h-1)', 'SBP (mmHg)', 'dBP (mmHg)', 'Na+(\xa0mmol/L)', 'K+(\xa0mmol/L)', 'ever_Hyperthyroidism_0', 'ever_Hyperthyroidism_1', 'ever_COPD_0', 'ever_COPD_1', 'ever diabetics_0', 'ever diabetics_1', 'ever_myocardiac infarction_0', 'ever_myocardiac infarction_1', 'Sex_1', 'Sex_2', 'family history_0', 'family history_1', 'total categories of antiHTN drugs_0', 'total categories of antiHTN drugs_1', 'total categories of antiHTN drugs_2', 'total categories of antiHTN drugs_4', 'total categories of antiHTN drugs_5']

Factor_141:

['age', 'Body weight(kg)', 'height (cm)', 'hypertension duration (years)', 'Aldosterone (ng/dL)', 'PRA (ngml-1h-1)', 'SBP (mmHg)', 'dBP (mmHg)', 'Na+(\xa0mmol/L)', 'K+(\xa0mmol/L)', 'ever_Hyperthyroidism_0', 'ever_Hyperthyroidism_1', 'ever_COPD_0', 'ever_COPD_1', 'ever diabetics_0', 'ever diabetics_1', 'ever_myocardiac infarction_0', 'ever_myocardiac infarction_1', 'Sex_1', 'Sex_2', 'family history_0', 'family history_1', 'total categories of antiHTN drugs_0', 'total categories of antiHTN drugs_1', 'total categories of antiHTN drugs_3', 'total categories of antiHTN drugs_4', 'total categories of antiHTN drugs_5']

Factor_142:

['age', 'Body weight(kg)', 'height (cm)', 'hypertension duration (years)', 'Aldosterone (ng/dL)', 'PRA (ngml-1h-1)', 'SBP (mmHg)', 'dBP (mmHg)', 'Na+(\xa0mmol/L)', 'K+(\xa0mmol/L)', 'ever_Hyperthyroidism_0', 'ever_Hyperthyroidism_1', 'ever_COPD_0', 'ever_COPD_1', 'ever diabetics_0', 'ever diabetics_1', 'ever_myocardiac infarction_0', 'ever_myocardiac infarction_1', 'Sex_1', 'Sex_2', 'family history_0', 'family history_1', 'total categories of antiHTN drugs_0', 'total categories of antiHTN drugs_2', 'total categories of antiHTN drugs_3', 'total categories of antiHTN drugs_4', 'total categories of antiHTN drugs_5']

Factor_143:

['age', 'Body weight(kg)', 'height (cm)', 'hypertension duration (years)', 'Aldosterone (ng/dL)', 'PRA (ngml-1h-1)', 'SBP (mmHg)', 'dBP (mmHg)', 'Na+(\xa0mmol/L)', 'K+(\xa0mmol/L)', 'ever_Hyperthyroidism_0', 'ever_Hyperthyroidism_1', 'ever_COPD_0', 'ever_COPD_1', 'ever diabetics_0', 'ever diabetics_1', 'ever_myocardiac infarction_0', 'ever_myocardiac infarction_1', 'Sex_1', 'Sex_2', 'family history_0', 'family history_2', 'total categories of antiHTN drugs_0', 'total categories of antiHTN drugs_1', 'total categories of antiHTN drugs_2', 'total categories of antiHTN drugs_3', 'total categories of antiHTN drugs_5']

Factor_144:

['age', 'Body weight(kg)', 'height (cm)', 'hypertension duration (years)', 'Aldosterone (ng/dL)', 'PRA (ngml-1h-1)', 'SBP (mmHg)', 'dBP (mmHg)', 'Na+(\xa0mmol/L)', 'K+(\xa0mmol/L)', 'ever_Hyperthyroidism_0', 'ever_Hyperthyroidism_1', 'ever_COPD_0', 'ever_COPD_1', 'ever diabetics_0', 'ever diabetics_1', 'ever_myocardiac infarction_0', 'ever_myocardiac infarction_1', 'Sex_1', 'Sex_2', 'family history_0', 'family history_2', 'total categories of antiHTN drugs_0', 'total categories of antiHTN drugs_1', 'total categories of antiHTN drugs_2', 'total categories of antiHTN drugs_4', 'total categories of antiHTN drugs_5']

Factor_145:

['age', 'Body weight(kg)', 'height (cm)', 'hypertension duration (years)', 'Aldosterone (ng/dL)', 'PRA (ngml-1h-1)', 'SBP (mmHg)', 'dBP (mmHg)', 'Na+(\xa0mmol/L)', 'K+(\xa0mmol/L)', 'ever_Hyperthyroidism_0', 'ever_Hyperthyroidism_1', 'ever_COPD_0', 'ever_COPD_1', 'ever diabetics_0', 'ever diabetics_1', 'ever_myocardiac infarction_0', 'ever_myocardiac infarction_1', 'Sex_1', 'Sex_2', 'family history_0', 'family history_2', 'total categories of antiHTN drugs_0', 'total categories of antiHTN drugs_2', 'total categories of antiHTN drugs_3', 'total categories of antiHTN drugs_4', 'total categories of antiHTN drugs_5']

Factor_146:

['age', 'Body weight(kg)', 'height (cm)', 'hypertension duration (years)', 'Aldosterone (ng/dL)', 'PRA (ngml-1h-1)', 'SBP (mmHg)', 'dBP (mmHg)', 'Na+(\xa0mmol/L)', 'K+(\xa0mmol/L)', 'ever_Hyperthyroidism_0', 'ever_Hyperthyroidism_1', 'ever_COPD_0', 'ever_COPD_1', 'ever diabetics_0', 'ever diabetics_1', 'ever_myocardiac infarction_0', 'ever_myocardiac infarction_1', 'Sex_1', 'Sex_2', 'family history_0', 'family history_2', 'total categories of antiHTN drugs_1', 'total categories of antiHTN drugs_2', 'total categories of antiHTN drugs_3', 'total categories of antiHTN drugs_4', 'total categories of antiHTN drugs_5']

Factor_147:

['age', 'Body weight(kg)', 'height (cm)', 'hypertension duration (years)', 'Aldosterone (ng/dL)', 'PRA (ngml-1h-1)', 'SBP (mmHg)', 'dBP (mmHg)', 'Na+(\xa0mmol/L)', 'K+(\xa0mmol/L)', 'ever_Hyperthyroidism_0', 'ever_Hyperthyroidism_1', 'ever_COPD_0', 'ever_COPD_1', 'ever diabetics_0', 'ever diabetics_1', 'ever_myocardiac infarction_0', 'ever_myocardiac infarction_1', 'Sex_1', 'Sex_2', 'family history_0', 'total categories of antiHTN drugs_0', 'total categories of antiHTN drugs_1', 'total categories of antiHTN drugs_2', 'total categories of antiHTN drugs_3', 'total categories of antiHTN drugs_4', 'total categories of antiHTN drugs_5']

Factor_148:

['age', 'Body weight(kg)', 'height (cm)', 'hypertension duration (years)', 'Aldosterone (ng/dL)', 'PRA (ngml-1h-1)', 'SBP (mmHg)', 'dBP (mmHg)', 'Na+(\xa0mmol/L)', 'K+(\xa0mmol/L)', 'ever_Hyperthyroidism_0', 'ever_Hyperthyroidism_1', 'ever_COPD_0', 'ever_COPD_1', 'ever diabetics_0', 'ever diabetics_1', 'ever_myocardiac infarction_0', 'ever_myocardiac infarction_1', 'Sex_1', 'Sex_2', 'family history_1', 'family history_2', 'total categories of antiHTN drugs_0', 'total categories of antiHTN drugs_1', 'total categories of antiHTN drugs_2', 'total categories of antiHTN drugs_3', 'total categories of antiHTN drugs_4']

Factor_149:

['age', 'Body weight(kg)', 'height (cm)', 'hypertension duration (years)', 'Aldosterone (ng/dL)', 'PRA (ngml-1h-1)', 'SBP (mmHg)', 'dBP (mmHg)', 'Na+(\xa0mmol/L)', 'K+(\xa0mmol/L)', 'ever_Hyperthyroidism_0', 'ever_Hyperthyroidism_1', 'ever_COPD_0', 'ever_COPD_1', 'ever diabetics_0', 'ever diabetics_1', 'ever_myocardiac infarction_0', 'ever_myocardiac infarction_1', 'Sex_1', 'Sex_2', 'family history_1', 'family history_2', 'total categories of antiHTN drugs_0', 'total categories of antiHTN drugs_1', 'total categories of antiHTN drugs_2', 'total categories of antiHTN drugs_3', 'total categories of antiHTN drugs_5']

Factor_150:

['age', 'Body weight(kg)', 'height (cm)', 'hypertension duration (years)', 'Aldosterone (ng/dL)', 'PRA (ngml-1h-1)', 'SBP (mmHg)', 'dBP (mmHg)', 'Na+(\xa0mmol/L)', 'K+(\xa0mmol/L)', 'ever_Hyperthyroidism_0', 'ever_Hyperthyroidism_1', 'ever_COPD_0', 'ever_COPD_1', 'ever diabetics_0', 'ever diabetics_1', 'ever_myocardiac infarction_0', 'ever_myocardiac infarction_1', 'Sex_1', 'Sex_2', 'family history_1', 'family history_2', 'total categories of antiHTN drugs_0', 'total categories of antiHTN drugs_1', 'total categories of antiHTN drugs_2', 'total categories of antiHTN drugs_4', 'total categories of antiHTN drugs_5']

CodeTexte

**Reference**

Hong, K. S., M. J. Khan and M. J. Hong (2018). "Feature Extraction and Classification Methods for Hybrid fNIRS-EEG Brain-Computer Interfaces." Front Hum Neurosci **12**: 246.
